# Supplementary material for: Design, Synthesis and Biological Evaluation of Syn and Anti-like Double Warhead Quinolinones Bearing Dihydroxy Naphthalene Moiety as Epidermal Growth Factor Receptor Inhibitors with Potential Apoptotic Antiproliferative Action
Source: Molecules. 2022 Dec 10;27(24):8765. doi: 10.3390/molecules27248765 (PMC9788418; doi:10.3390/molecules27248765)
Supplement: Supplementary file 1 [file molecules-27-08765-s001.zip › molecules-2080765-supplementary.pdf]

Supporting Information

Design, synthesis, and biological evaluation of Syn and anti-like double warhead quinolinones bearing dihydroxy naphthalene moiety as EGFR inhibitors with potential apoptotic antiproliferative action

Essmat M. El-Sheref[1*], Mohamed A. Ameen[1*], Kamal M. El-Shaieb[1], Fathy F. Abdel-Latif[1], Asmaa I. Abdel-naser[1], Alan B. Brown[2], S. Bräse[3*], Hazem M. Fathy[4], Iqrar Ahmad[5], Harun Patel[5], Hesham A.M. Gomaa[6], Bahaa G.M. Youssif[7*], Asmaa H. Mohamed[1]

[1]Chemistry Department, Faculty of Science, Minia University, El Minia, 61519 Egypt; [2]Chemistry Department, Florida Institute of Technology, 150 W University Blvd, Melbourne, FL 32901 USA; [3]Institute of Biological and Chemical Systems, IBCS-FMS, Karlsruhe Institute of Technology, 76131 Karlsruhe, Germany; [4]Pharmacetical Organic Chemistry Department, Faculty of Pharmacy, Al-Azhar University, Assiut Branch, Assiut, 71524 Egypt; [5]Division of Computer Aided Drug Design, Department of Pharmaceutical Chemistry, R. C. Patel Institute of Pharmaceutical Education and Research, Shirpur, Maharashtra, 425405 India; [6]Pharmacology Department, College of Pharmacy, Jouf University, Sakaka, 72314 Saudi Arabia; [7]Pharmaceutical Organic Chemistry Department, Faculty of Pharmacy, Assiut University, Assiut, 71526 Egypt.

Spectral data for compound 4a.

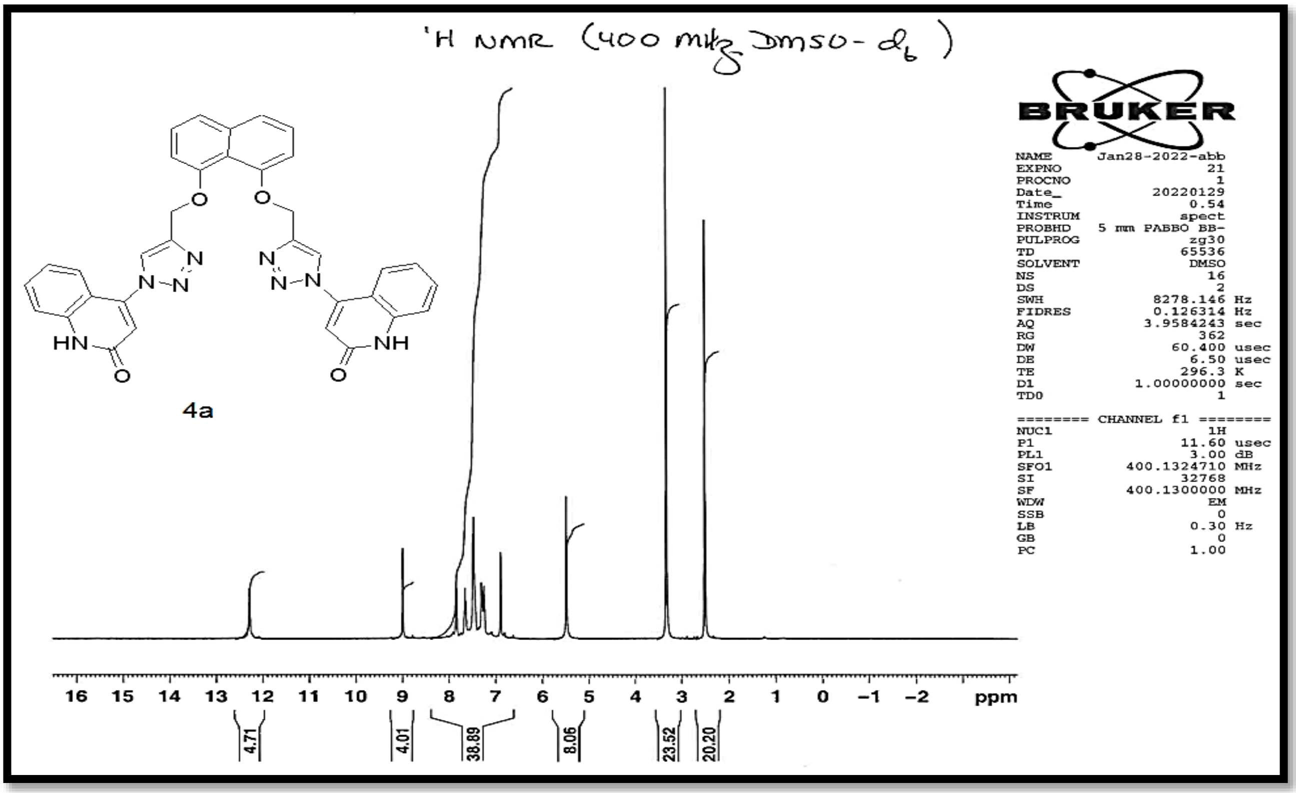

Figure S1. ^{1}H-NMR spectrum for compound **4a.**

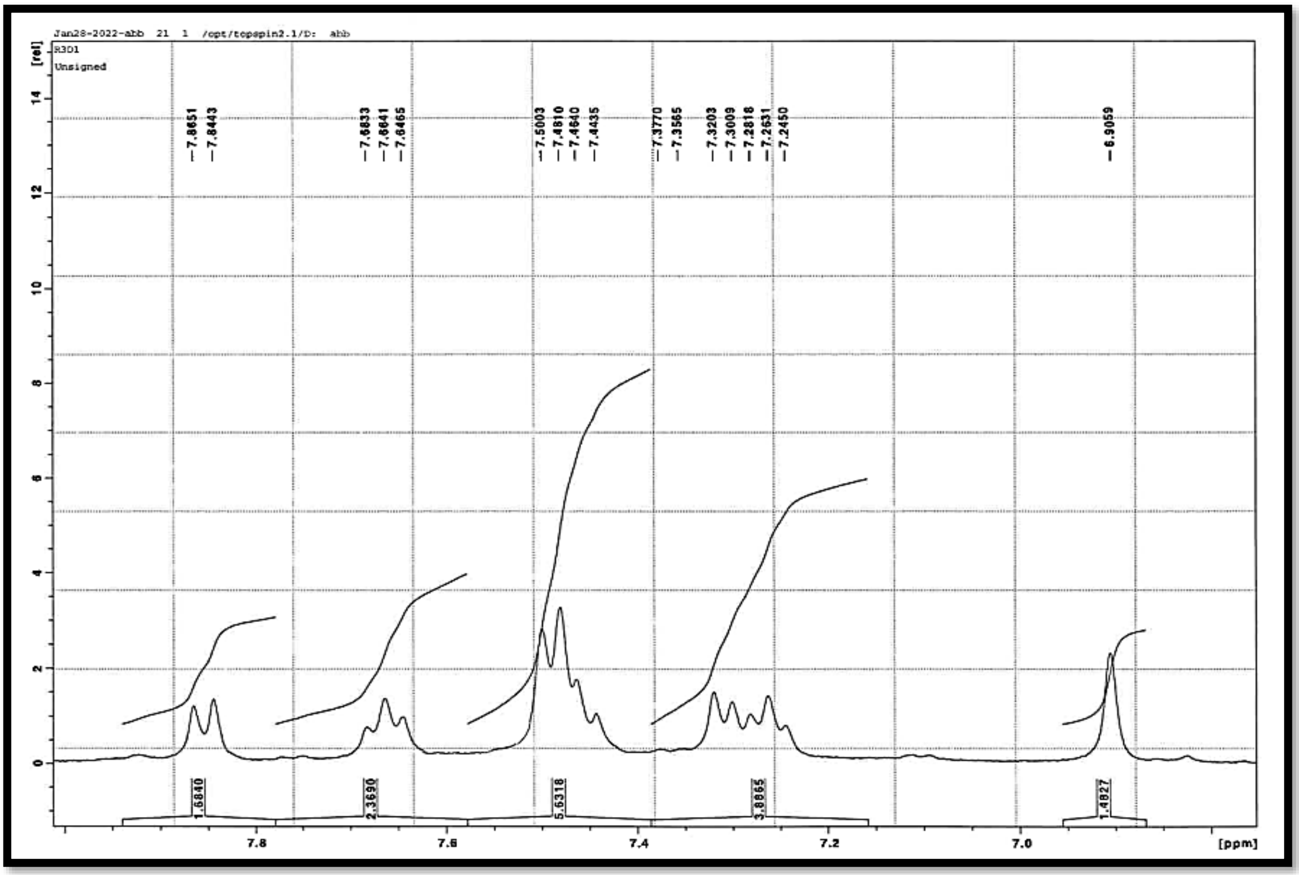

Figure S2. Part of the ^{1}H-NMR spectrums for compound **4a.**

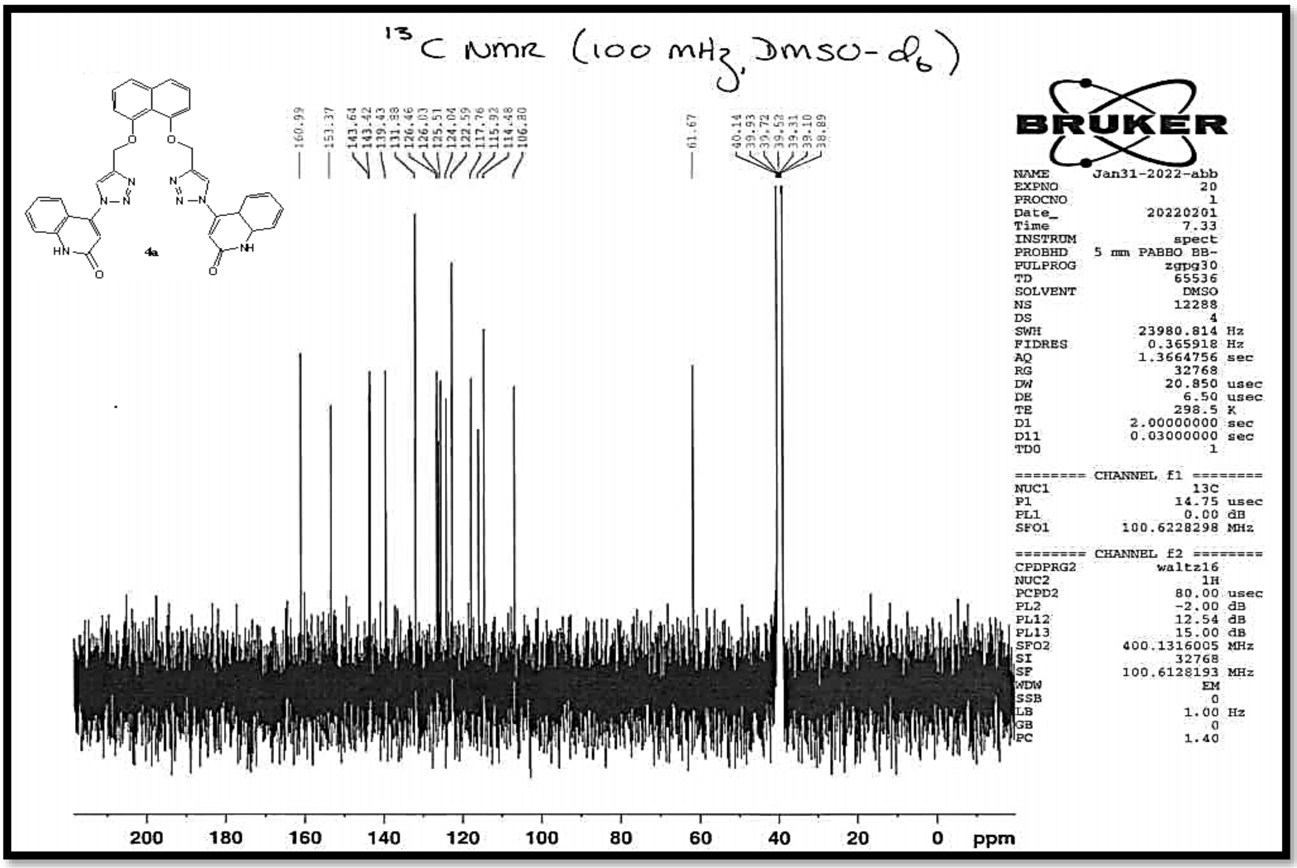

Figure S3. ^{13}C-NMR spectrums for compound **4a.**

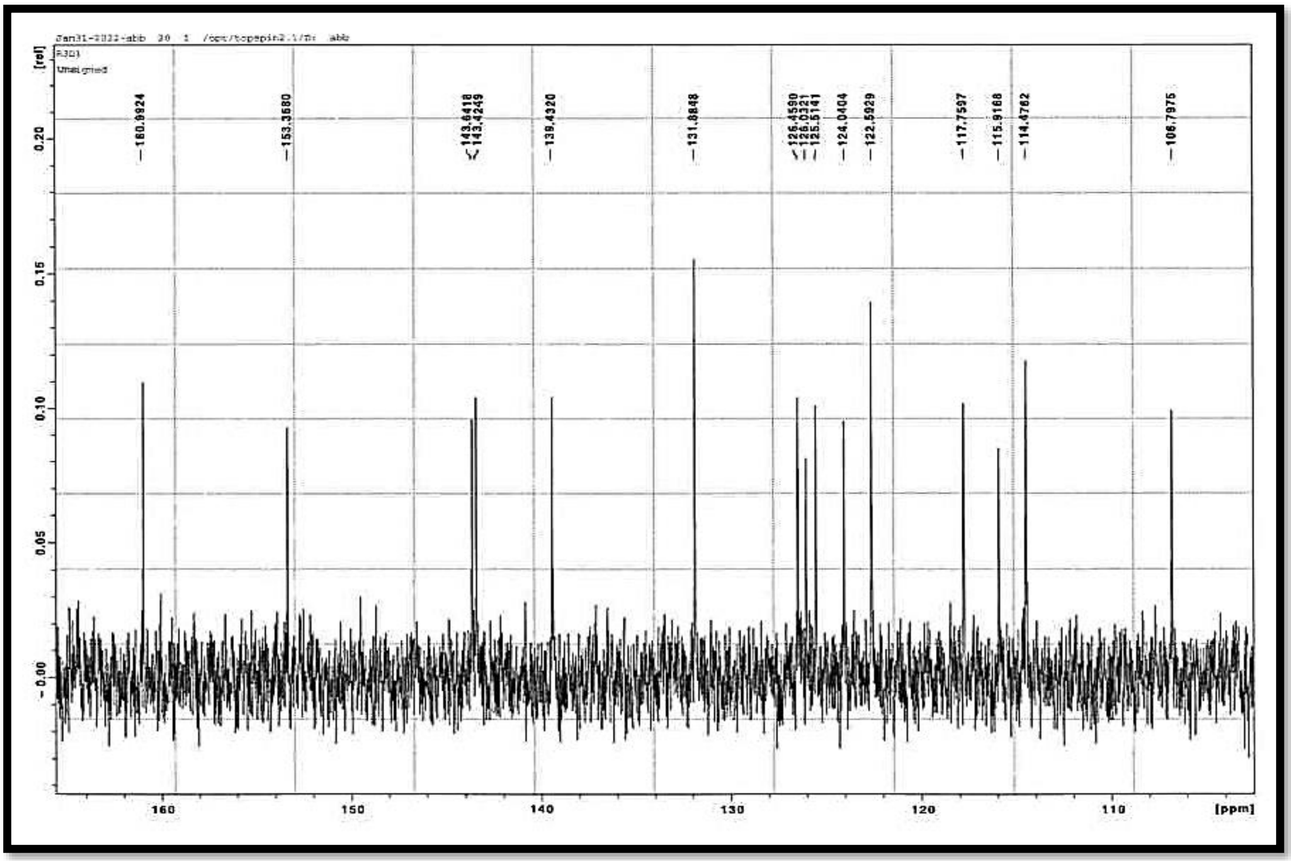

Figure S4. Part of the ^{13}C-NMR spectrums for compound **4a.**

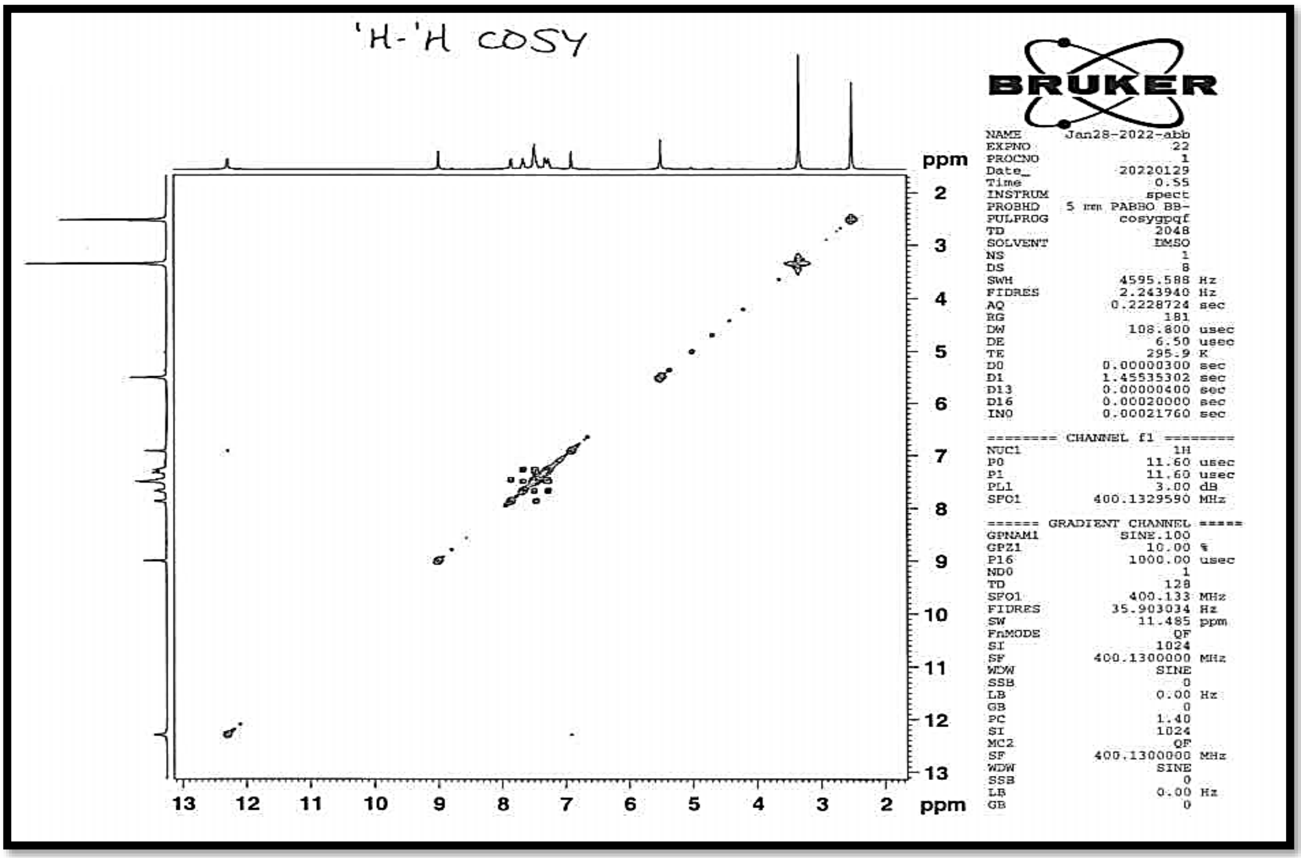

Figure S5. ^{1}H-^{1}H Cosy spectrums for compound **4a.**

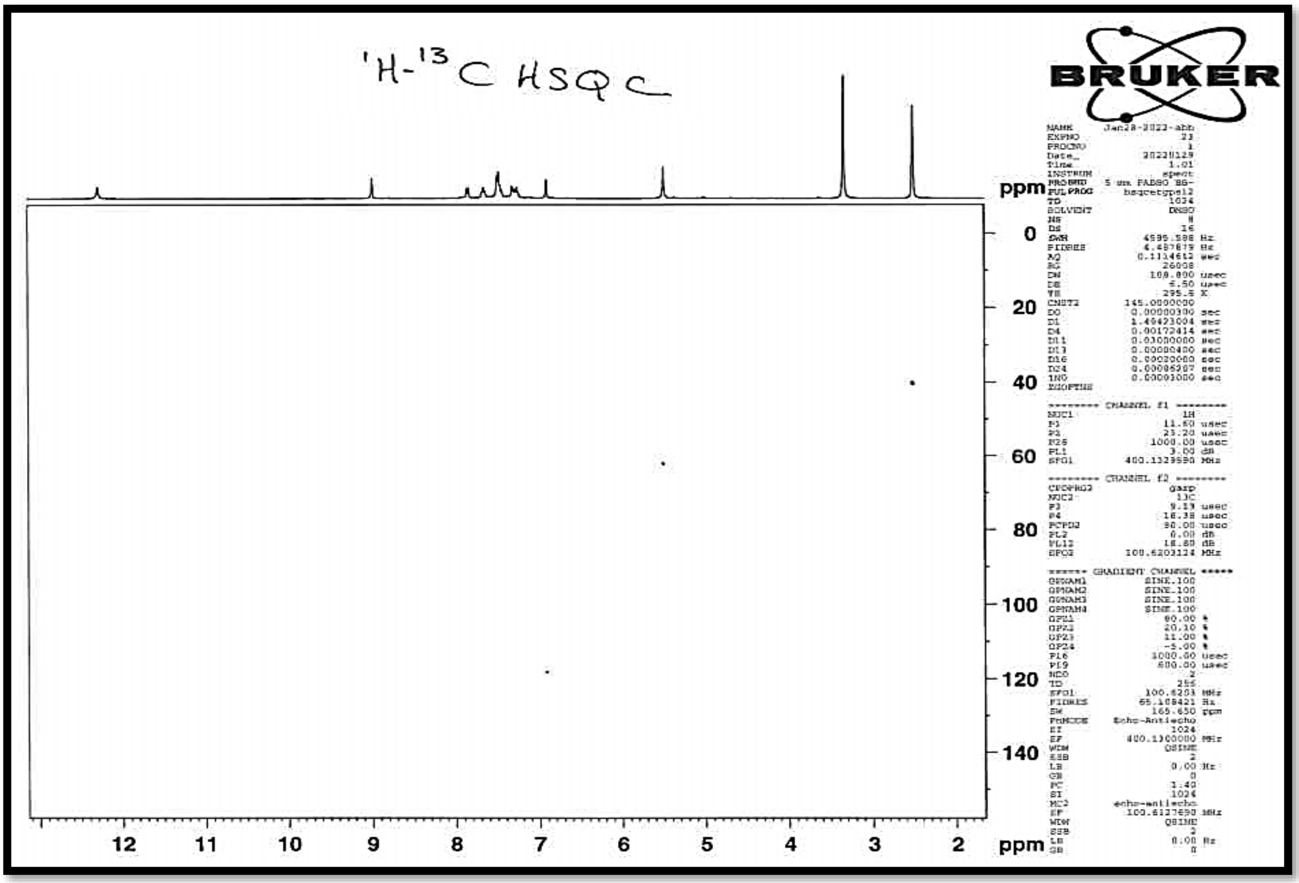

Figure S6. ^{1}H-^{13}C HSQC spectrums for compound **4a.**

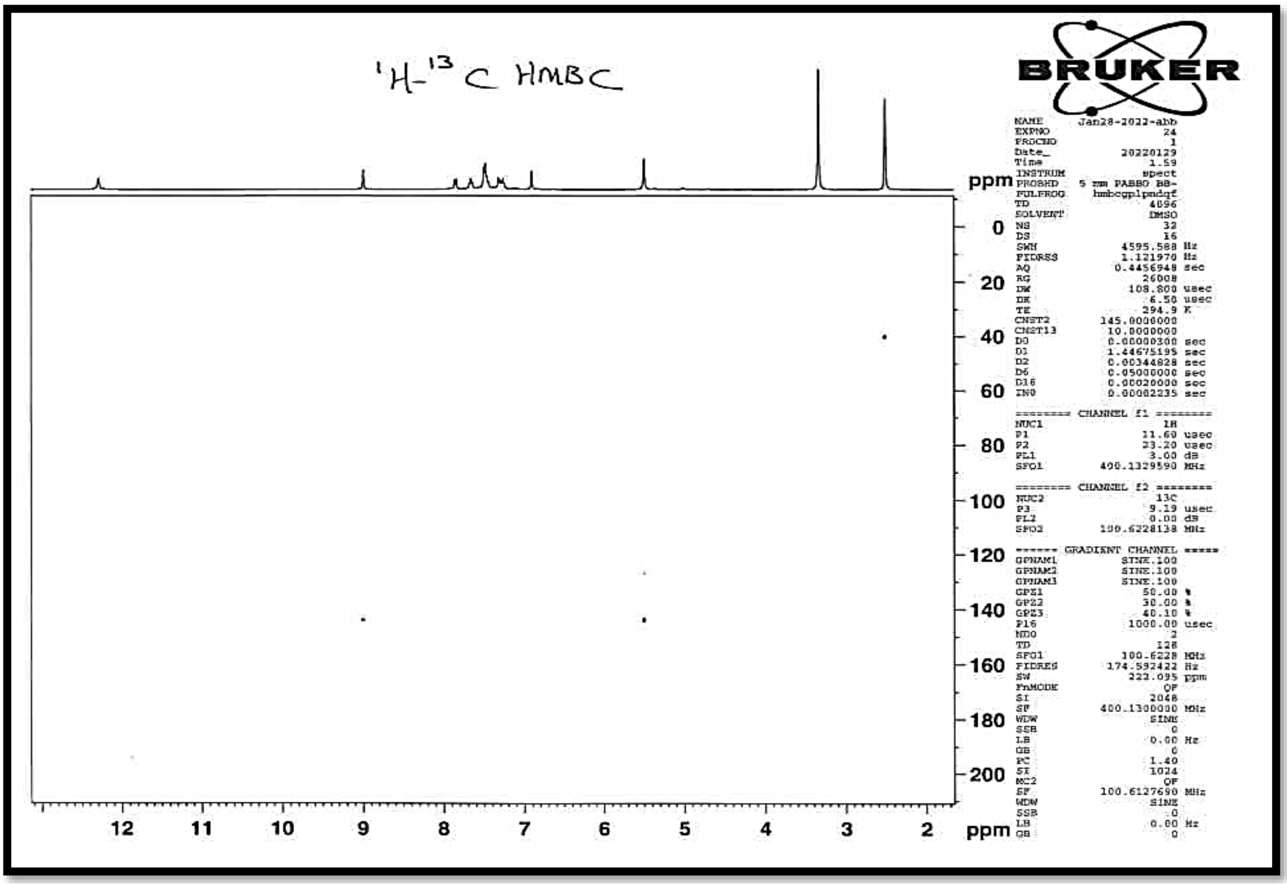

Figure S7. ^{1}H-^{13}C HMBC spectrums for compound **4a.**

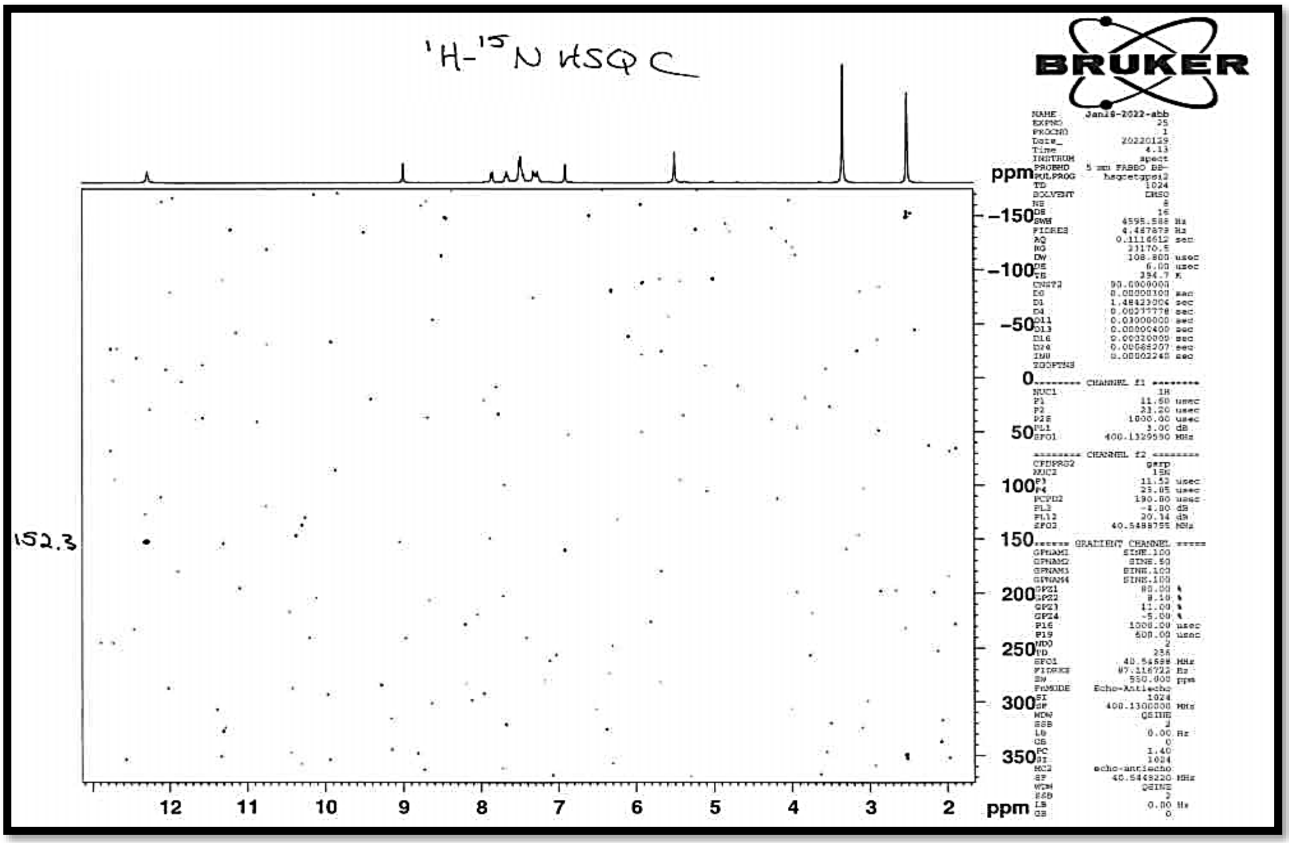

Figure S8. ^{1}H-^{15}N HSQC spectrums for compound **4a.**

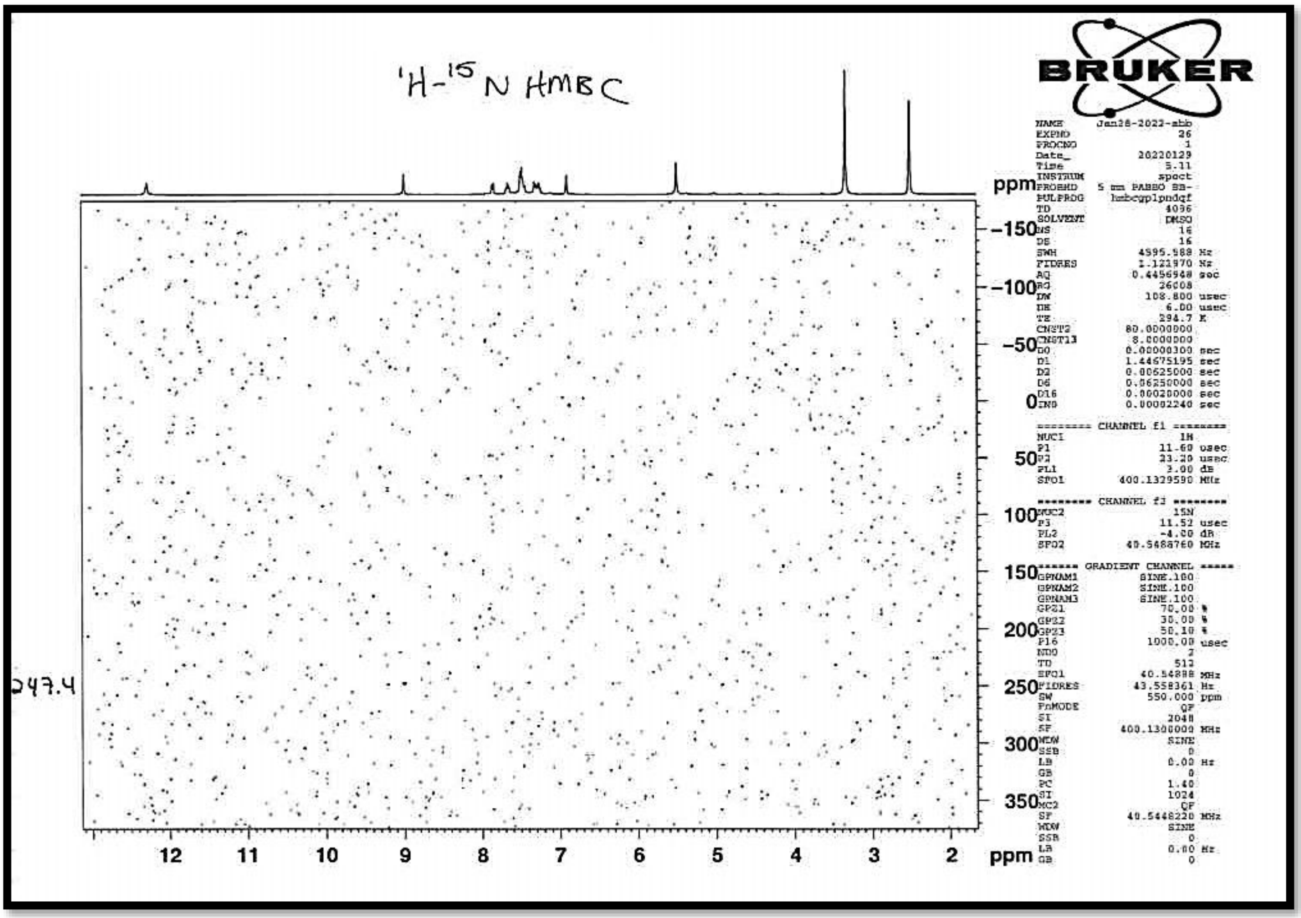

Figure S9. ^{1}H-^{15}N HMBC spectrums for compound **4a.**

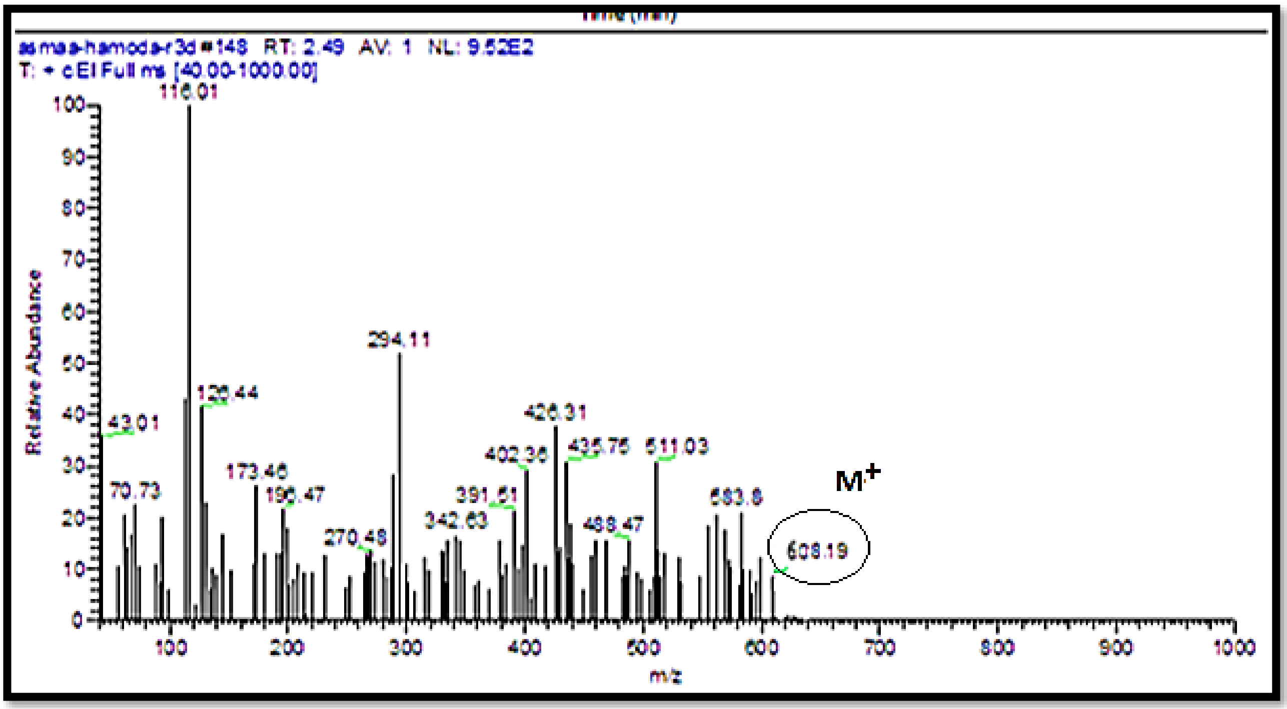

Figure S10. Mass spectrometry spectrums for compound **4a.**

Spectral data for compound 4b.

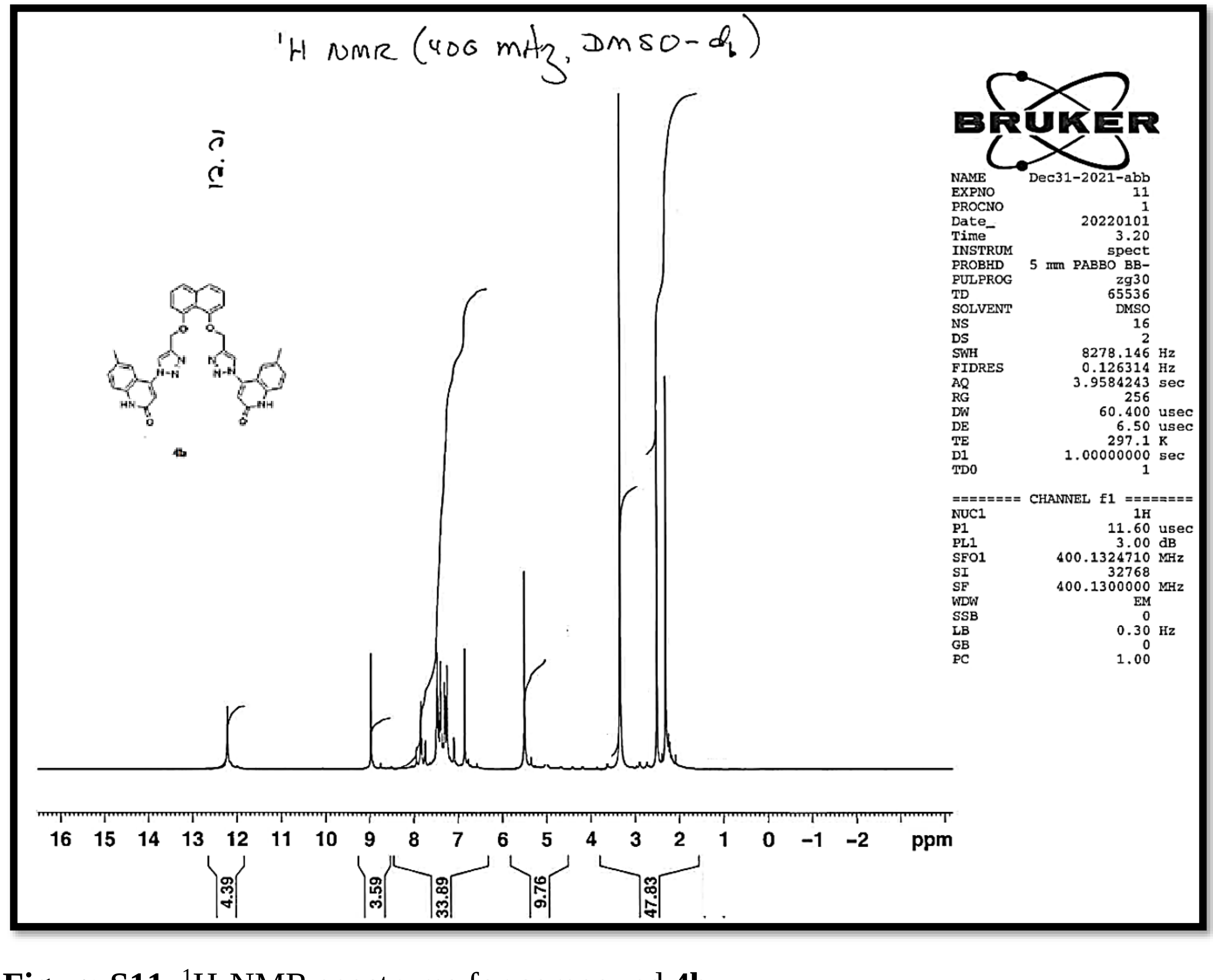

Figure S11. ^{1}H-NMR spectrums for compound **4b.**

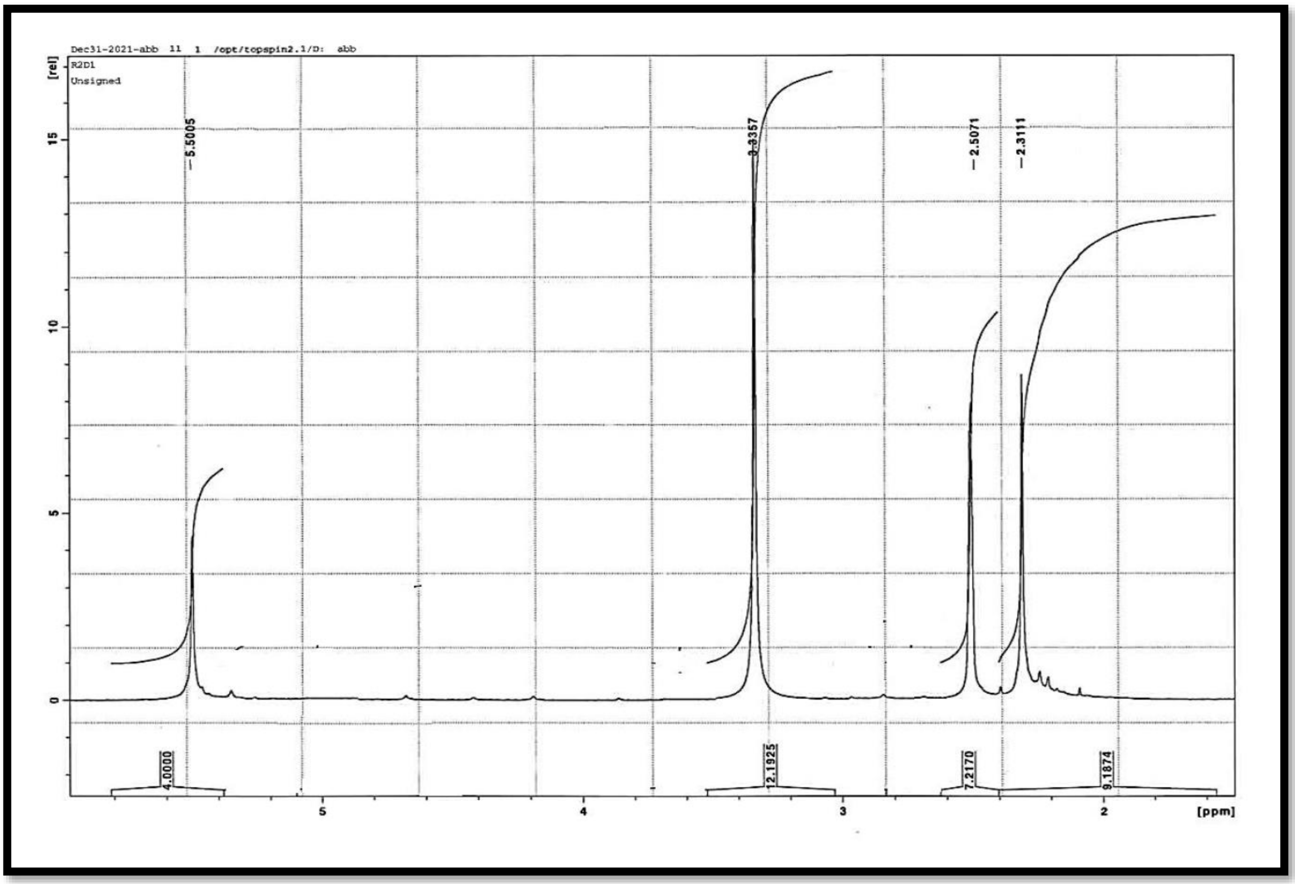

Figure S12. Part of the ^{1}H-NMR spectrums for compound **4b.**

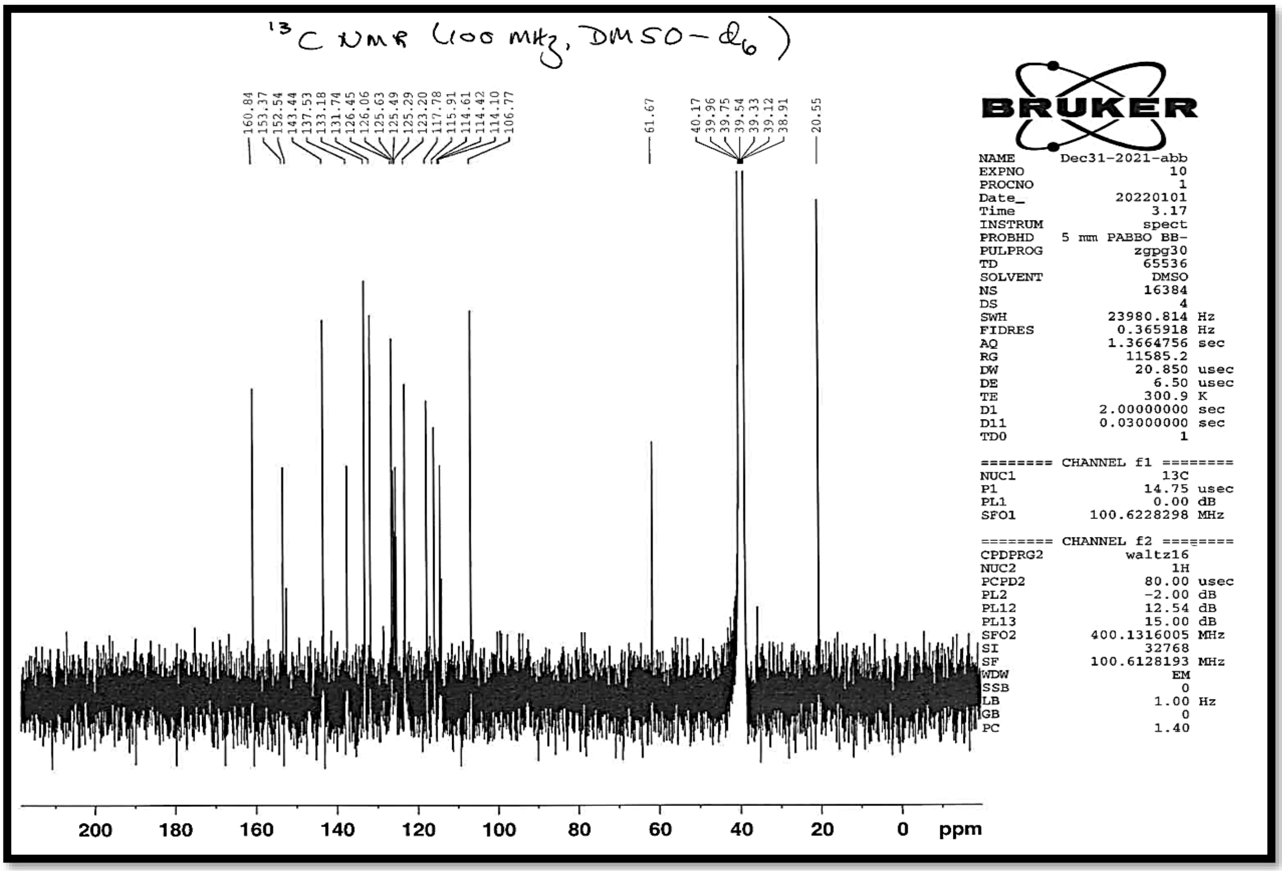

Figure S13. ^{13}C-NMR spectrums for compound **4b.**

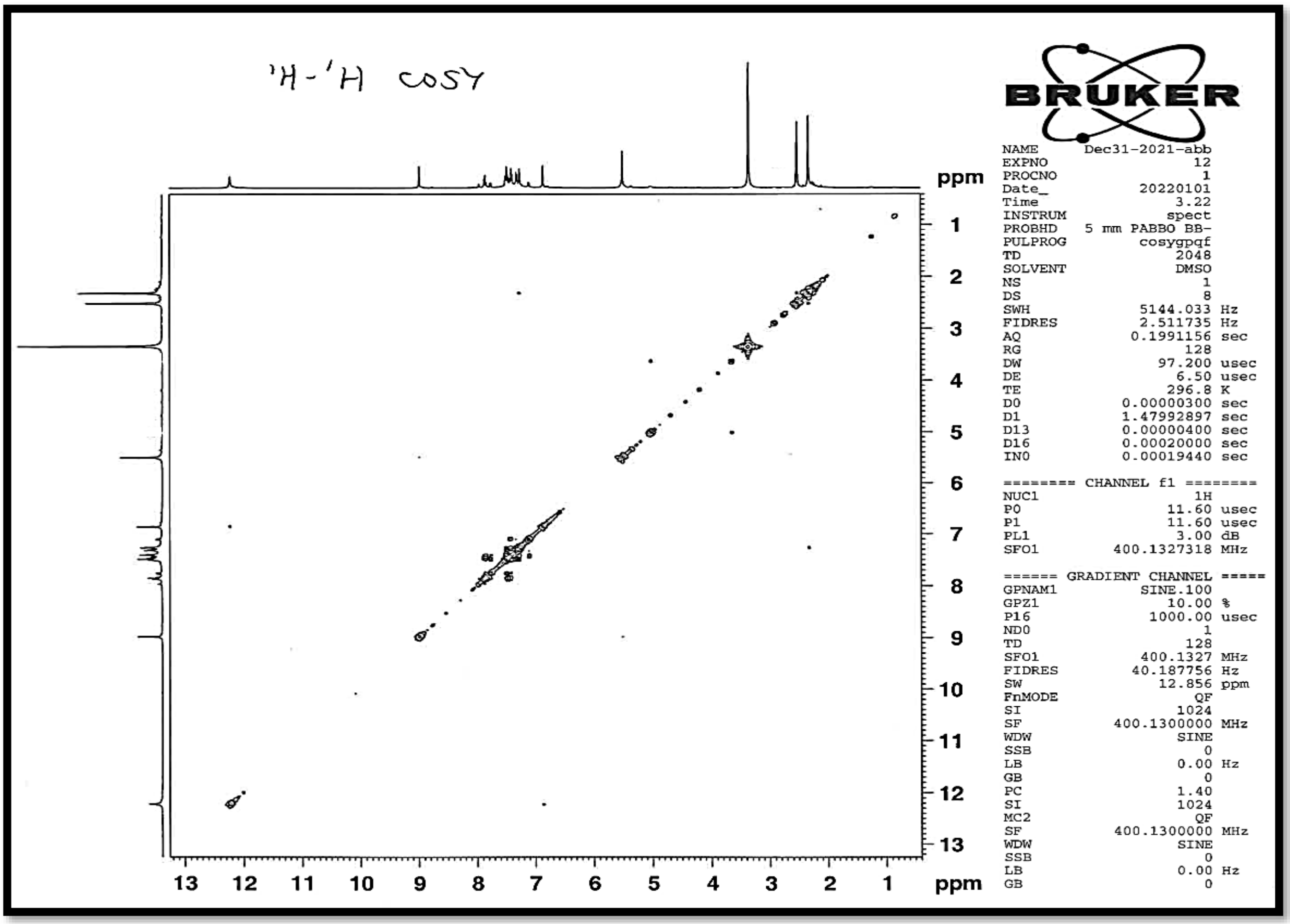

Figure S14. ^{1}H-^{1}H Cosy spectrums for compound **4b.**

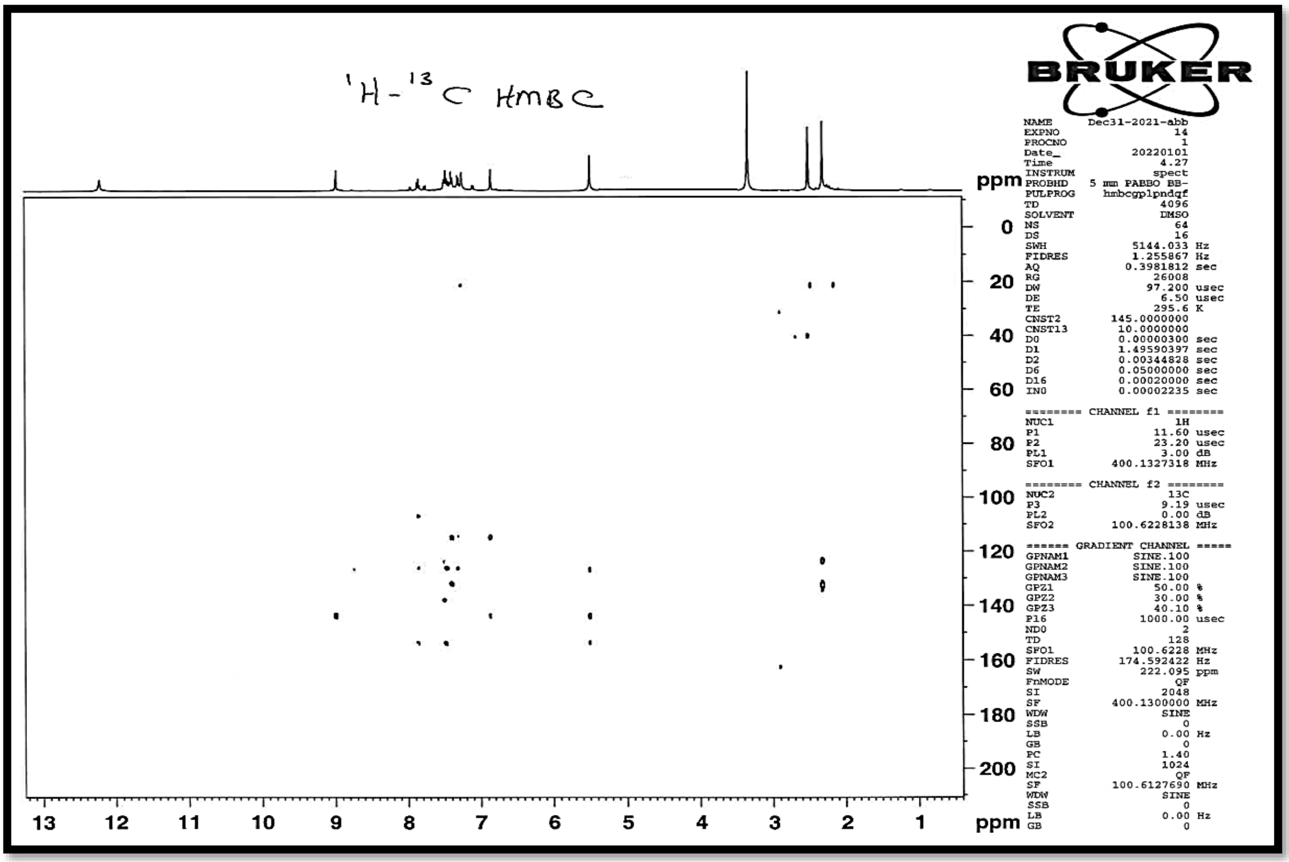

Figure S15. ^{1}H-^{13}C HMBC spectrums for compound **4b.**

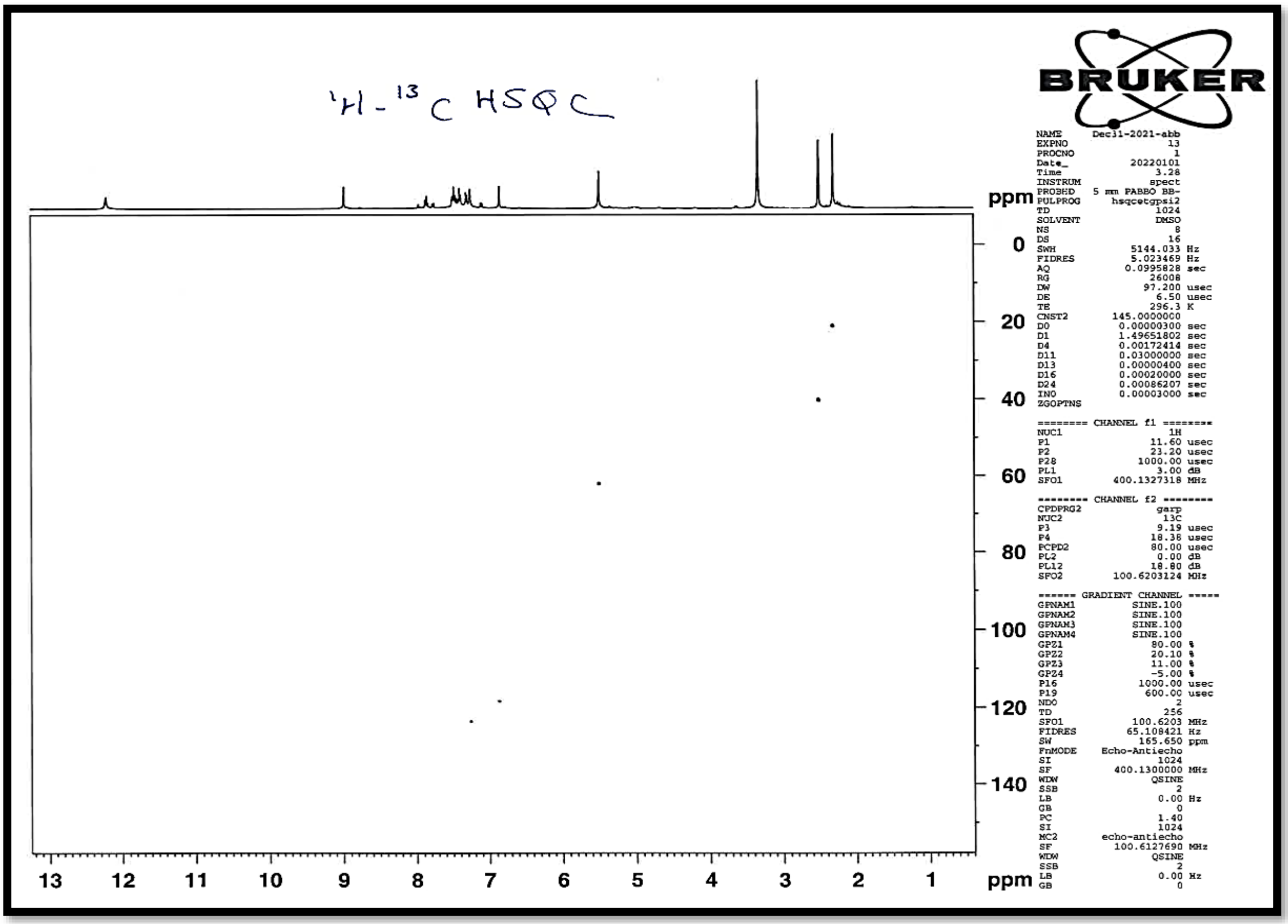

Figure S16. ^{1}H-^{13}C HSQC spectrums for compound **4b.**

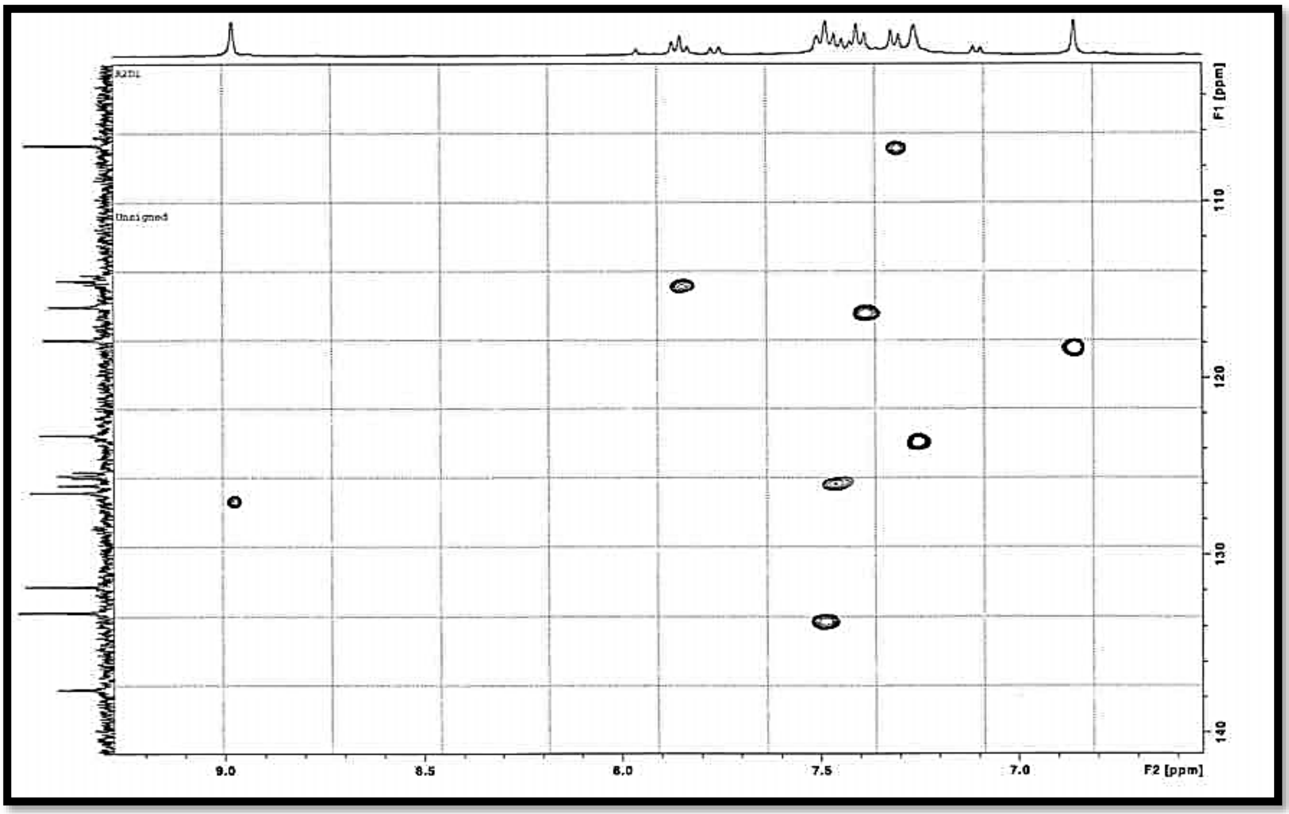

Figure S17. Part of the ^{1}H-^{13}C HSQC spectrums for compound **4b.**

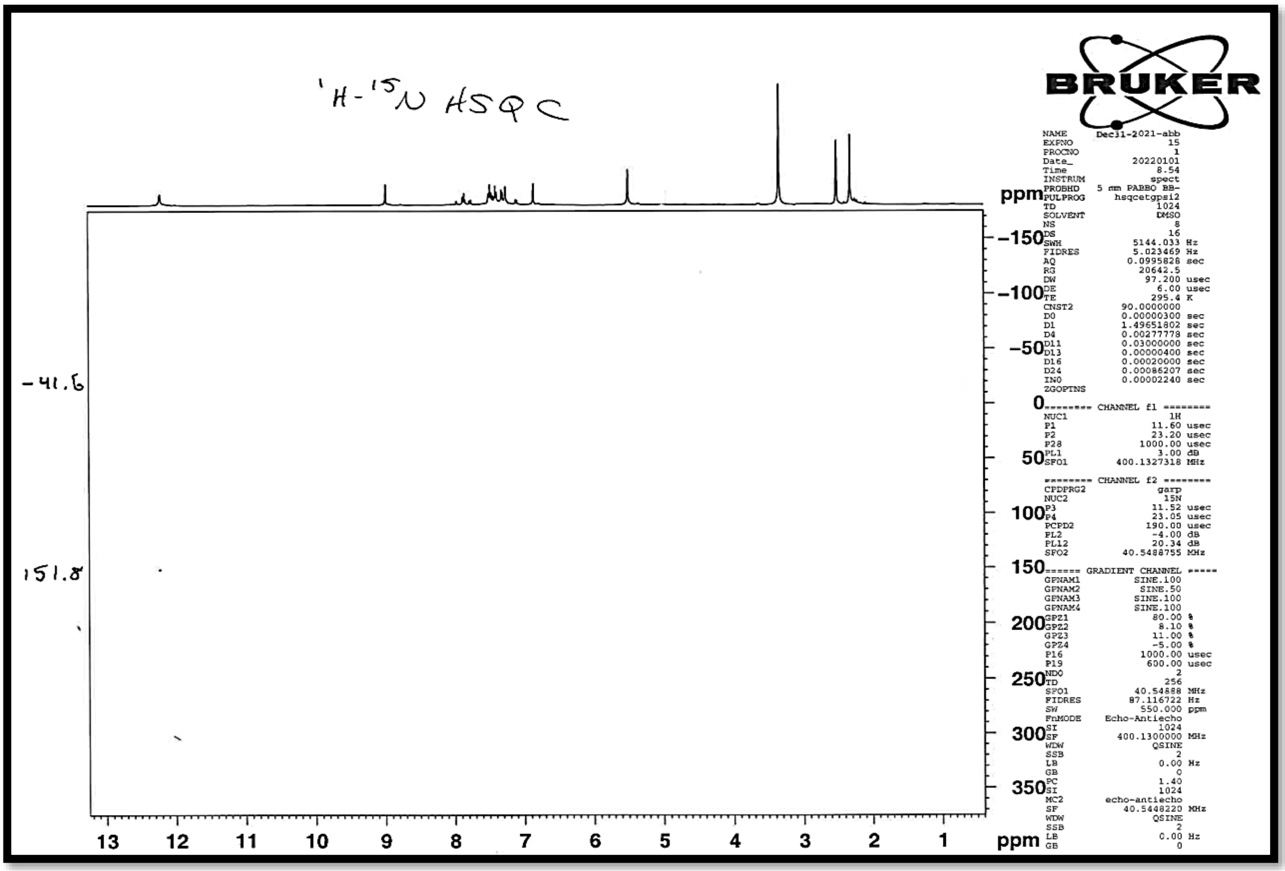

Figure S18. ^{1}H-^{15}N HSQC spectrums for compound **4b.**

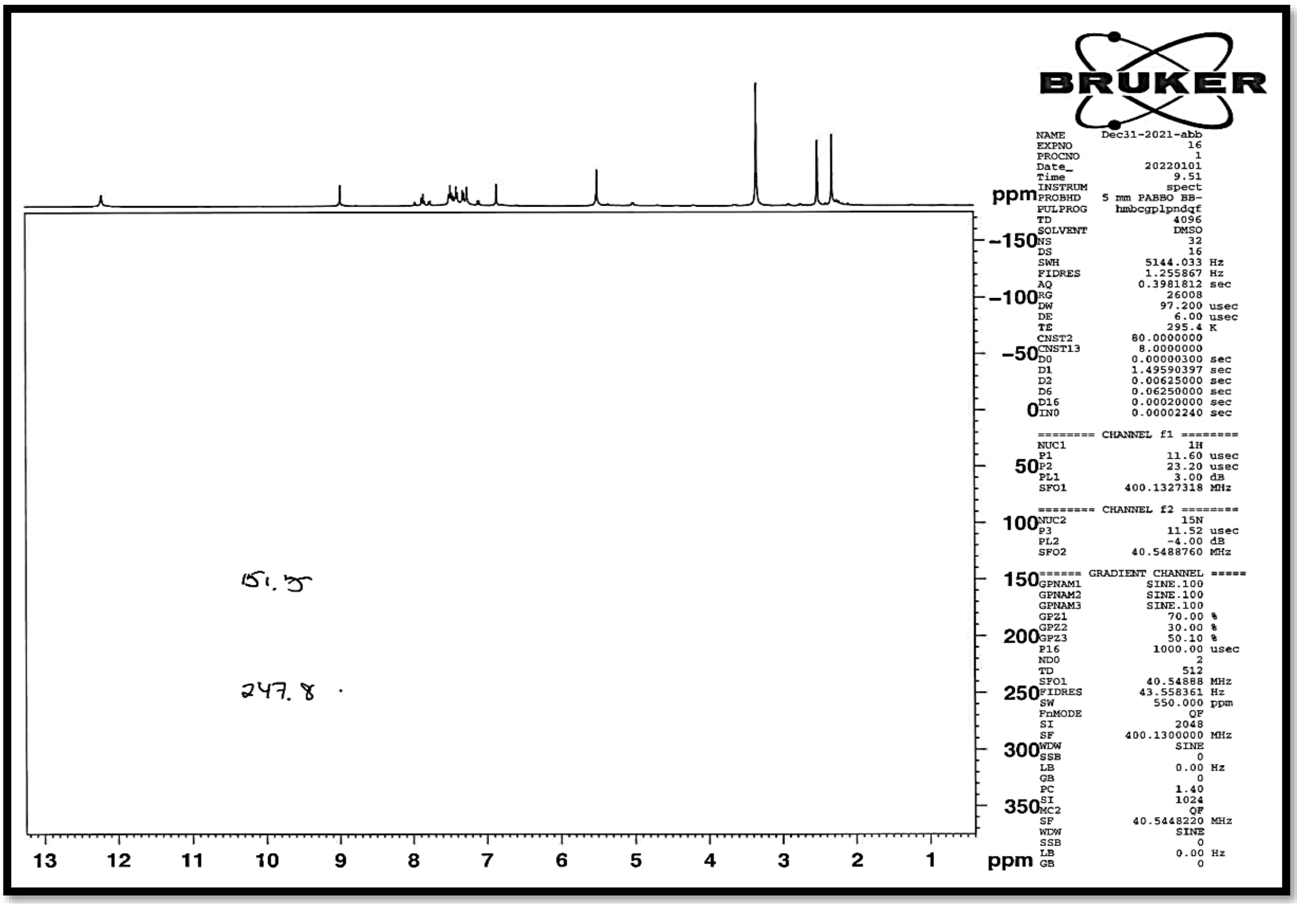

Figure S19. ^{1}H-^{15}N HMBC spectrums for compound **4b.**

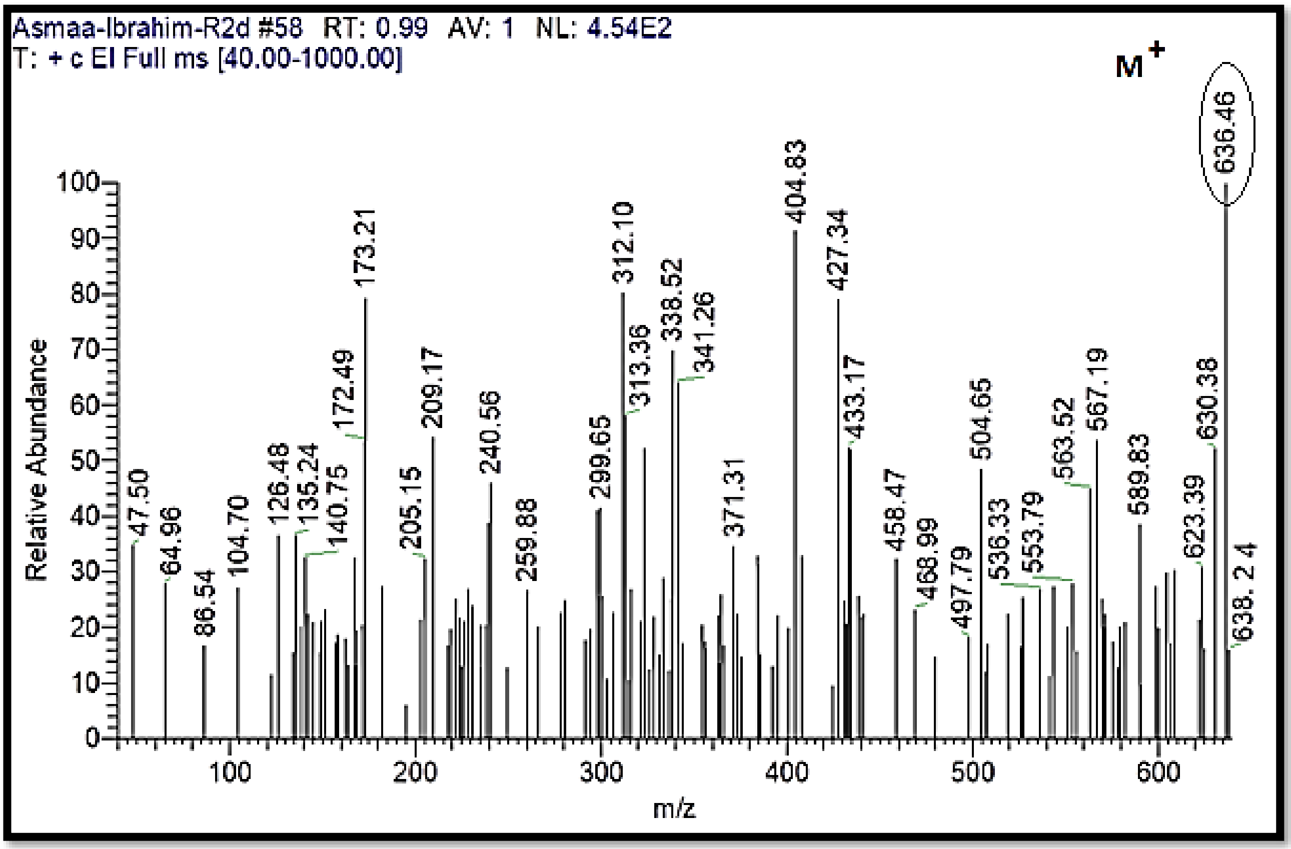

Figure S20. Mass spectrometry for compound **4b.**

Spectral data for compound 4c.

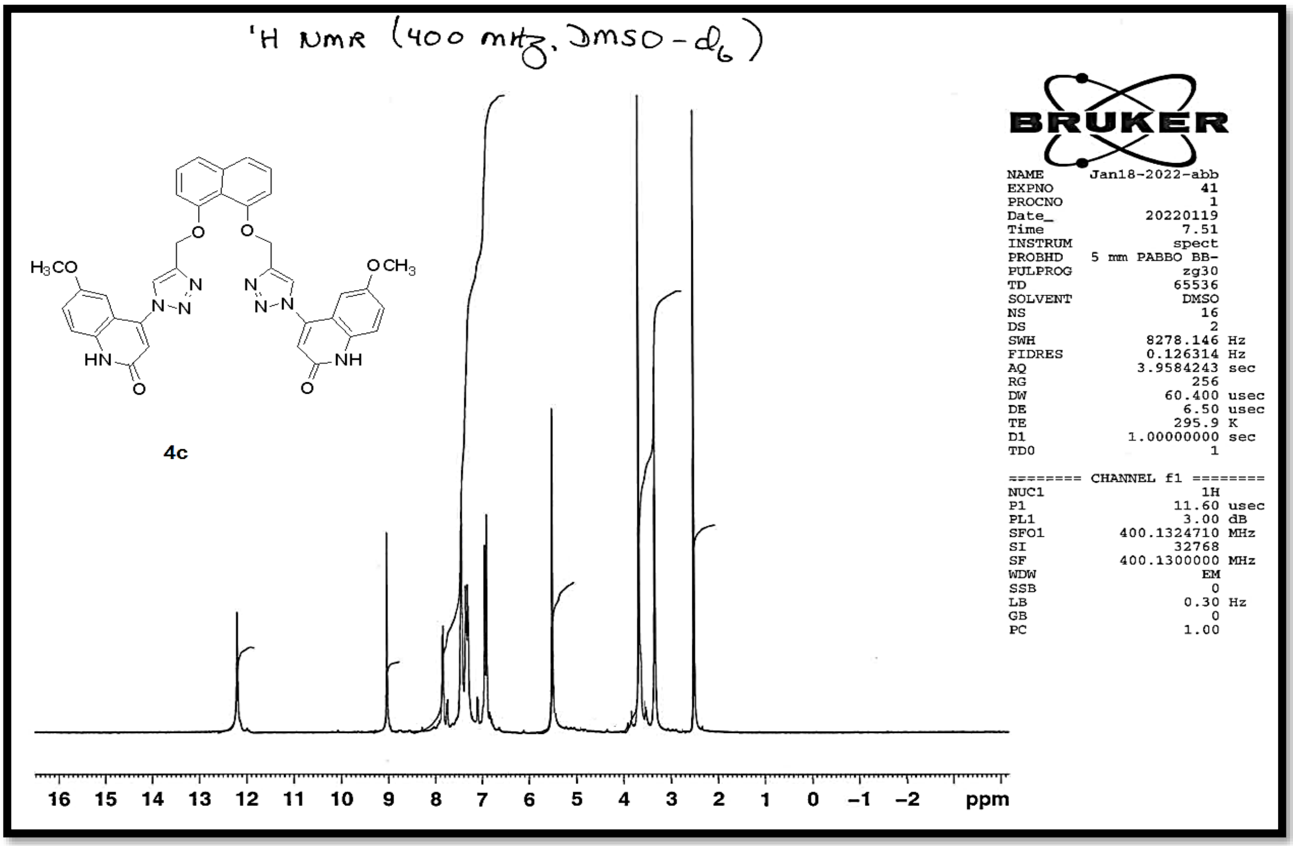

Figure S21. ^{1}H NMR spectrums for compound **4c.**

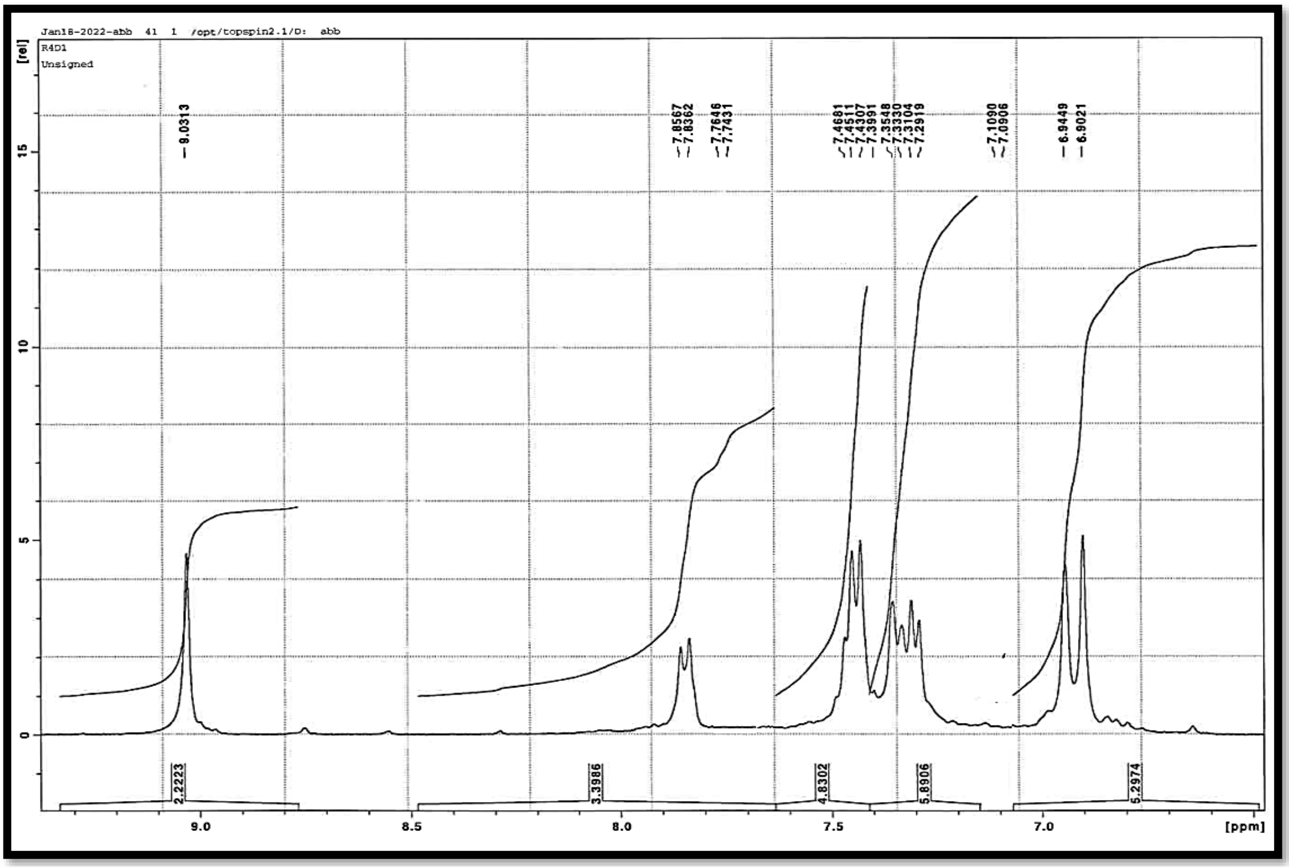

Figure S22. Part of the ^{1}H NMR spectrums for compound **4c.**

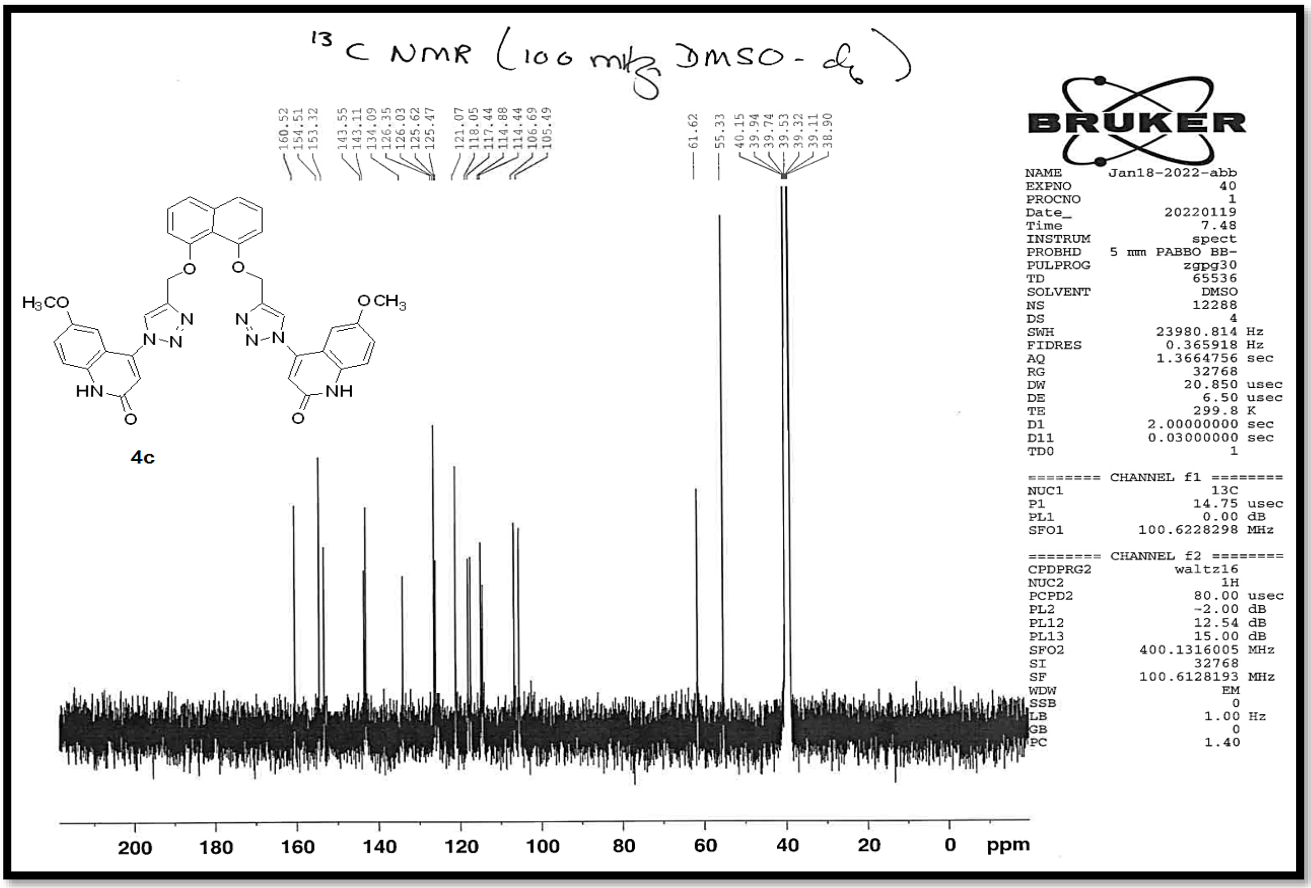

Figure S23. ^{13}C NMR spectrums for compound **4c.**

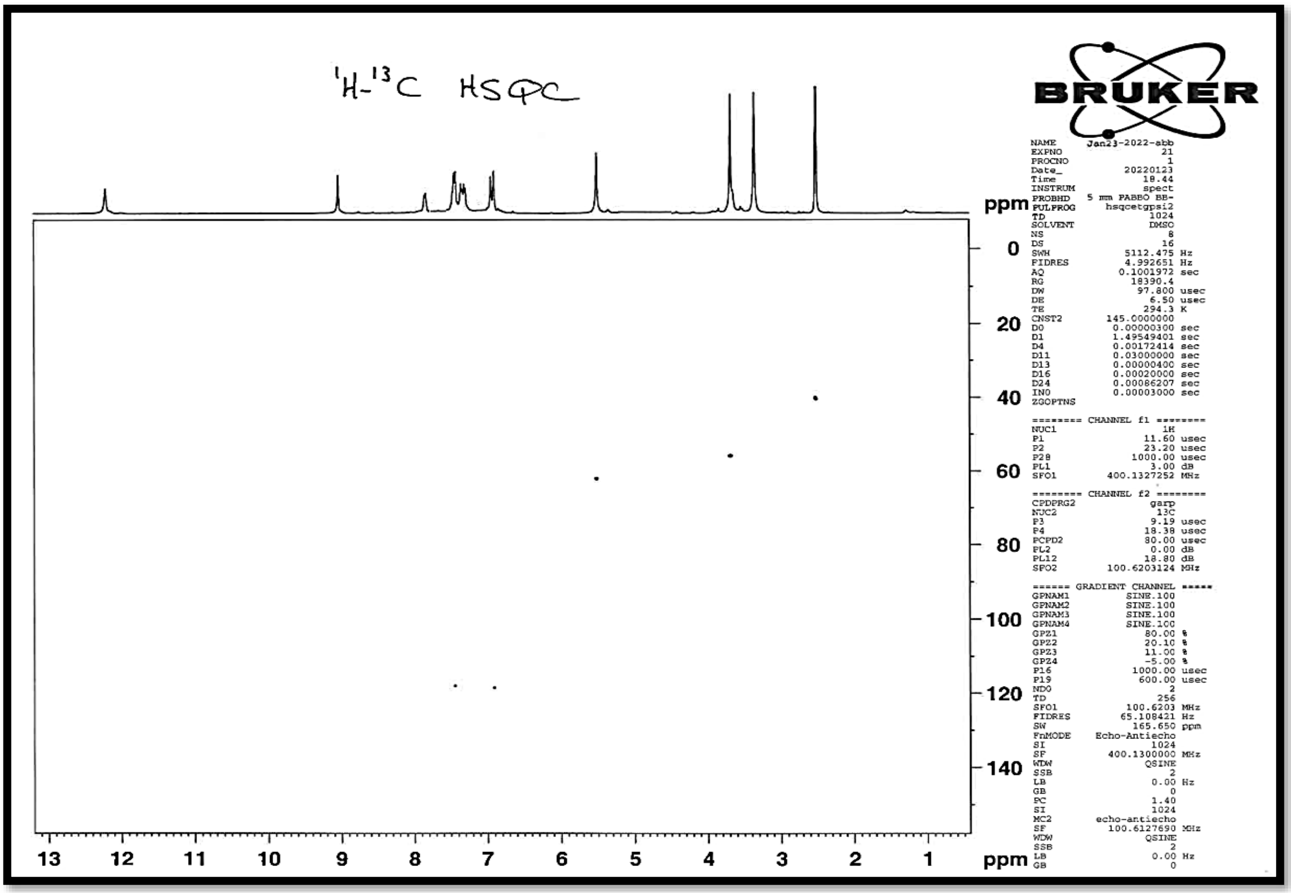

Figure S24. ^{1}H-^{13}C HSQC spectrums for compound **4c.**

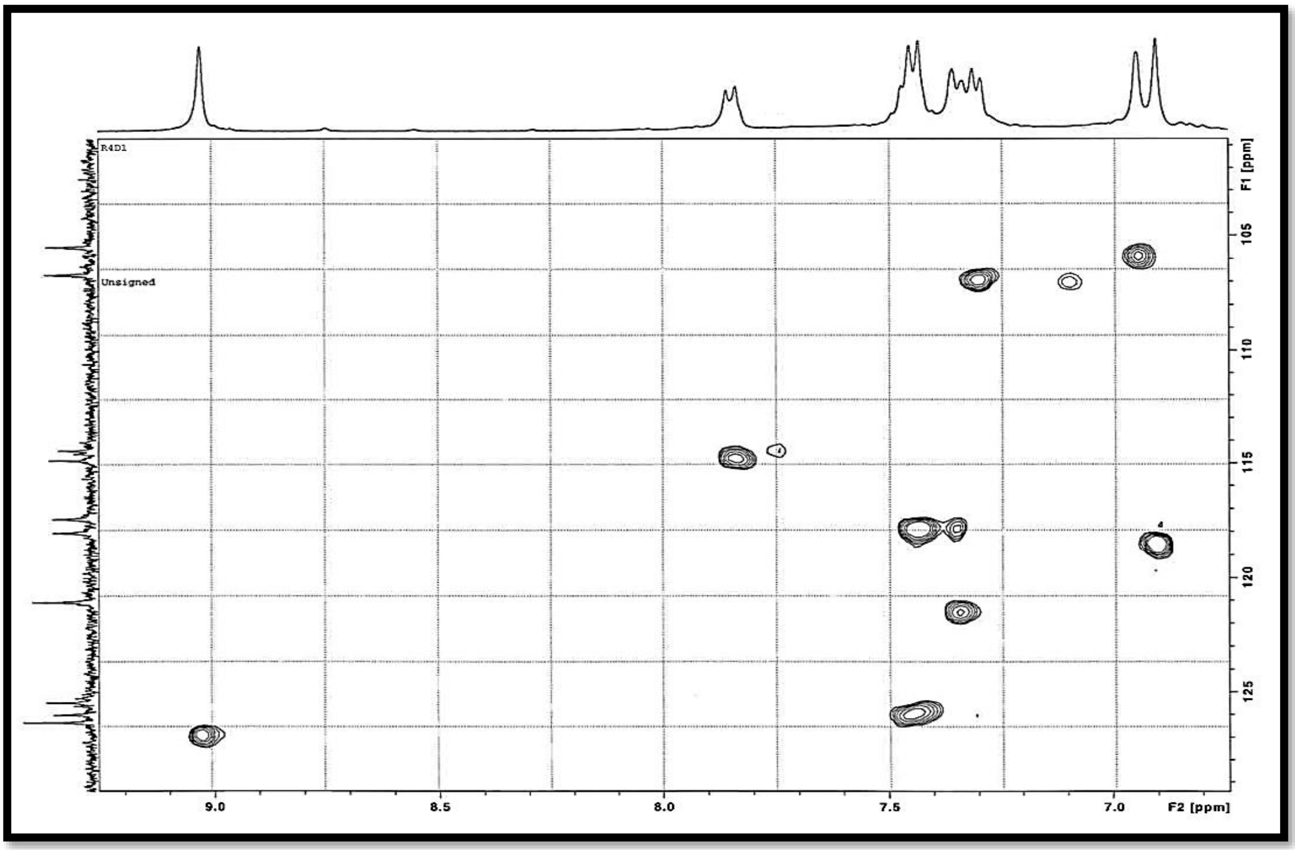

Figure S25. Part of ^{1}H-^{13}C HSQC spectrums for compound **4c.**

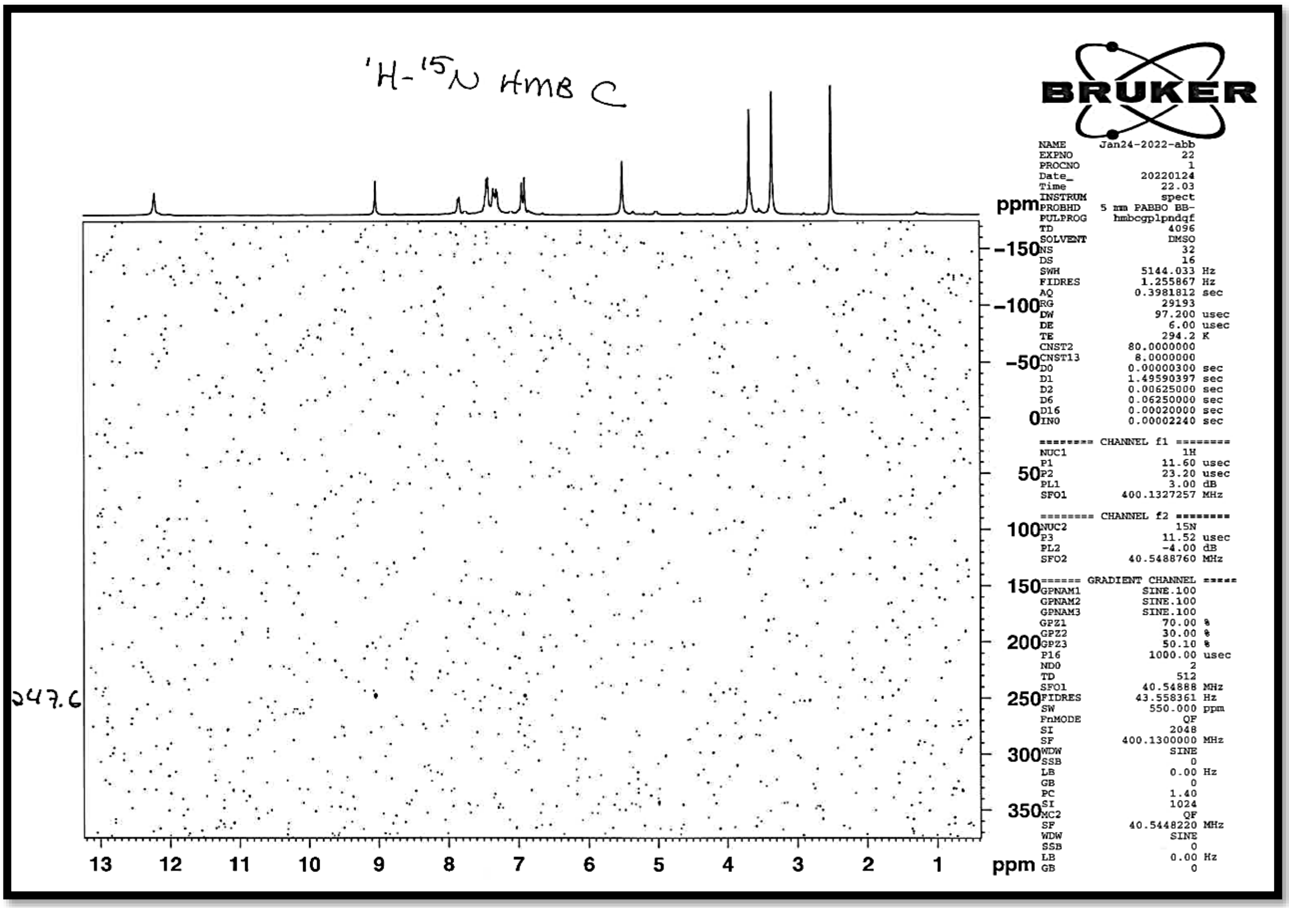

Figure S26. ^{1}H-^{15}N HMBC spectrums for compound **4c.**

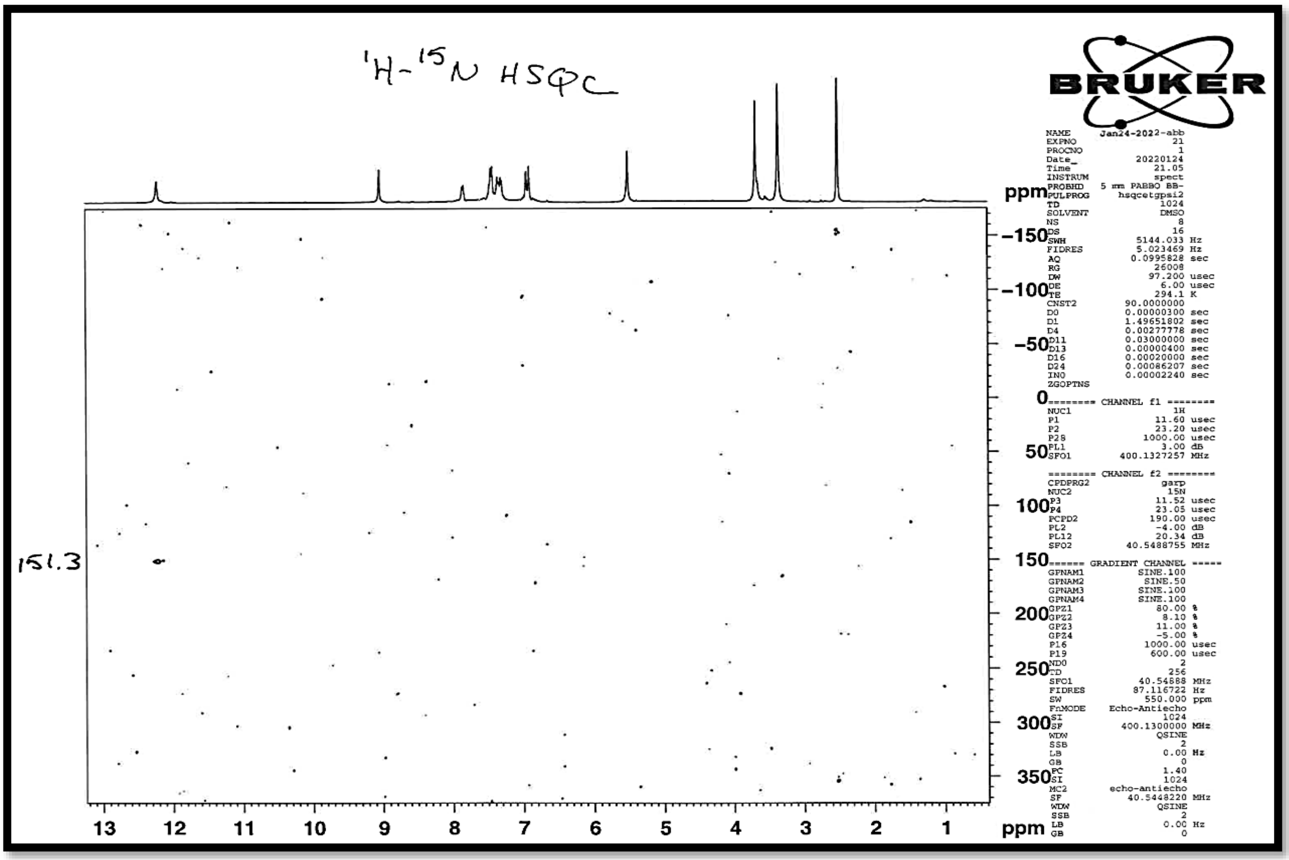

Figure S27. ^{1}H-^{15}N SQC spectrums for compound **4c.**

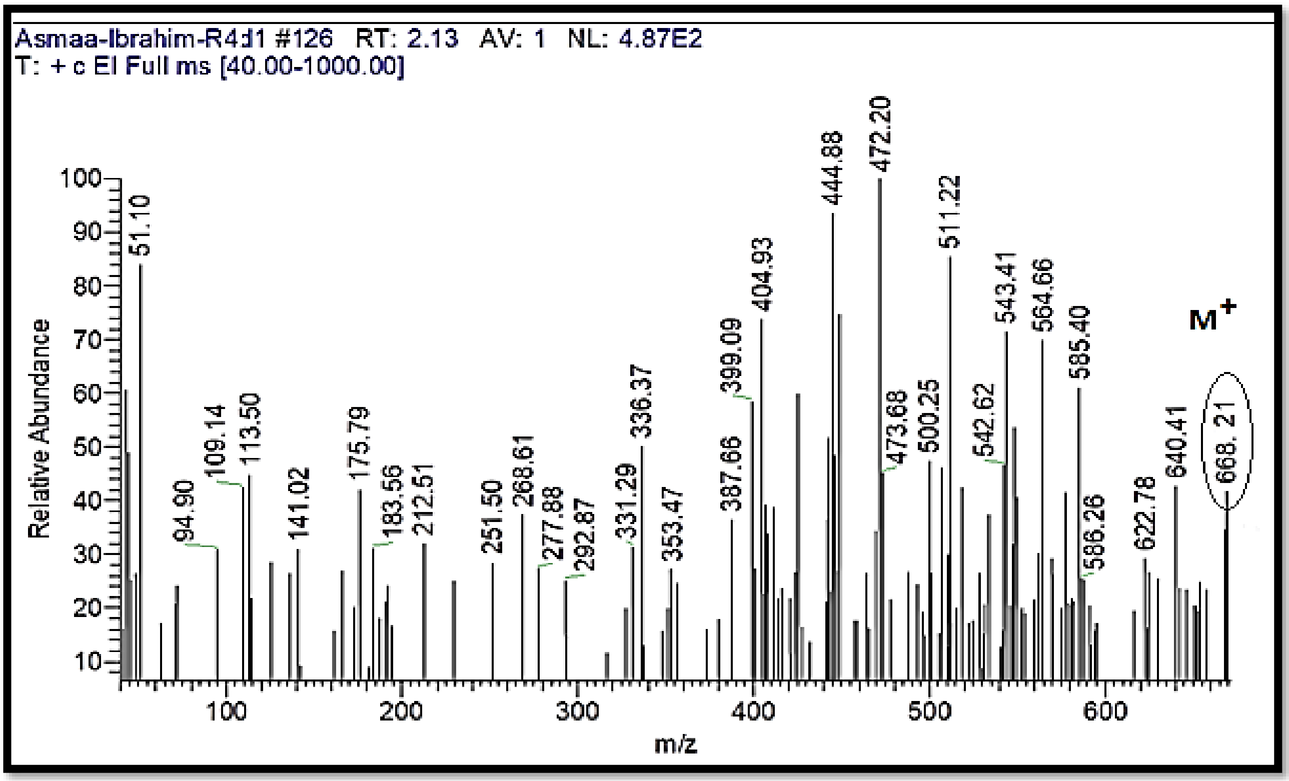

Figure S28. Mass spectrometry for compound **4c.**

Spectral data for compound 4d.

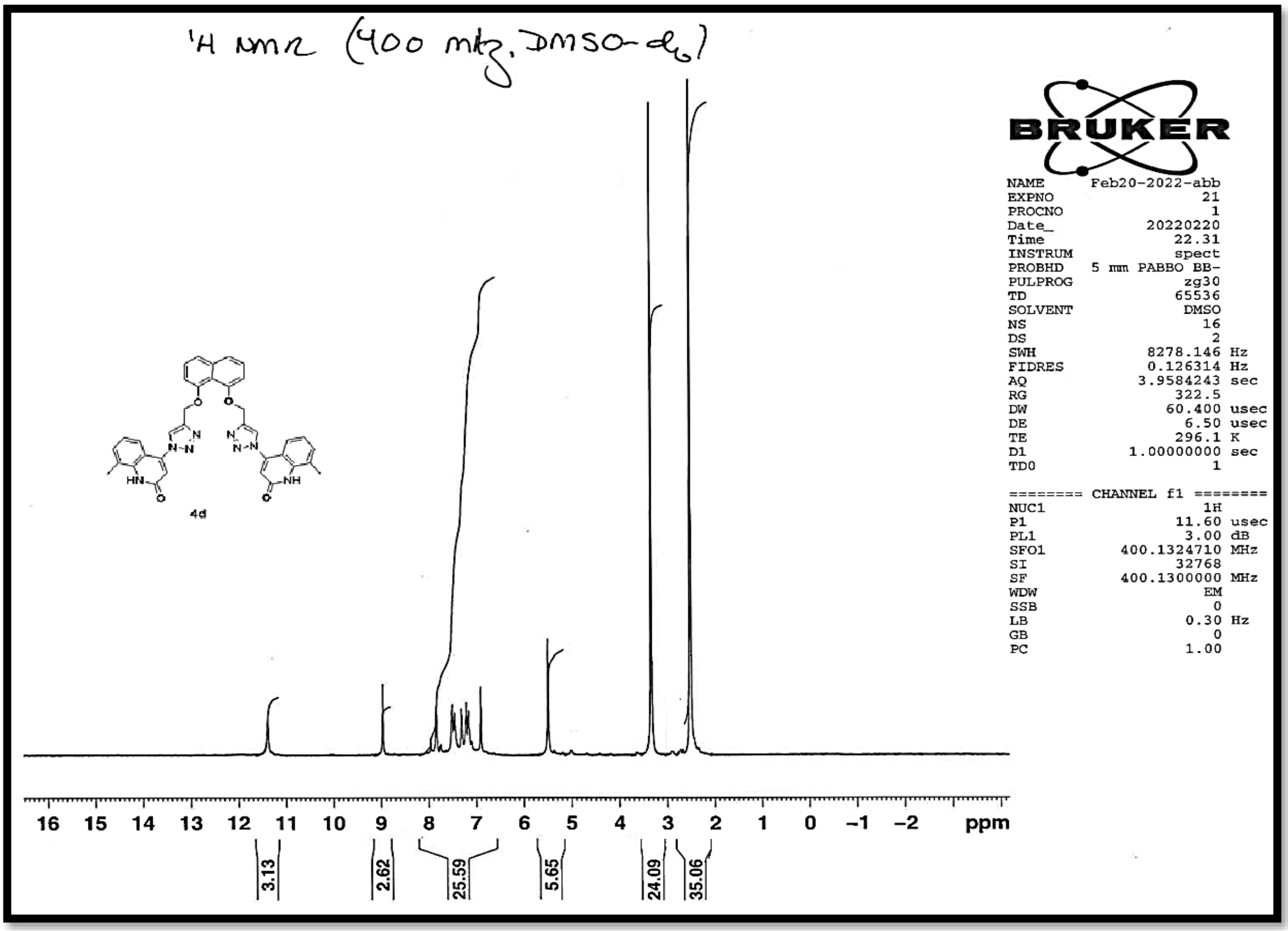

Figure S29. ^{1}H NMR spectrums for compound **4d.**

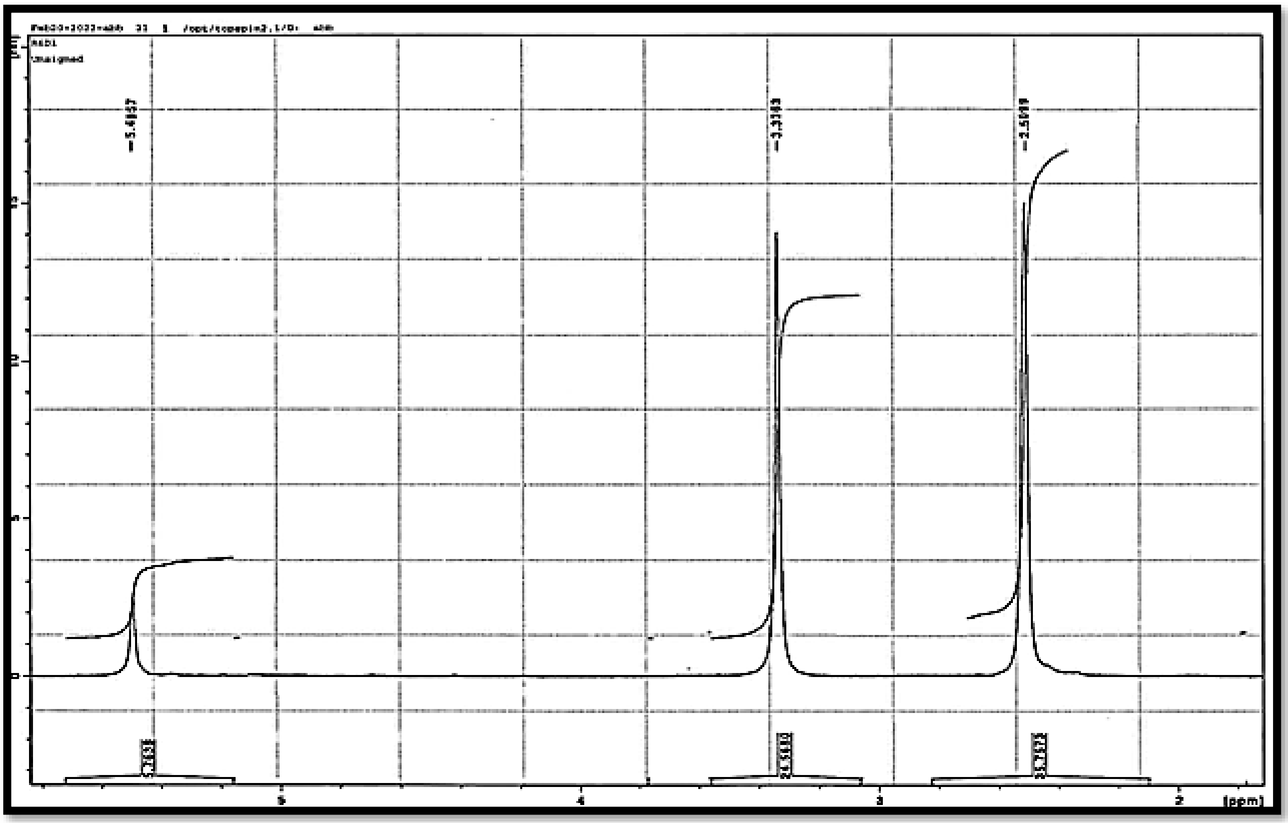

Figure S30. Part of the ^{1}H NMR spectrums for compound **4d.**

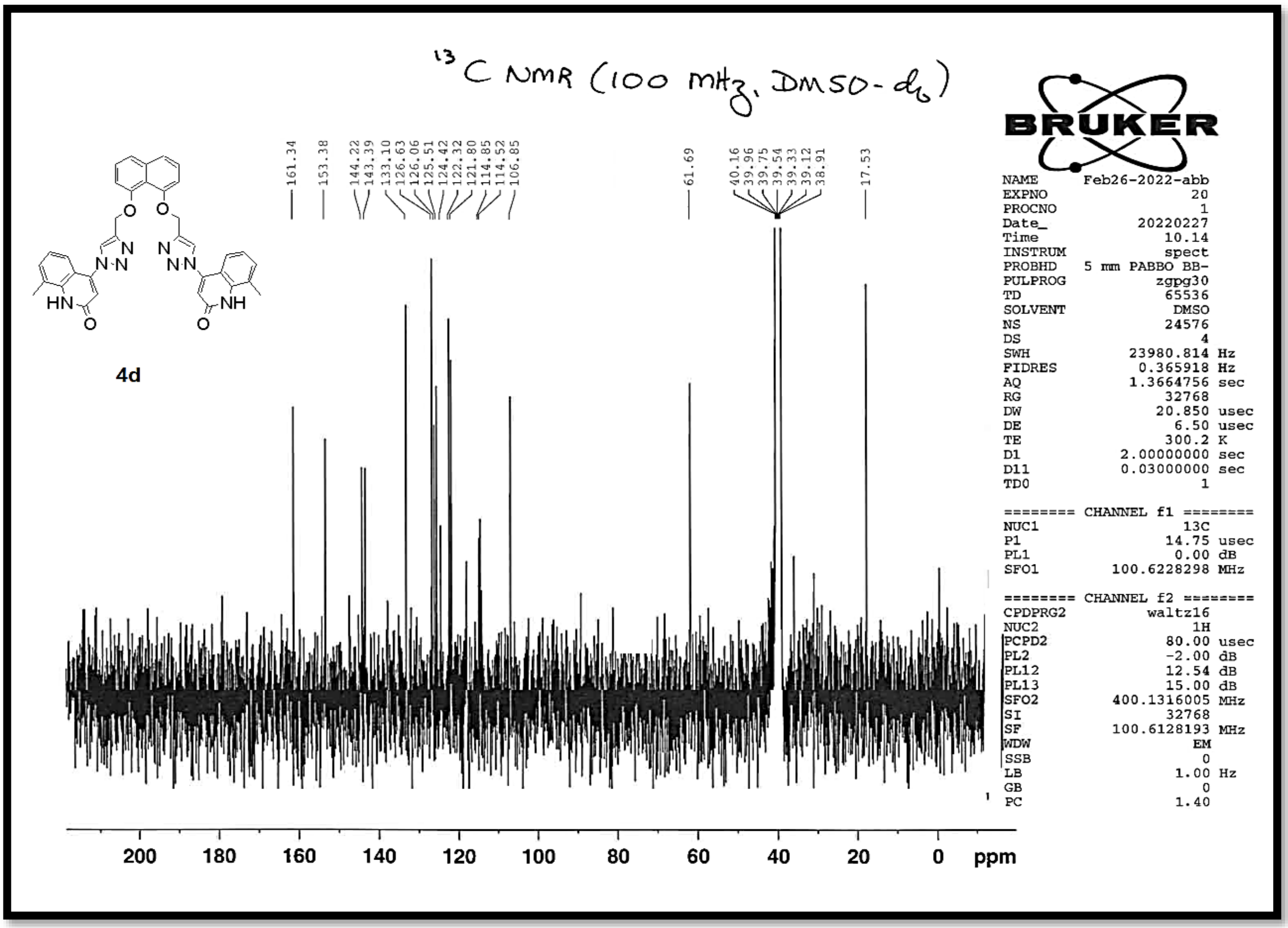

Figure S31. ^{13}C NMR spectrums for compound **4d.**

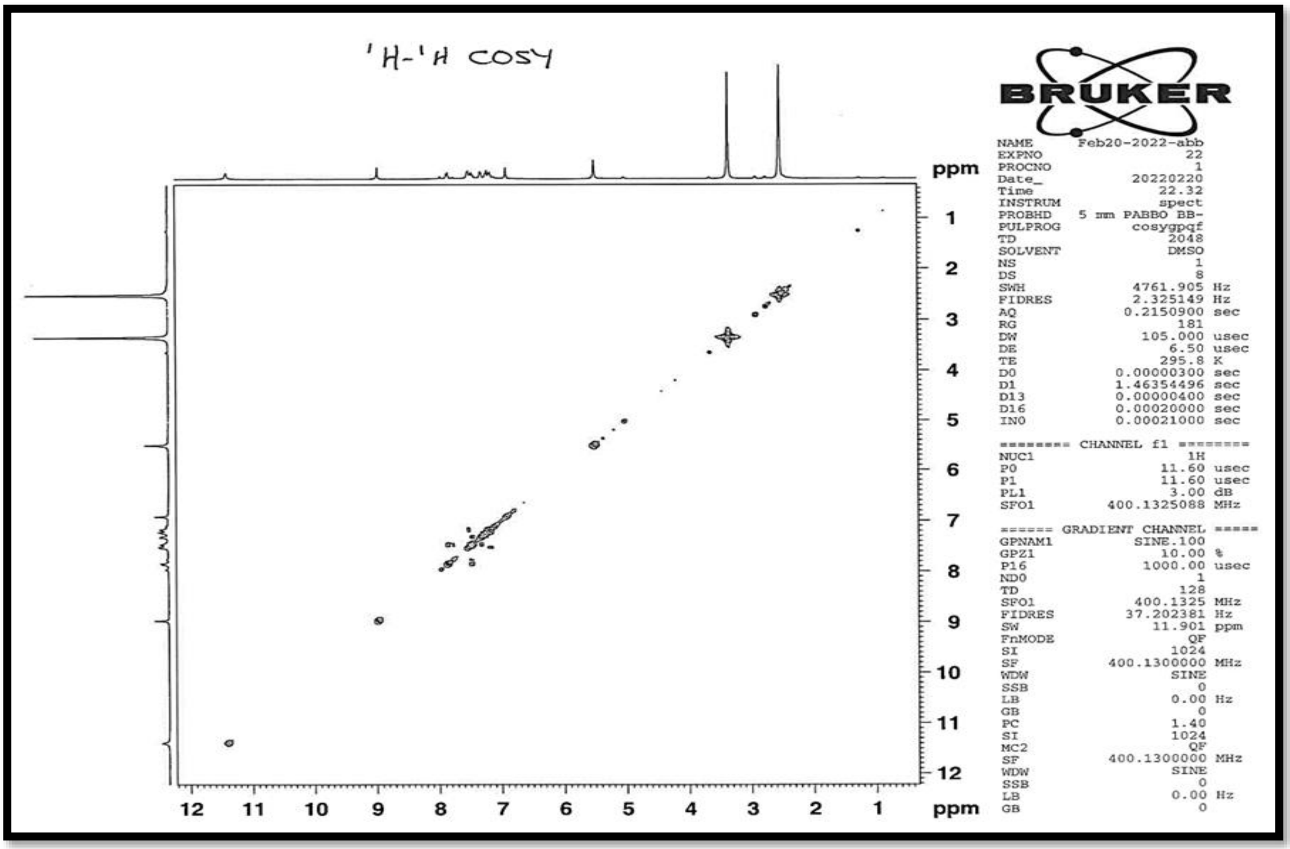

Figure S32. ^{1}H-^{1}H Cosy spectrums for compound **4d.**

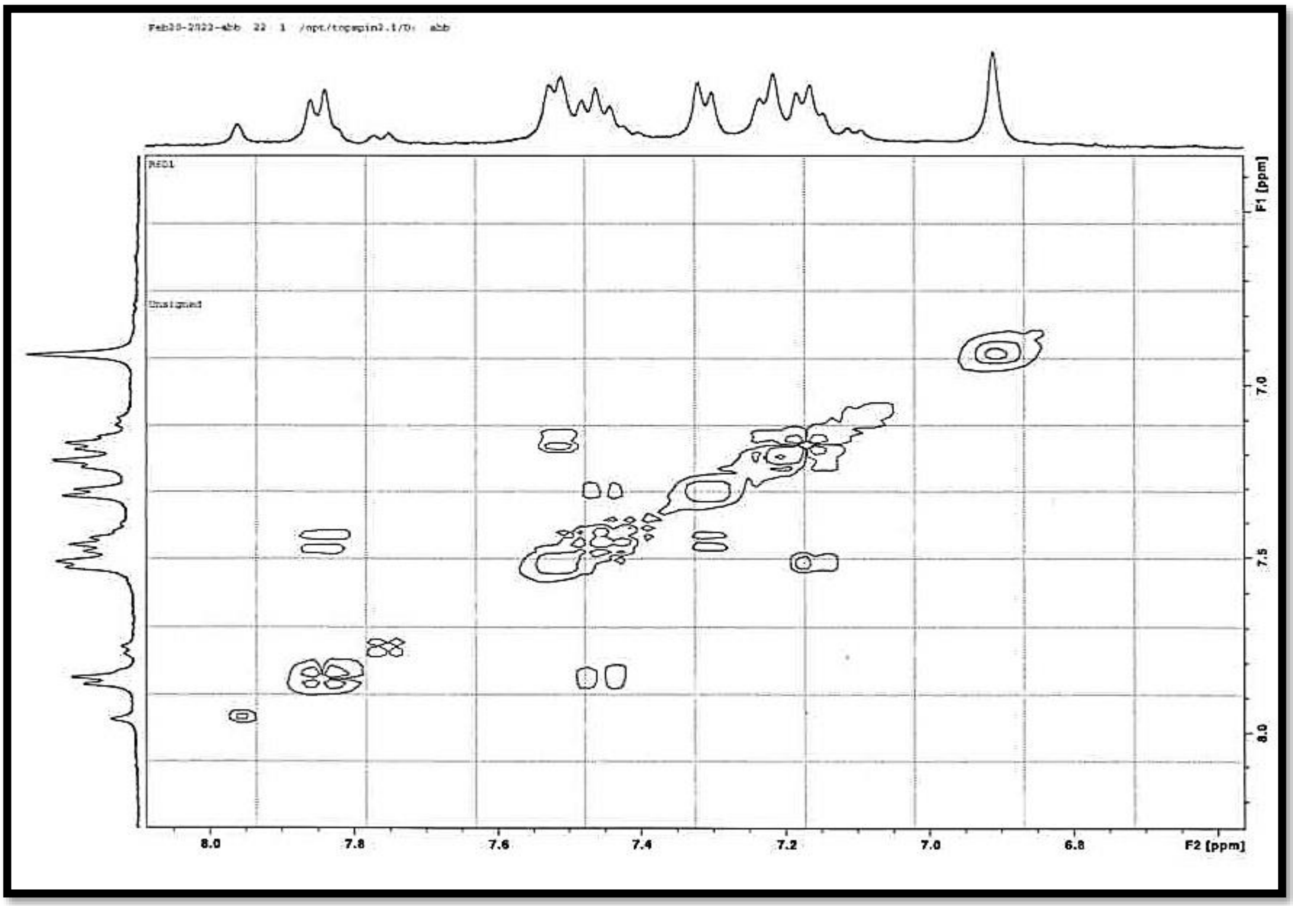

Figure S33. Part of the ^{1}H-^{1}H Cosy spectrums for compound **4d.**

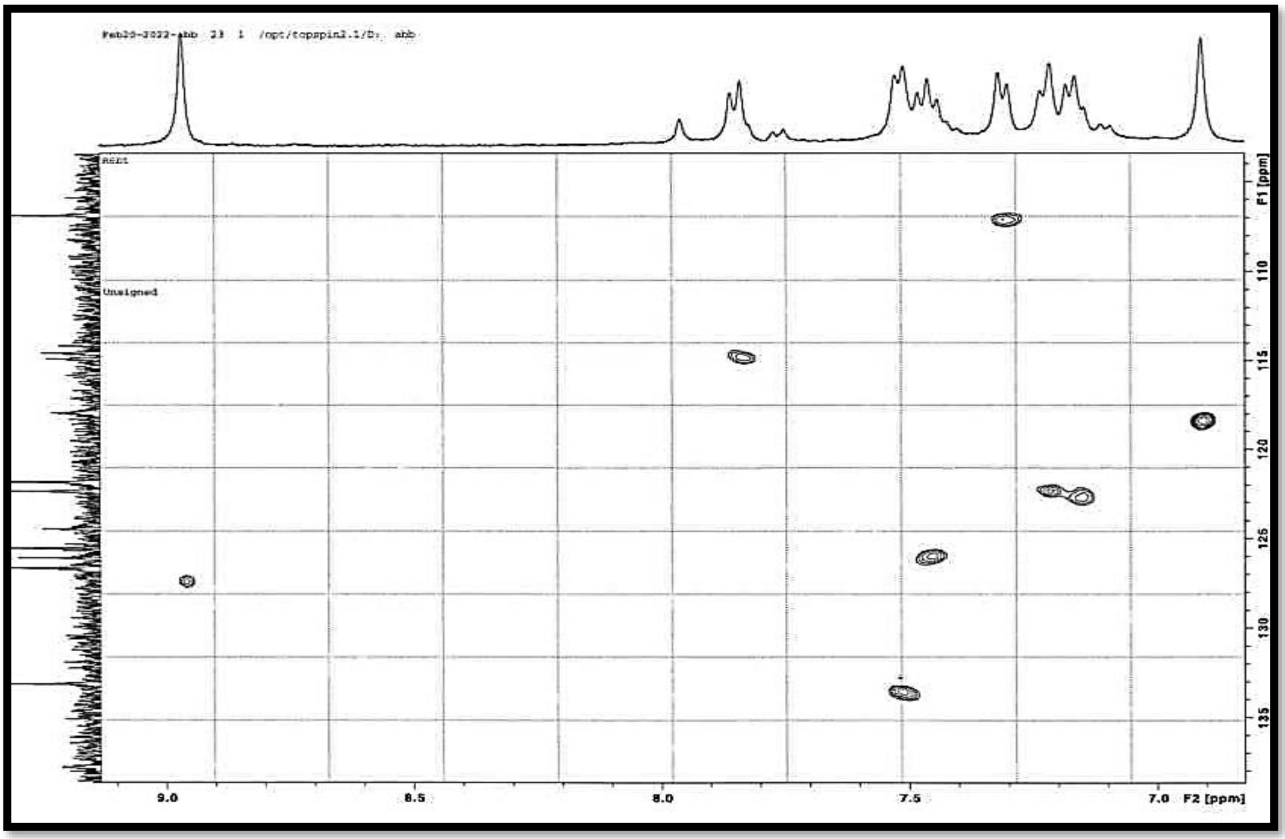

Figure S34. Part of the ^{1}H-^{13}C HSQC spectrums for compound **4d.**

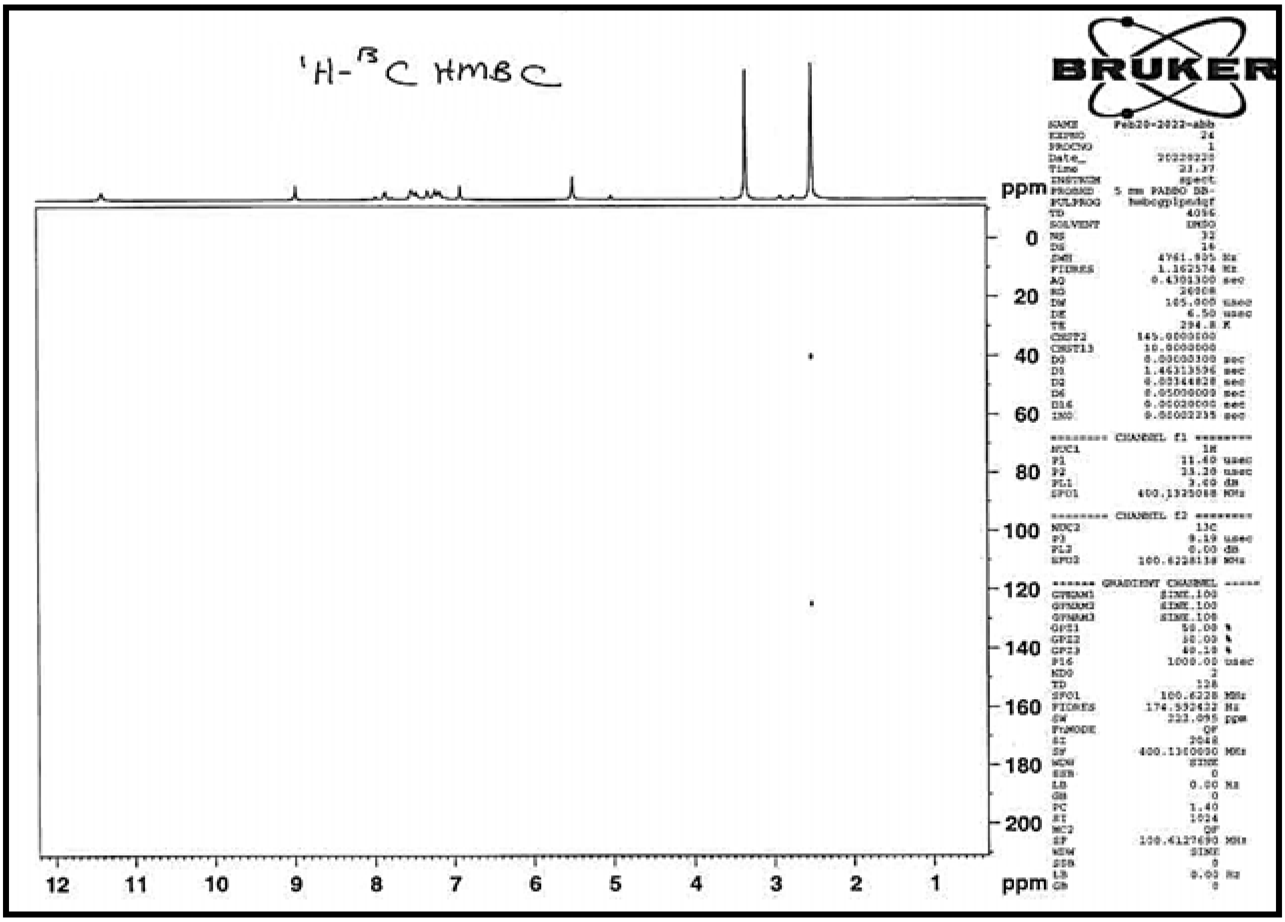

Figure S35. ^{1}H-^{13}C HMBC spectrums for compound **4d.**

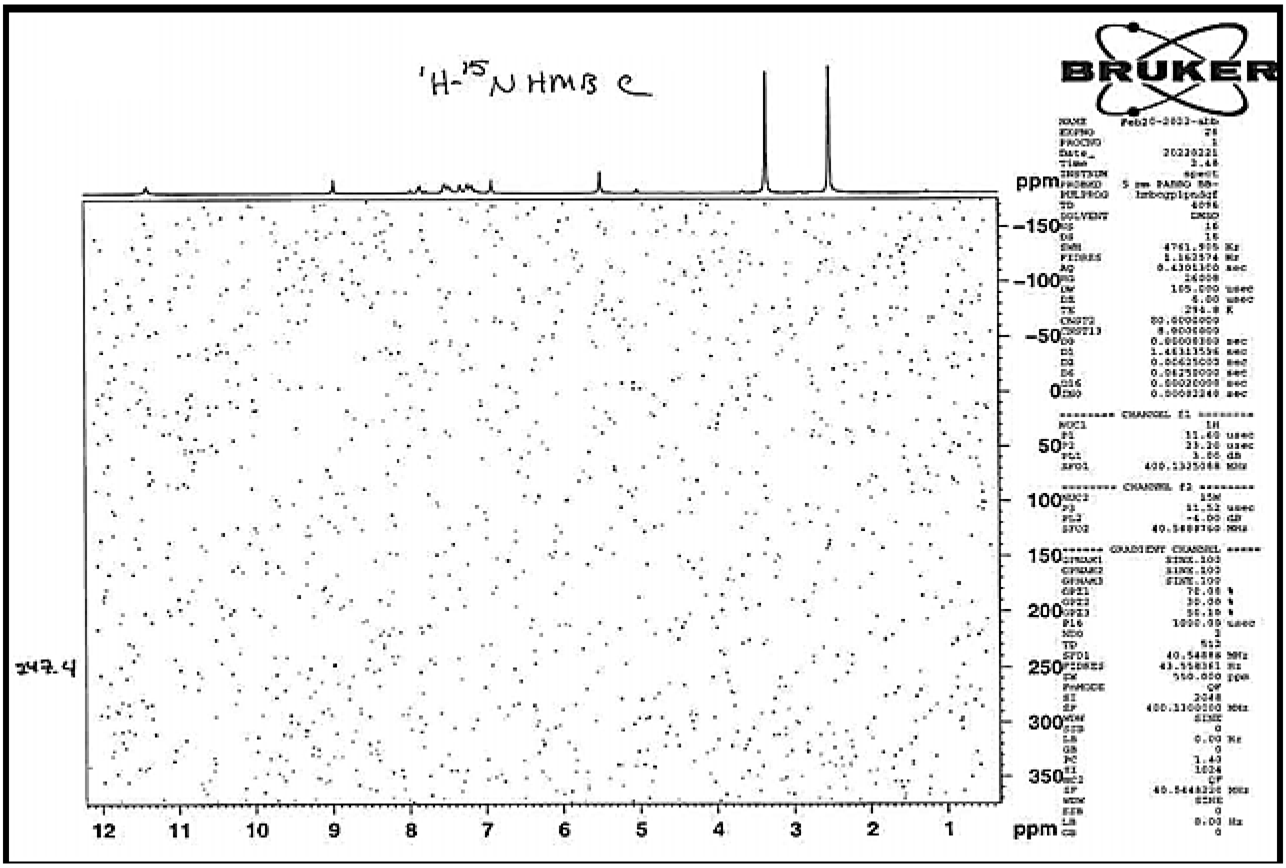

Figure S36. ^{1}H-^{15}N HMBC spectrums for compound **4d.**

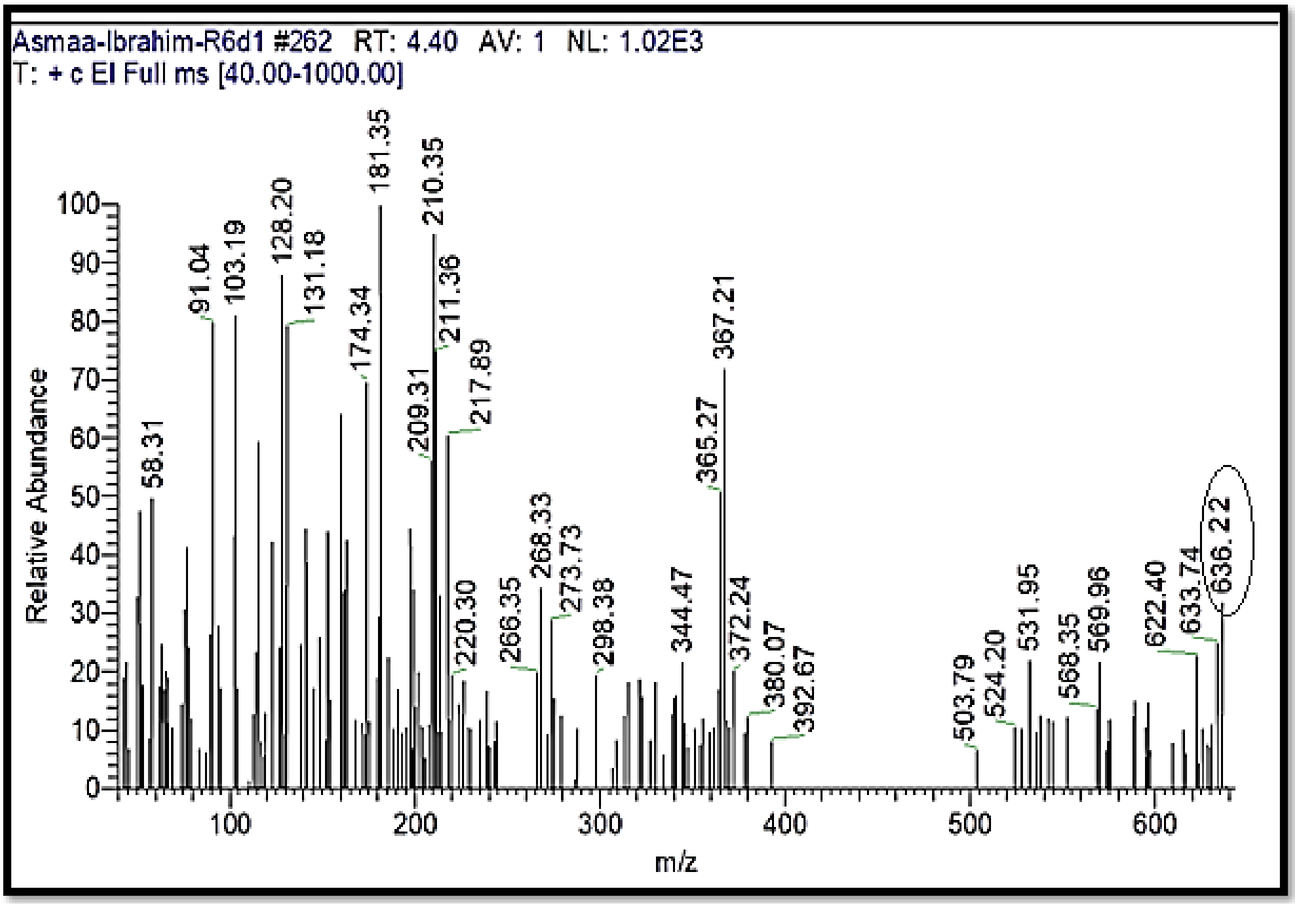

Figure S37. Mass spectrometry for compound **4d.**

Spectral data for compound 4e.

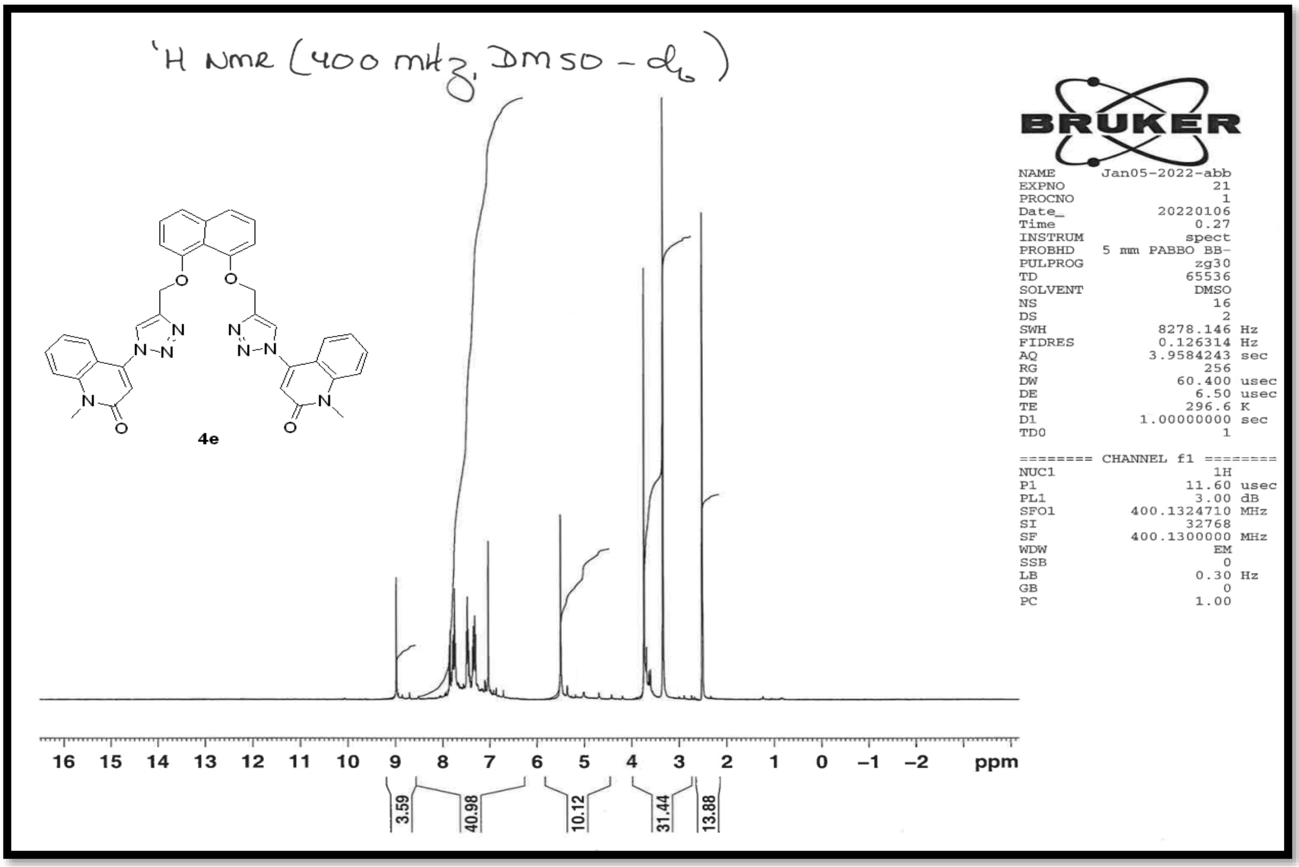

Figure S38. ^{1}H NMR spectrums for compound **4e.**

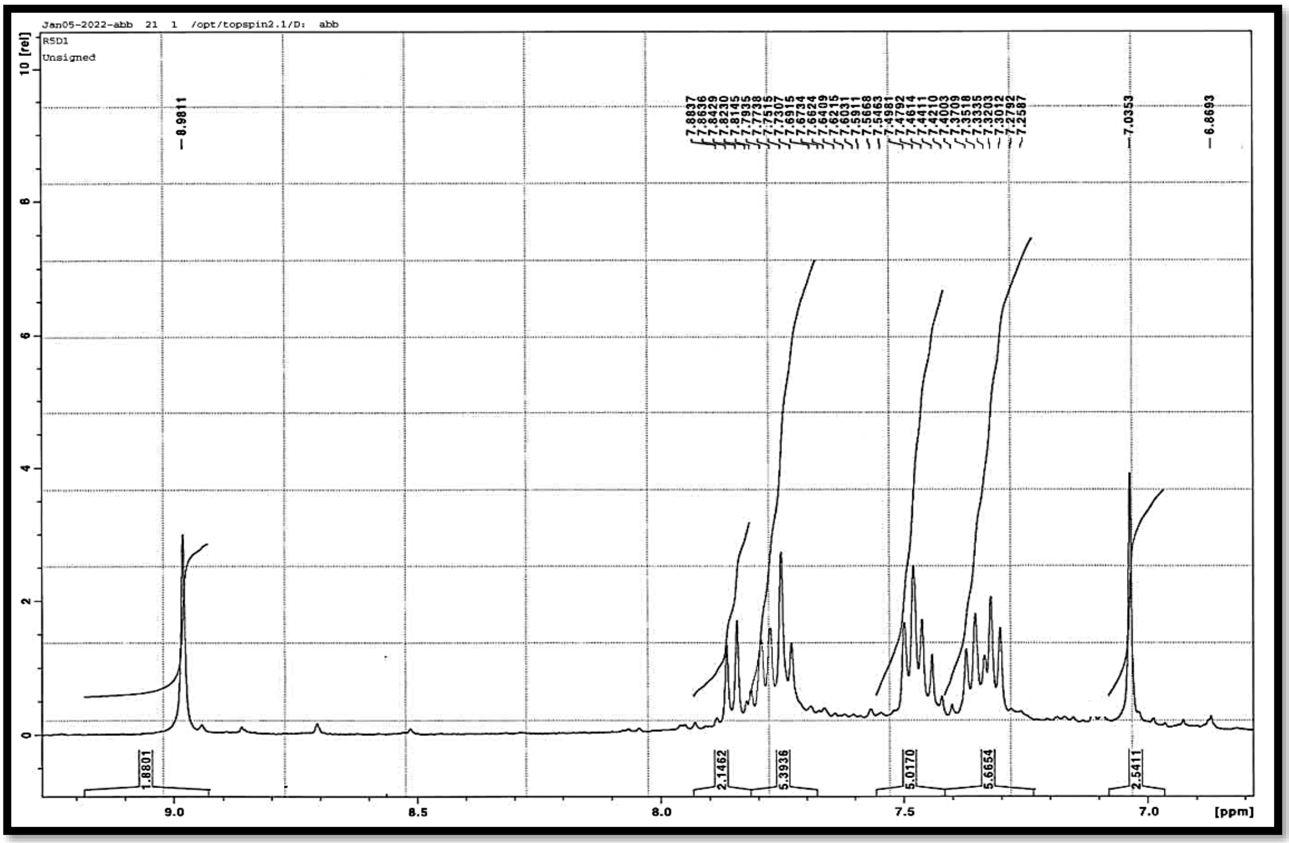

Figure S39. Part of the ^{1}H NMR spectrums for compound **4e.**

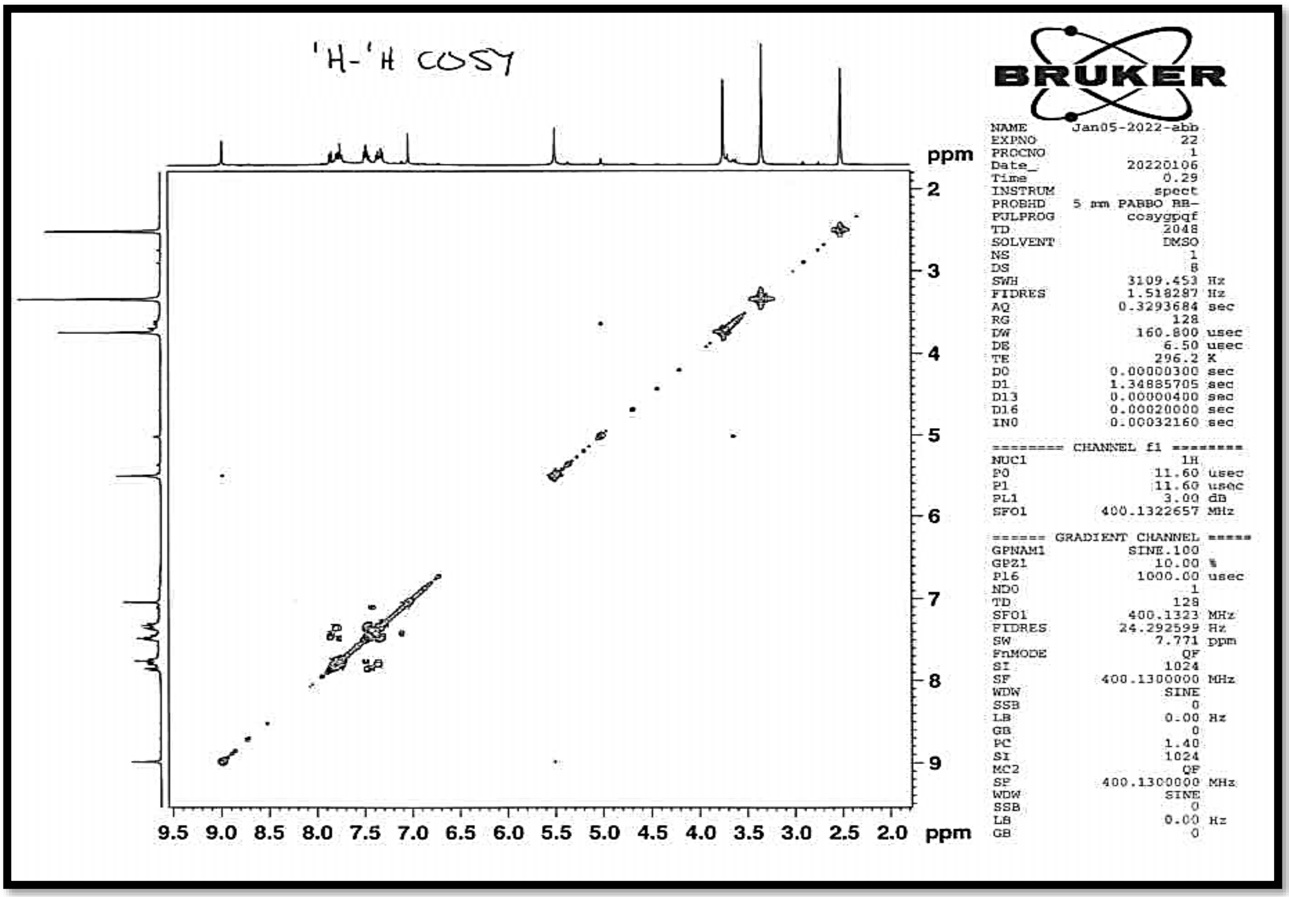

Figure S40. ^{1}H-^{1}H Cosy spectrums for compound **4e.**

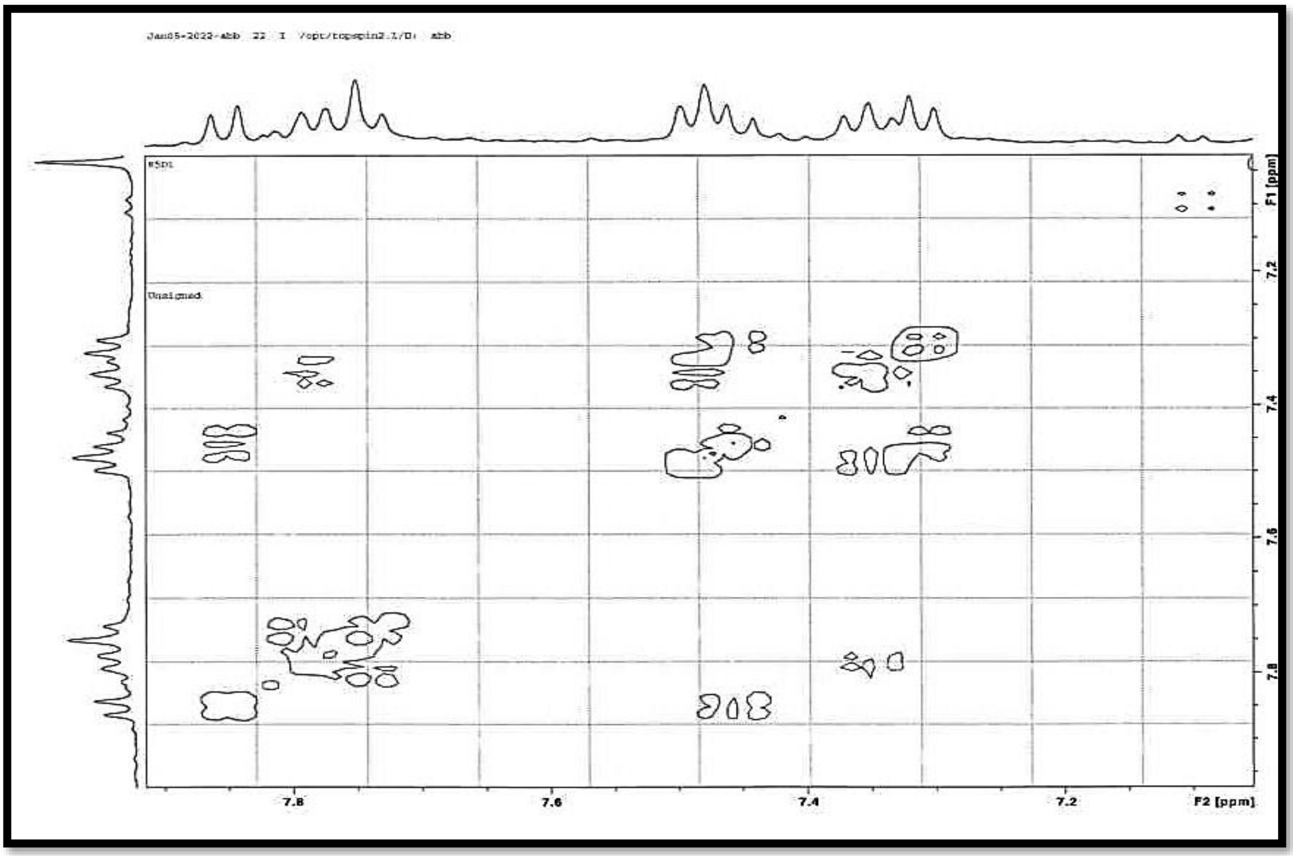

Figure S41. Part of the ^{1}H-^{1}H Cosy spectrums for compound **4e.**

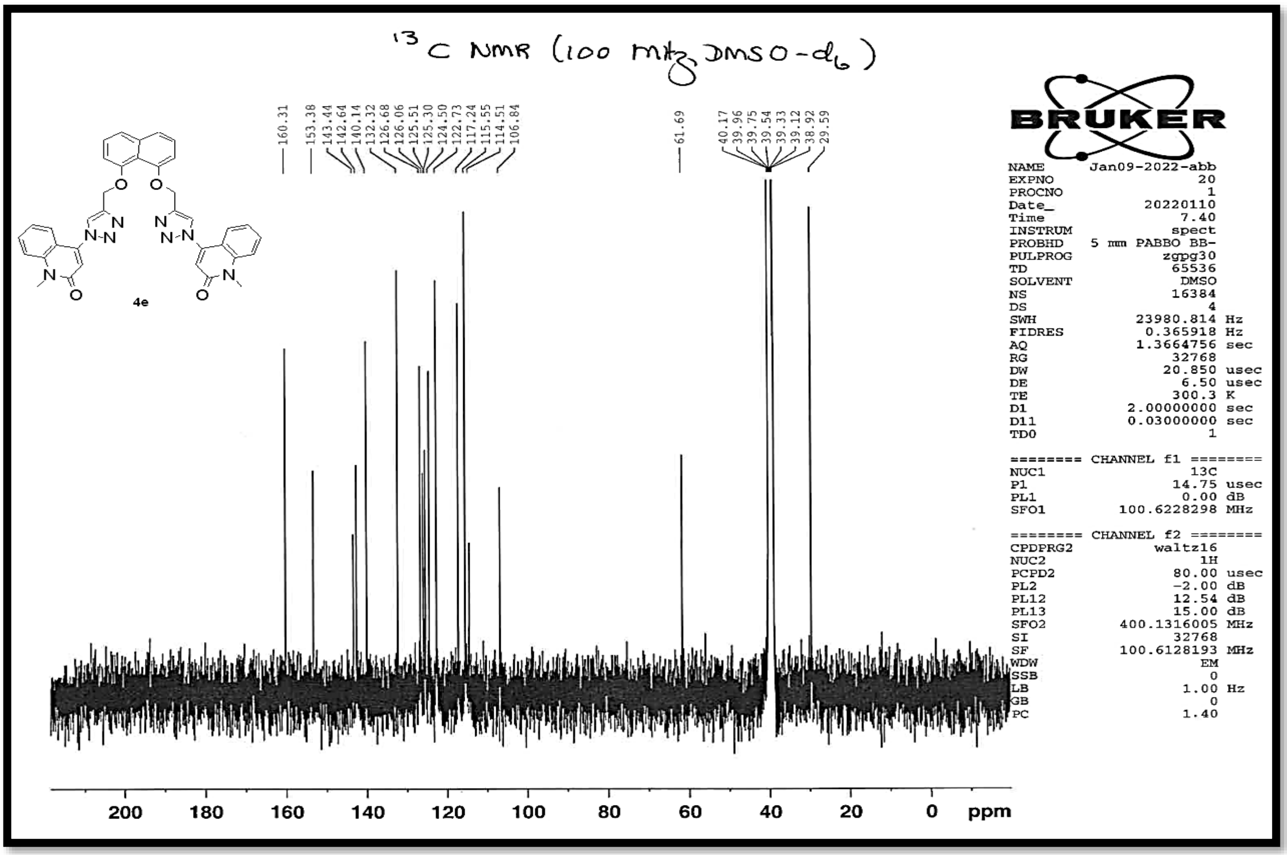

Figure S42. ^{13}C NMR spectrums for compound **4e.**

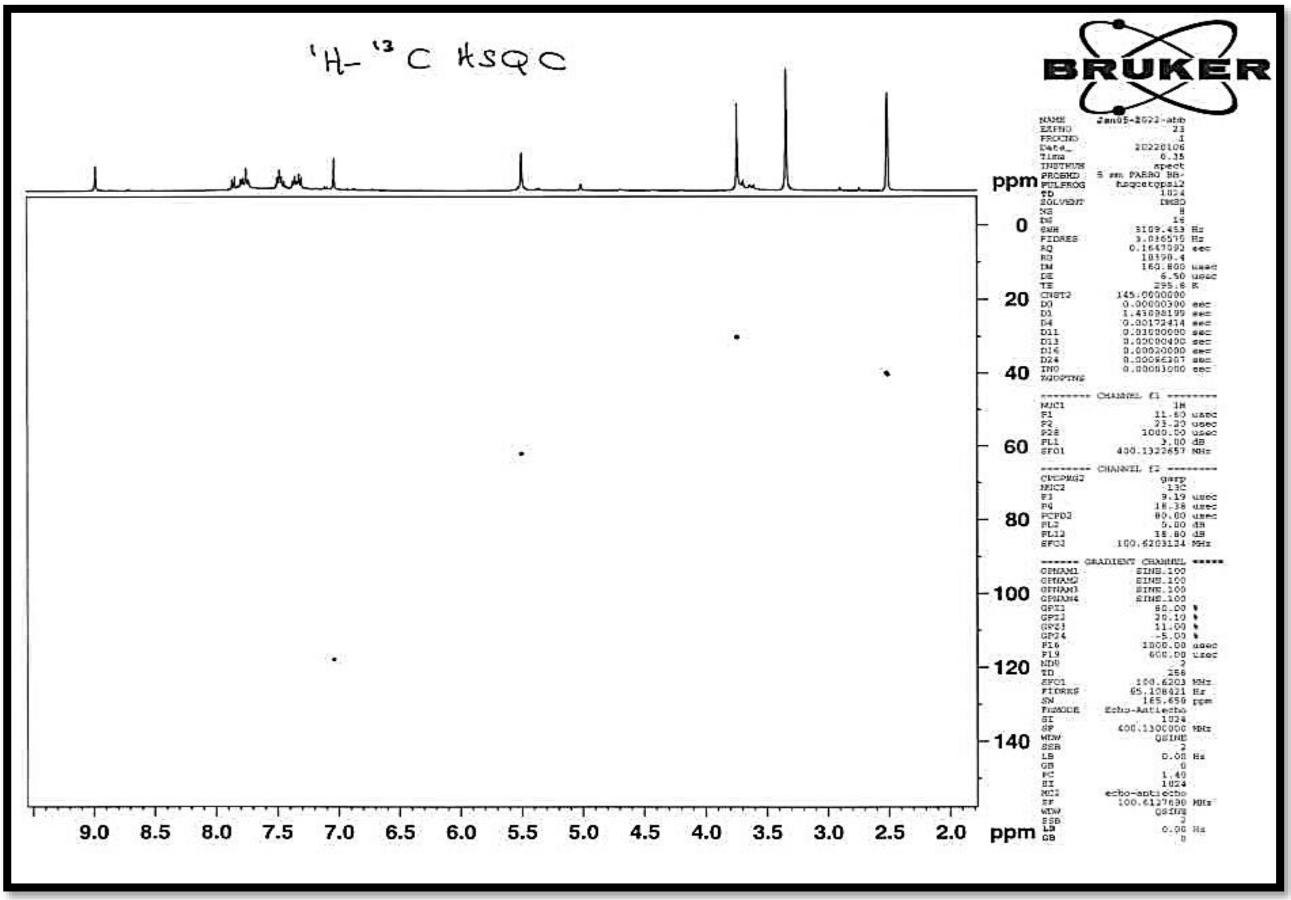

Figure S43. ^{1}H-^{13}C HSQC spectrums for compound **4e.**

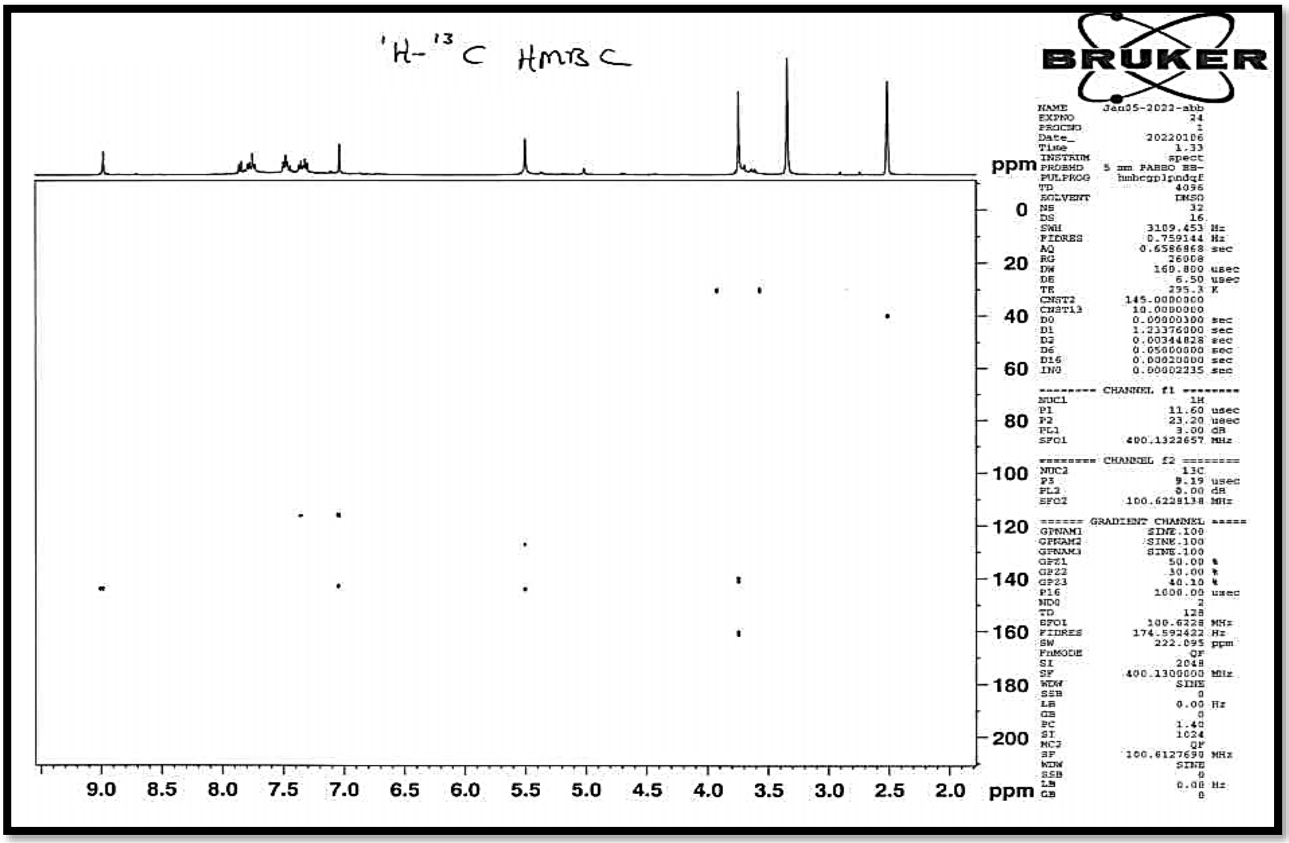

Figure S44. ^{1}H-^{13}C HMBC spectrums for compound **4e.**

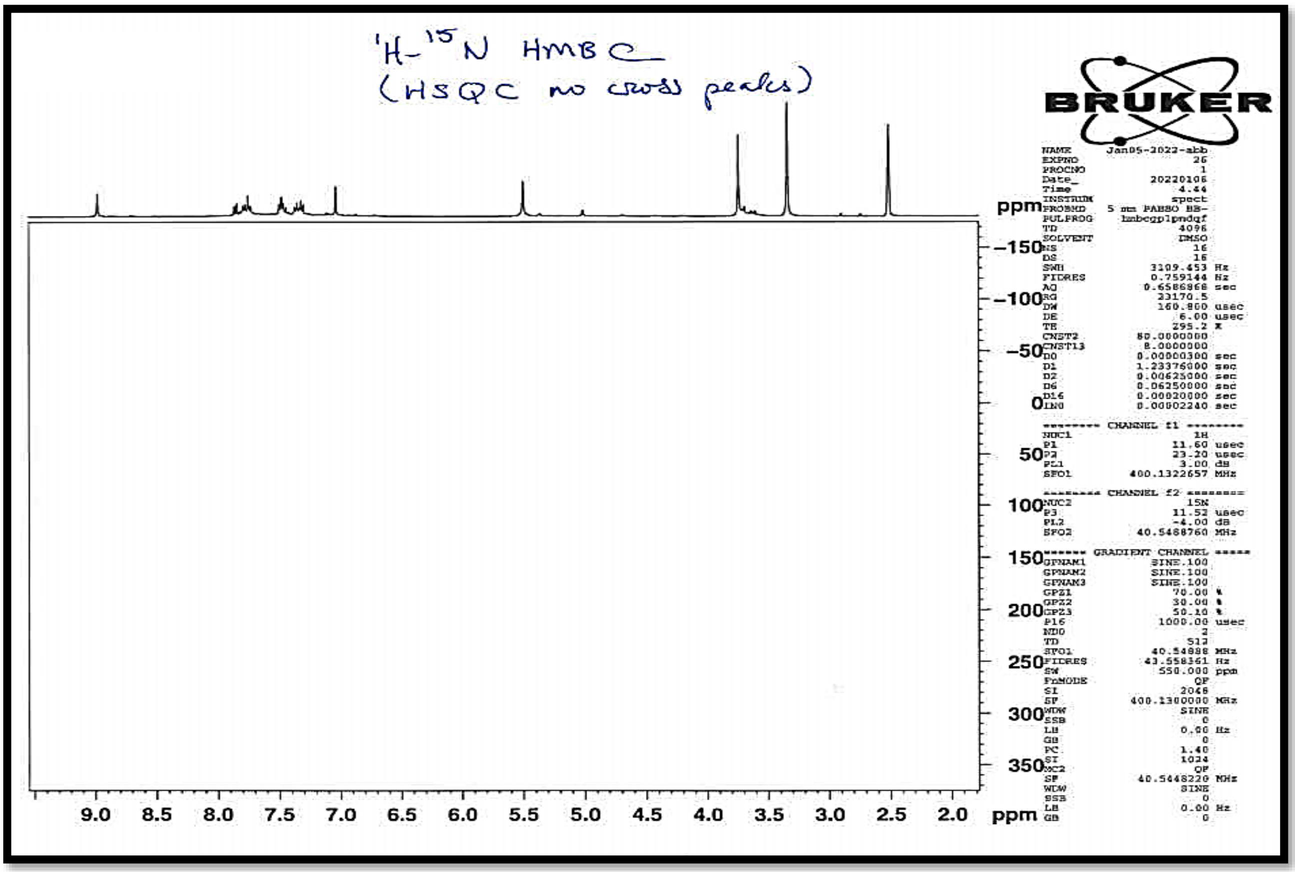

Figure S45. ^{1}H-^{15}N HMBC spectrums for compound **4e.**

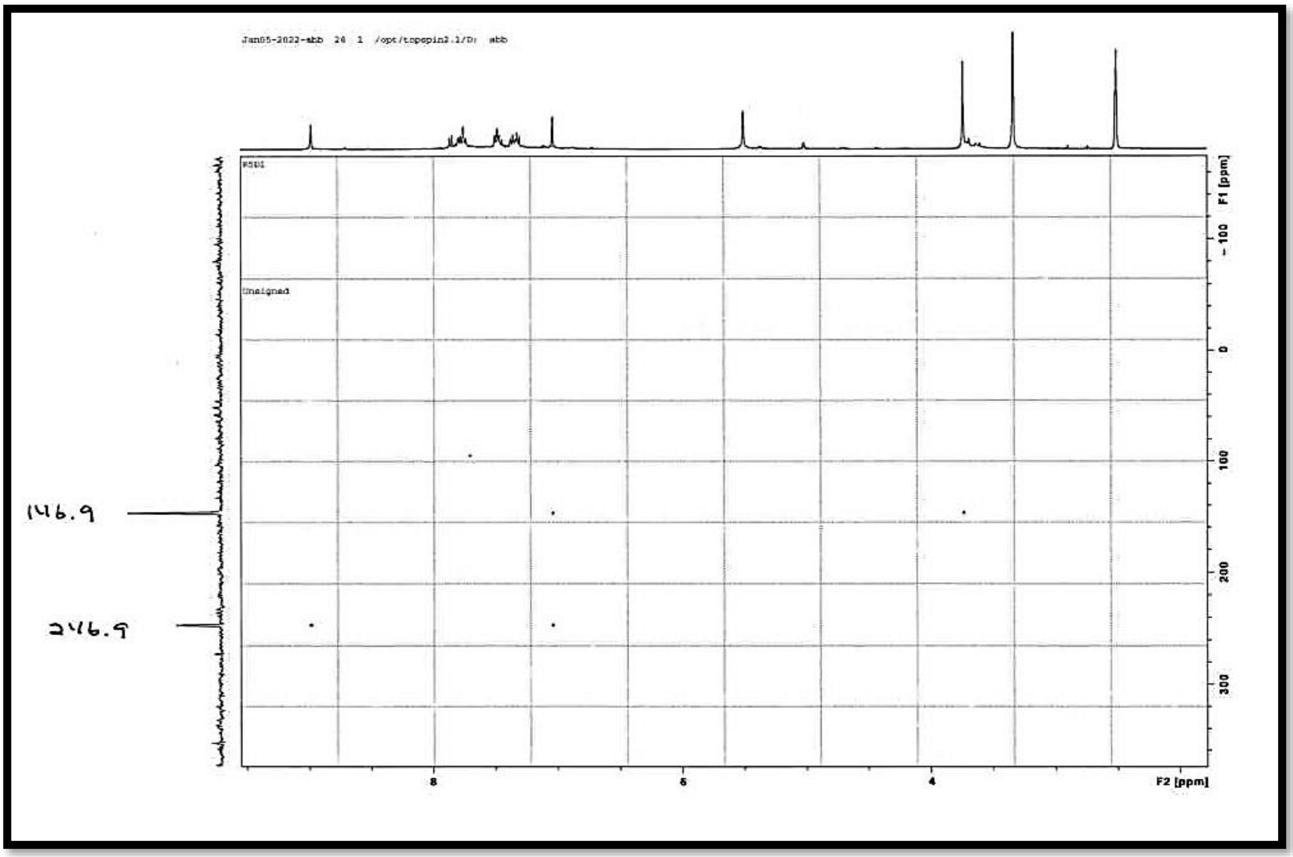

Figure S46. Part of the ^{1}H-^{15}N HMBC spectrums for compound **4e.**

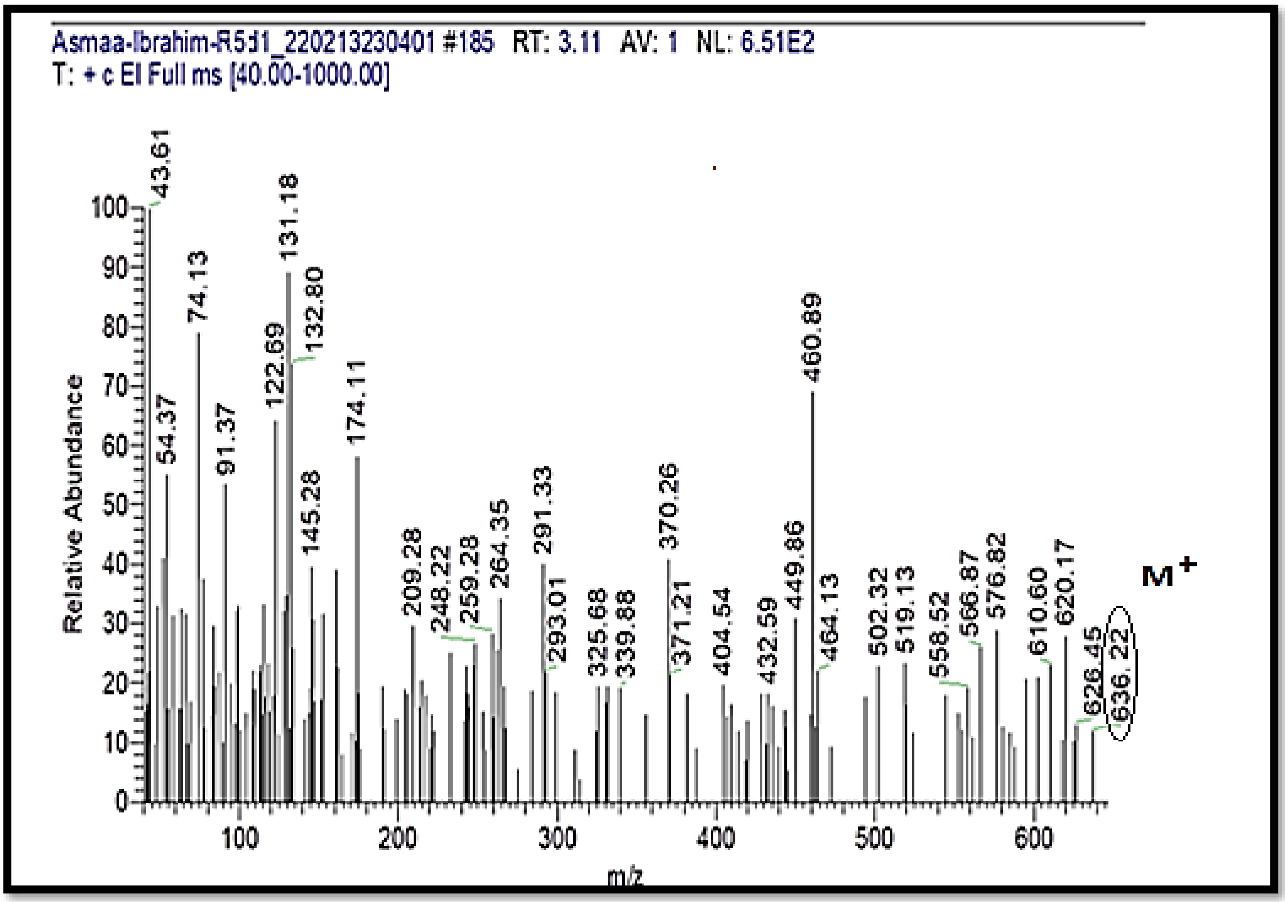

Figure S47. Mass spectrometry for compound **4e.**

Spectral Data for compound 6.

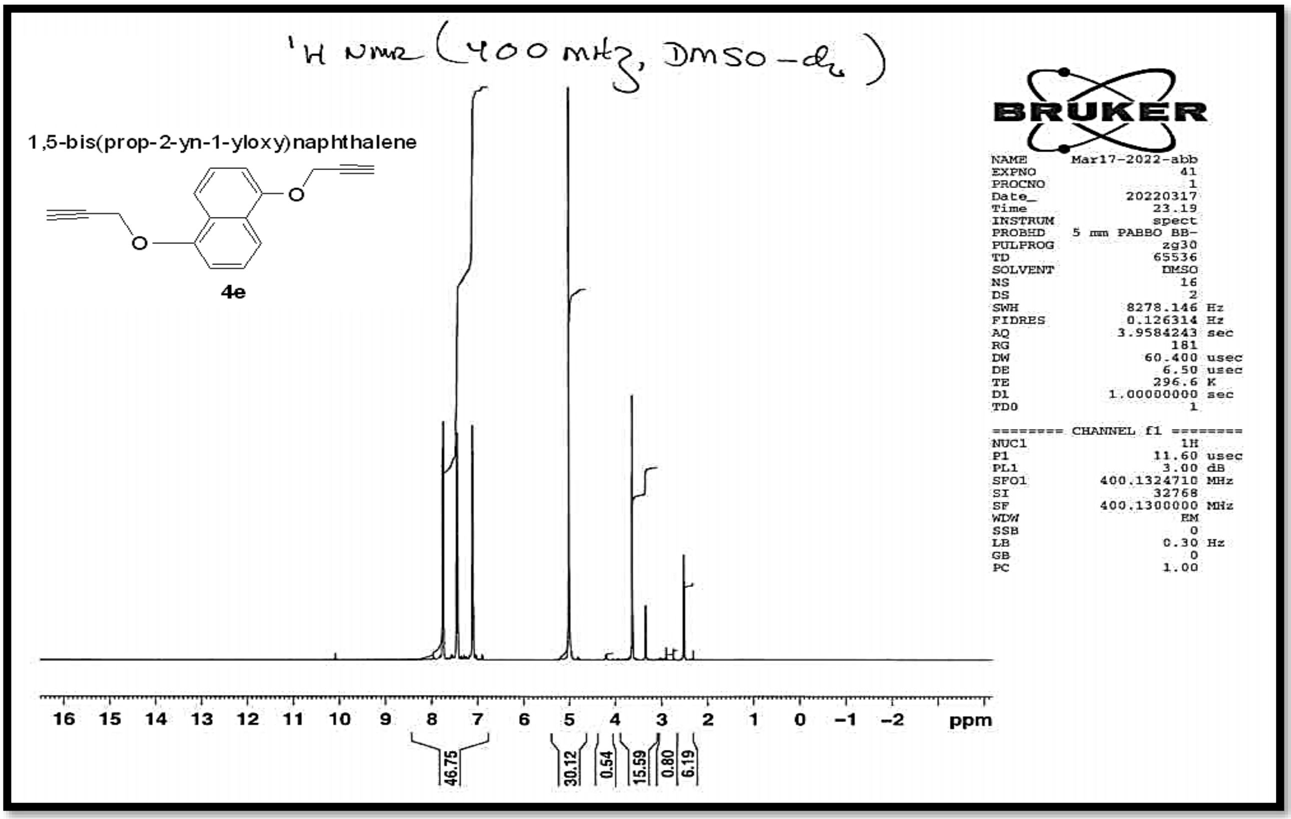

Figure S48. ^{1}H NMR spectrums for compound **6.**

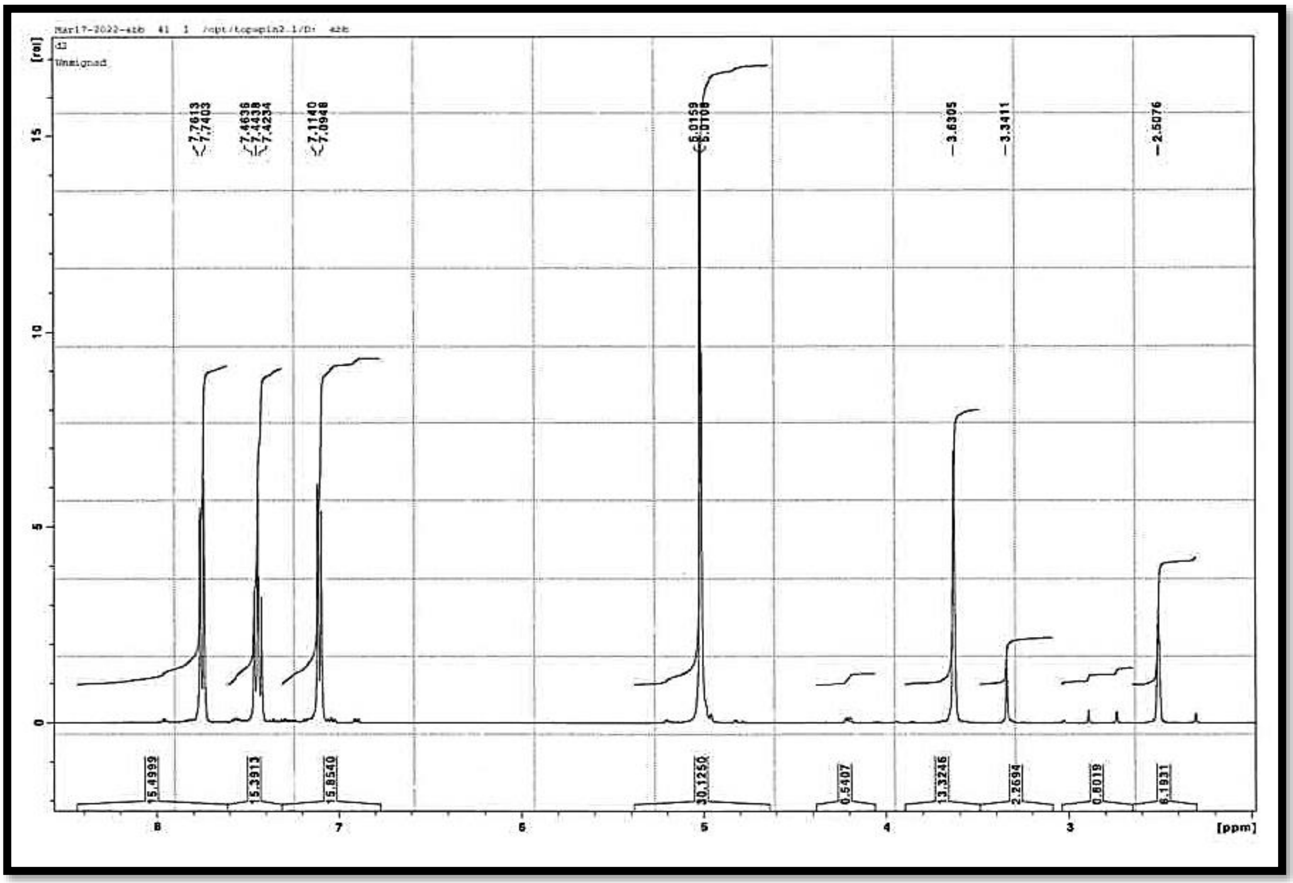

Figure S49. Part of the ^{1}H NMR spectrums for compound **6.**

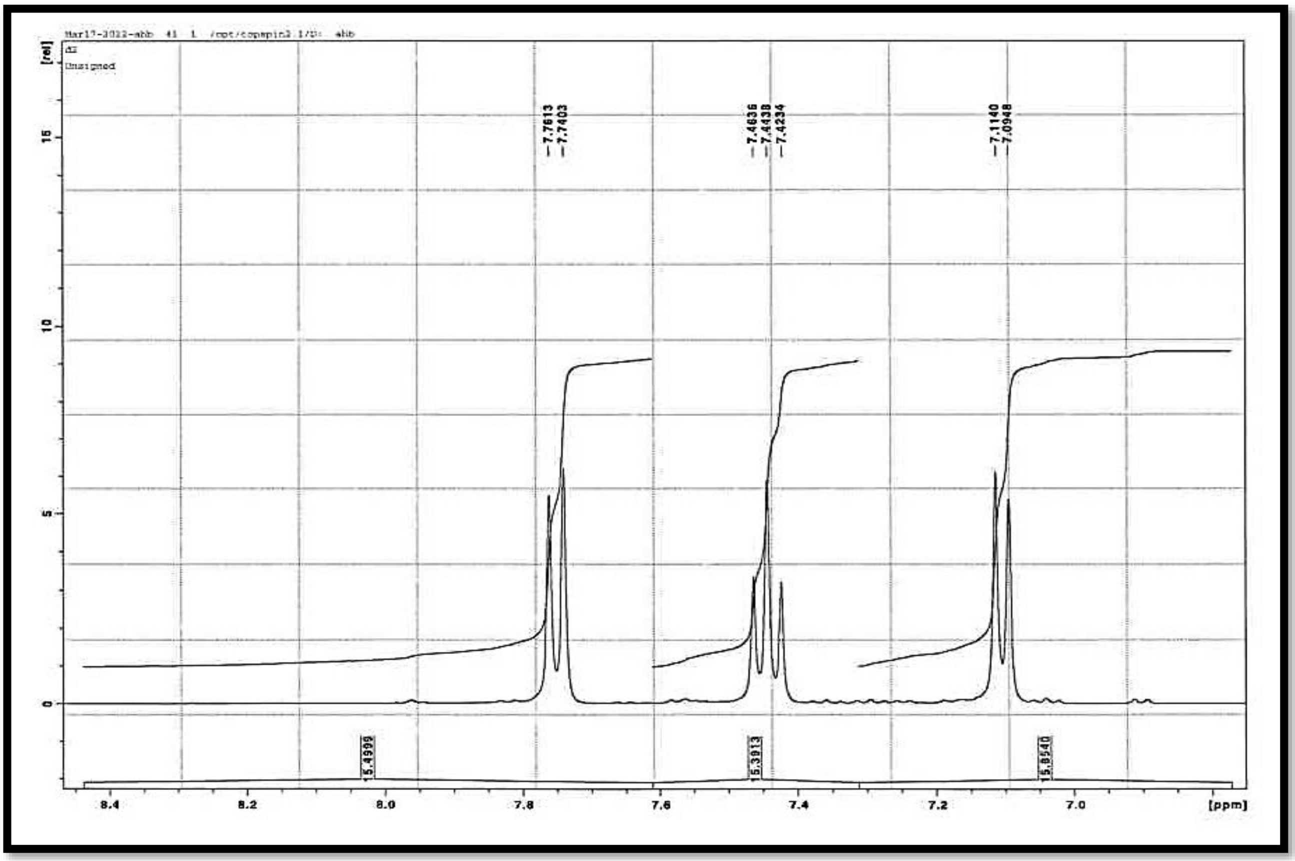

Figure S50. Part of the ^{1}H NMR spectrums for compound **6.**

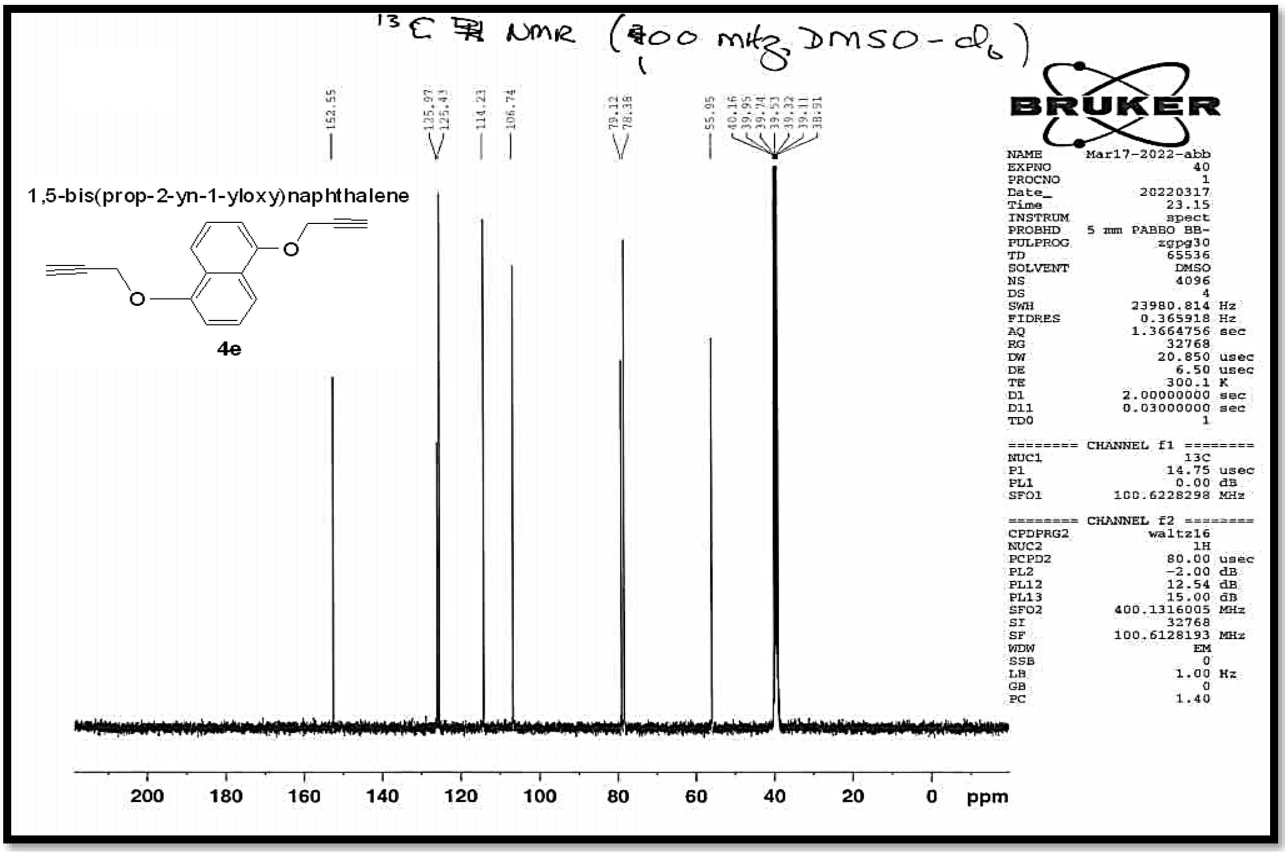

Figure S51. ^{13}C NMR spectrums for compound **6.**

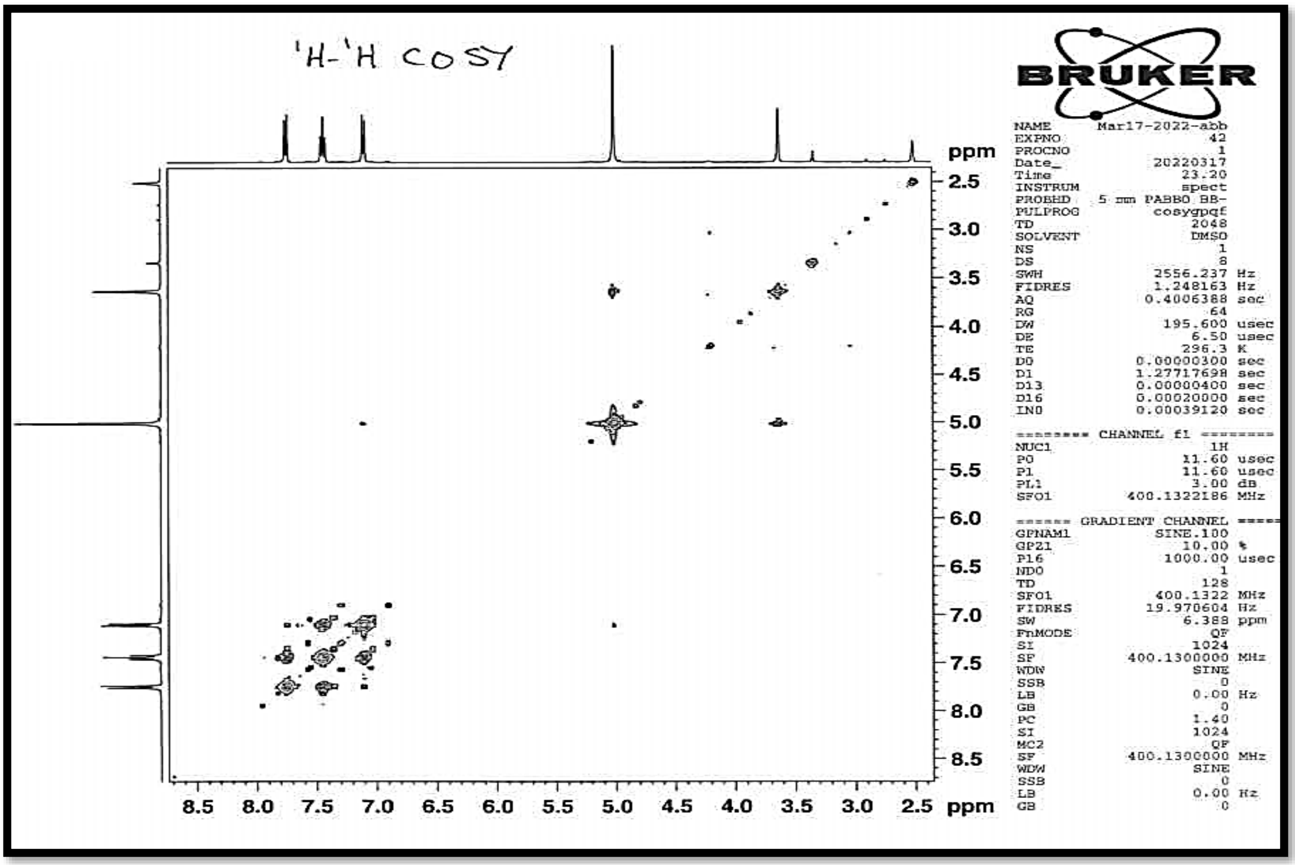

Figure S52. ^{1}H-^{1}H Cosy spectrums for compound **6.**

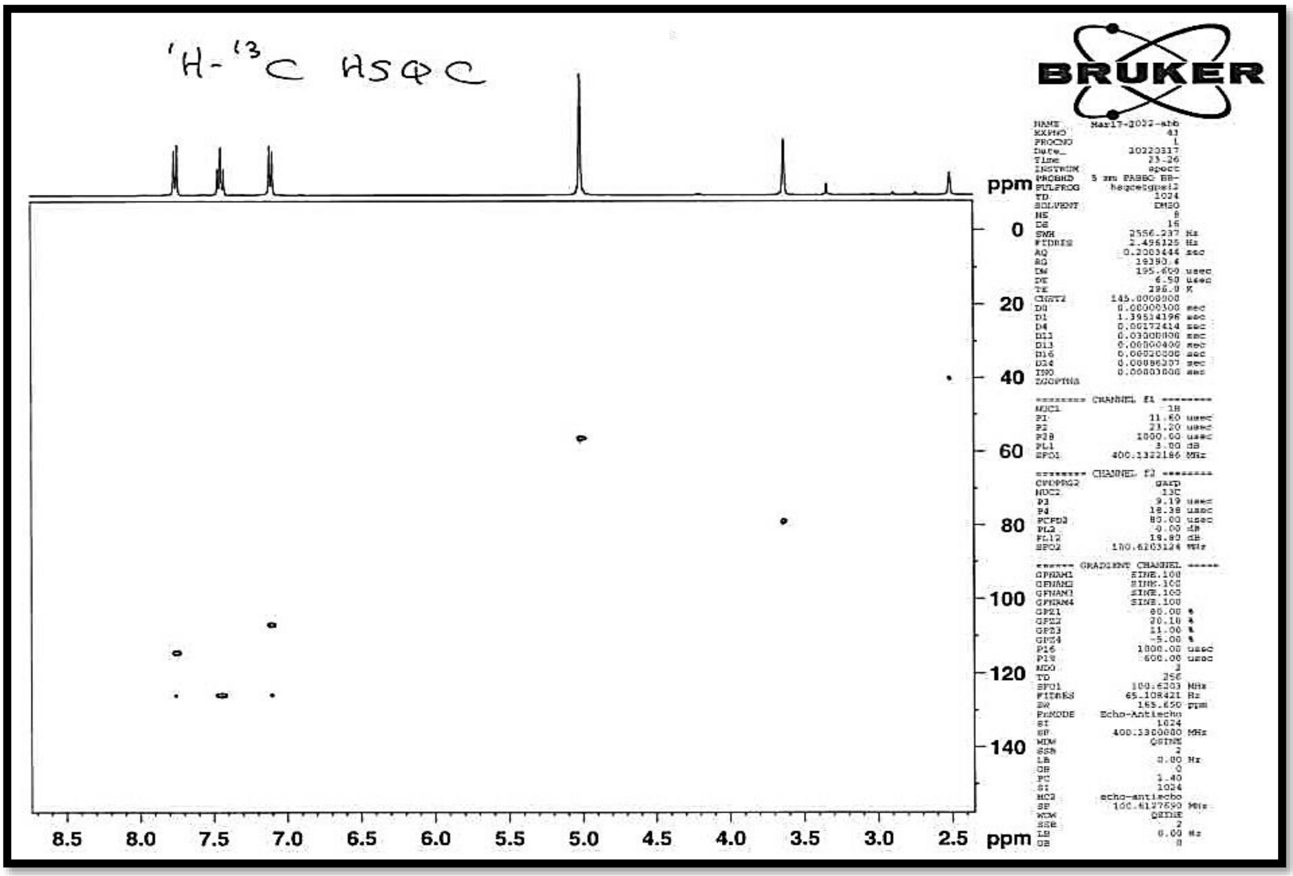

Figure S53. ^{1}H-^{13}C HSQC spectrums for compound **6.**

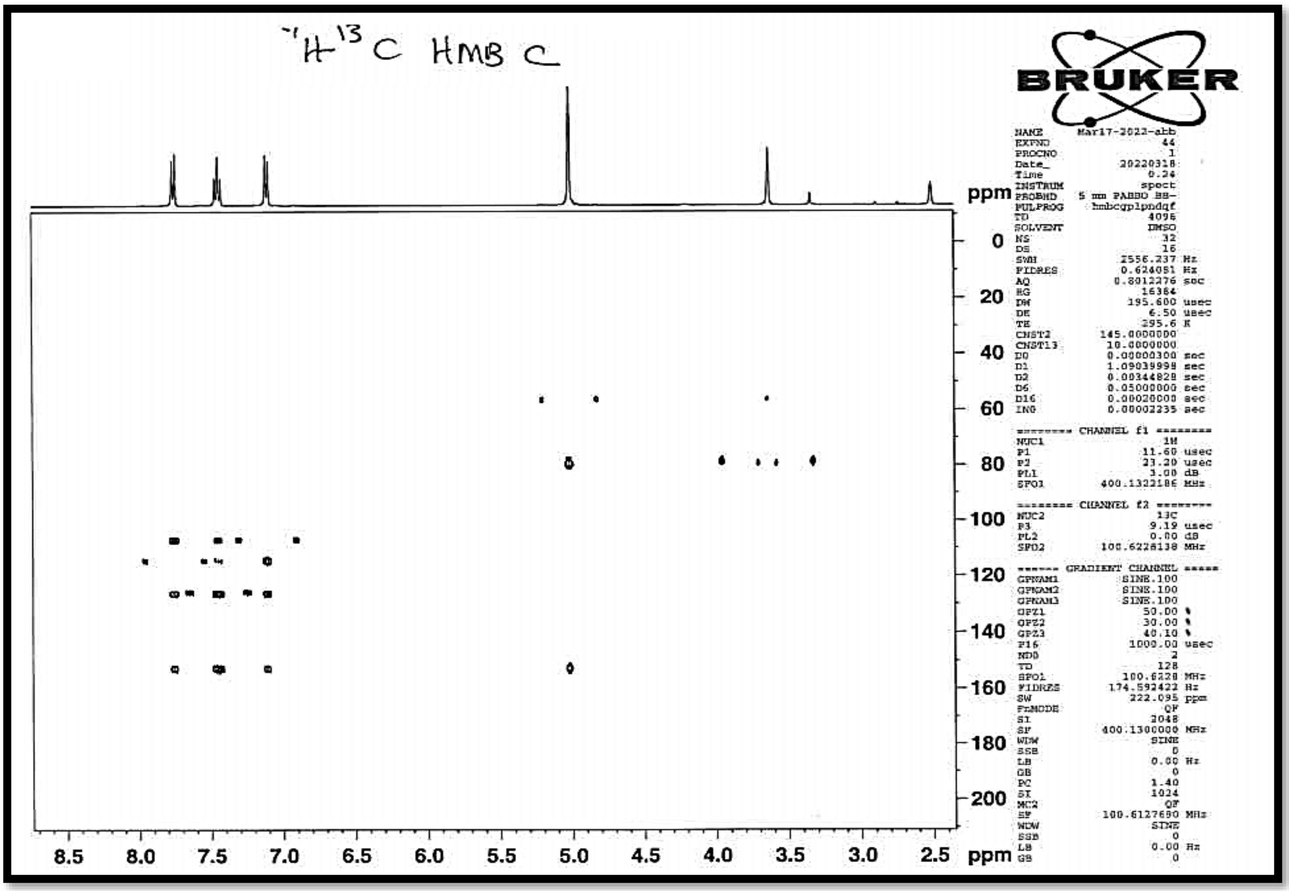

Figure S54. ^{1}H-^{13}C HMBC spectrums for compound **6.**

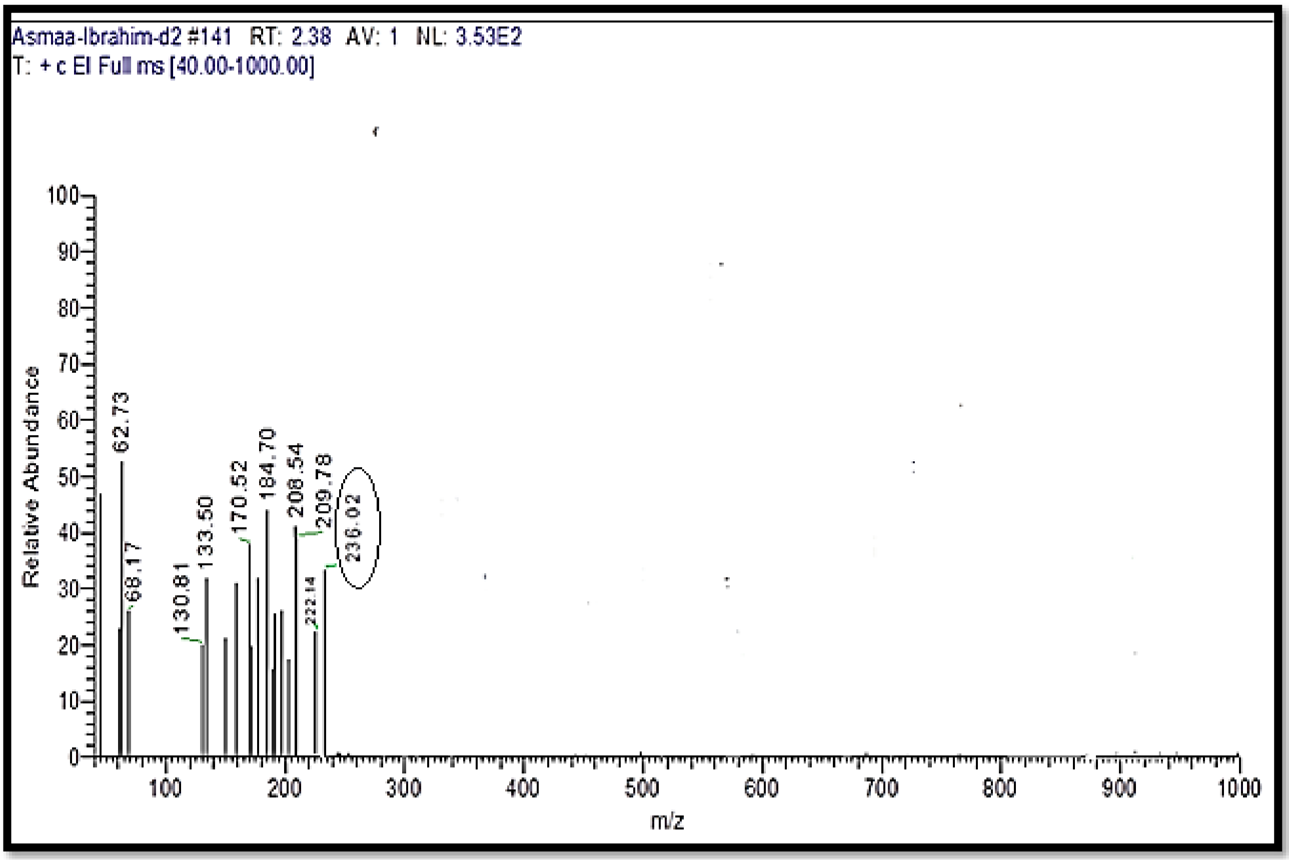

Figure S55. Mass spectrometry for compound **6.**

Spectral data for compound 7a.

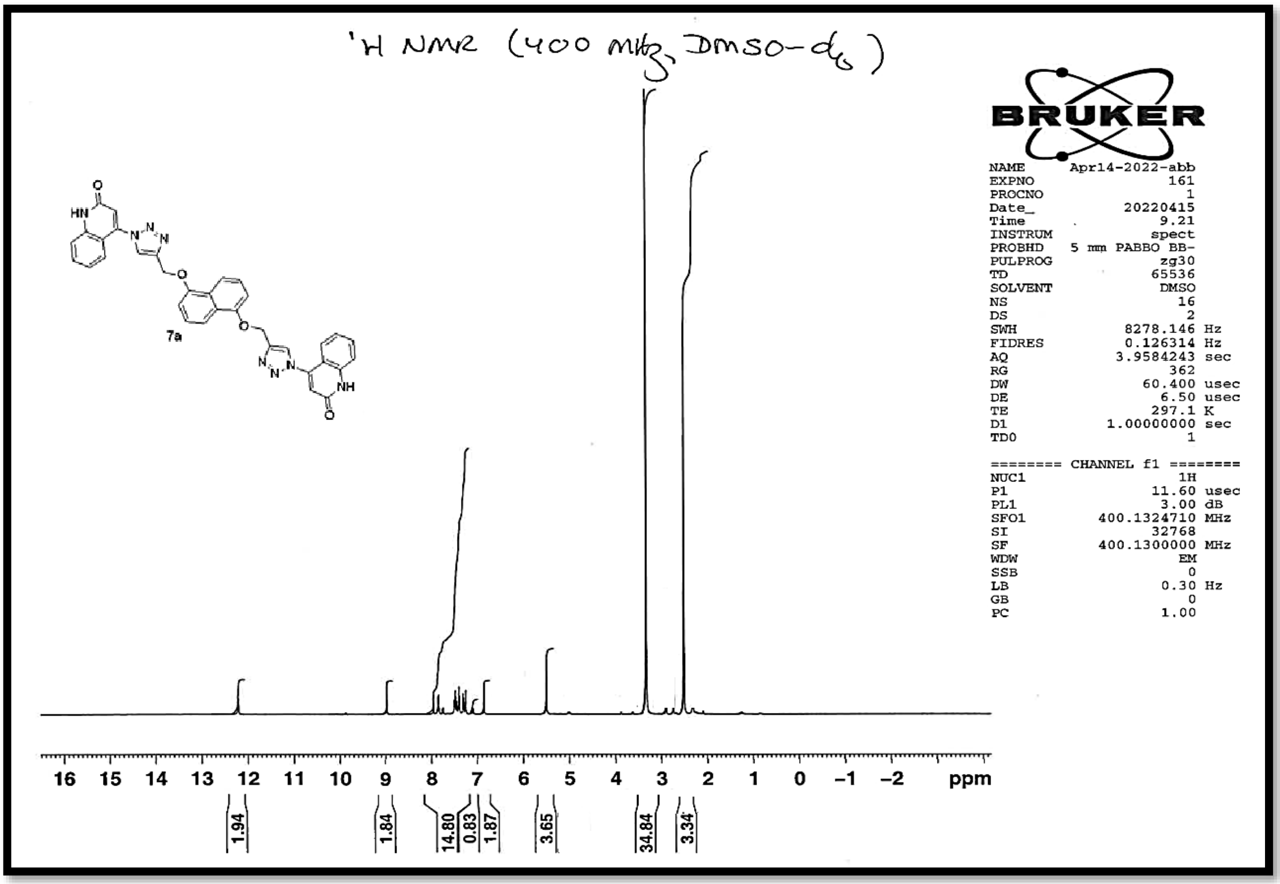

Figure S56. ^{1}H NMR spectrums for compound **7a.**

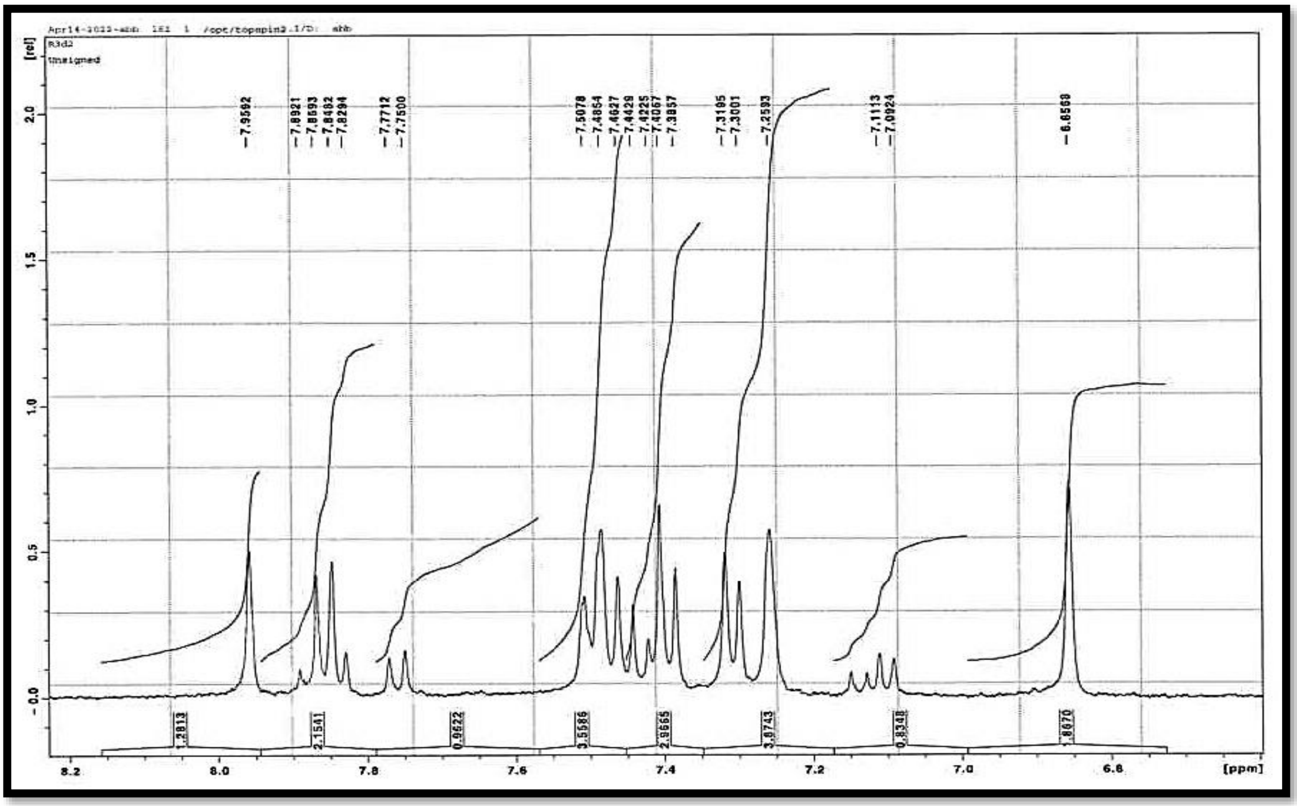

Figure S57. Part of the ^{1}H NMR spectrums for compound **7a.**

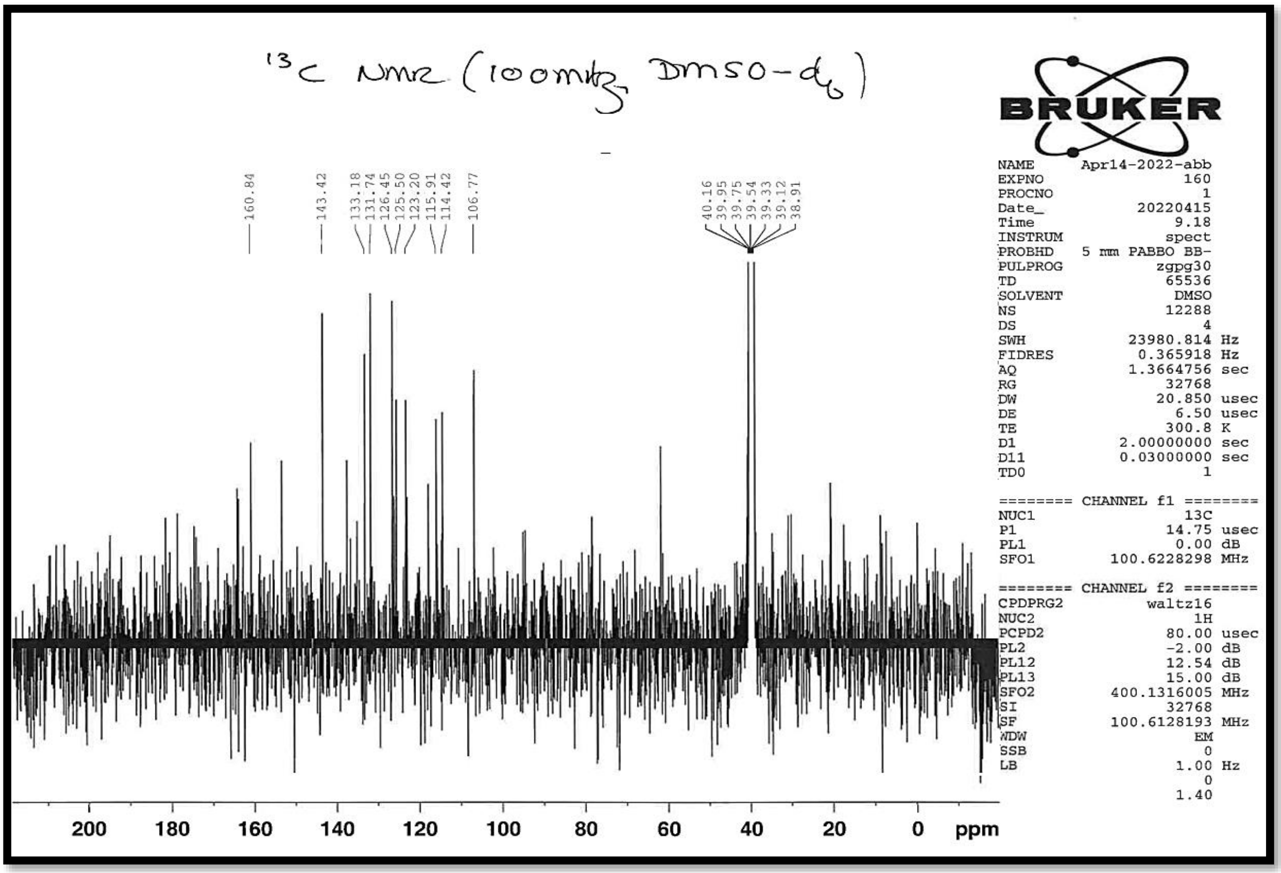

Figure S58. ^{13}C NMR spectrums for compound **7a.**

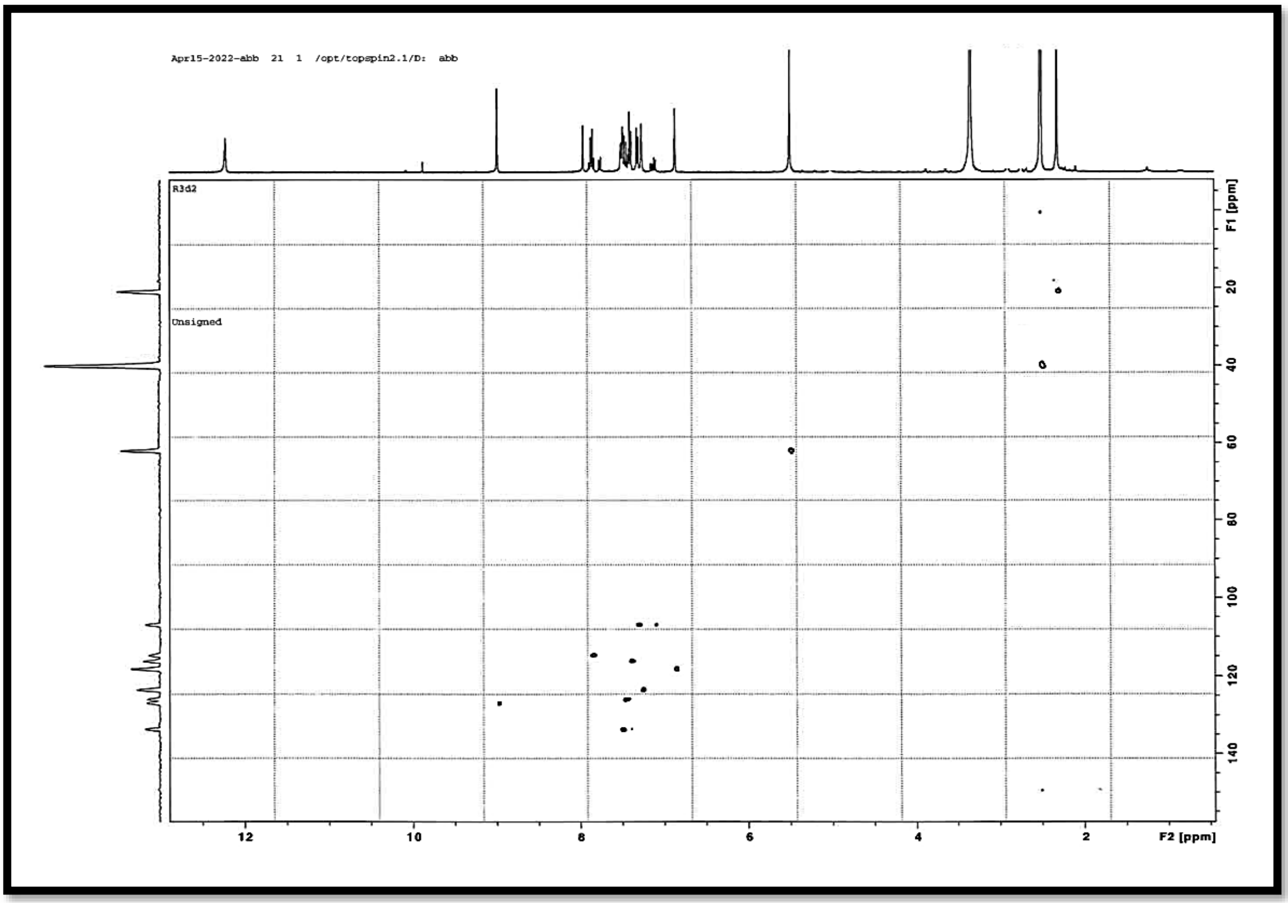

Figure S59. Part of ^{1}H-^{13}C HSQC spectrums for compound **7a.**

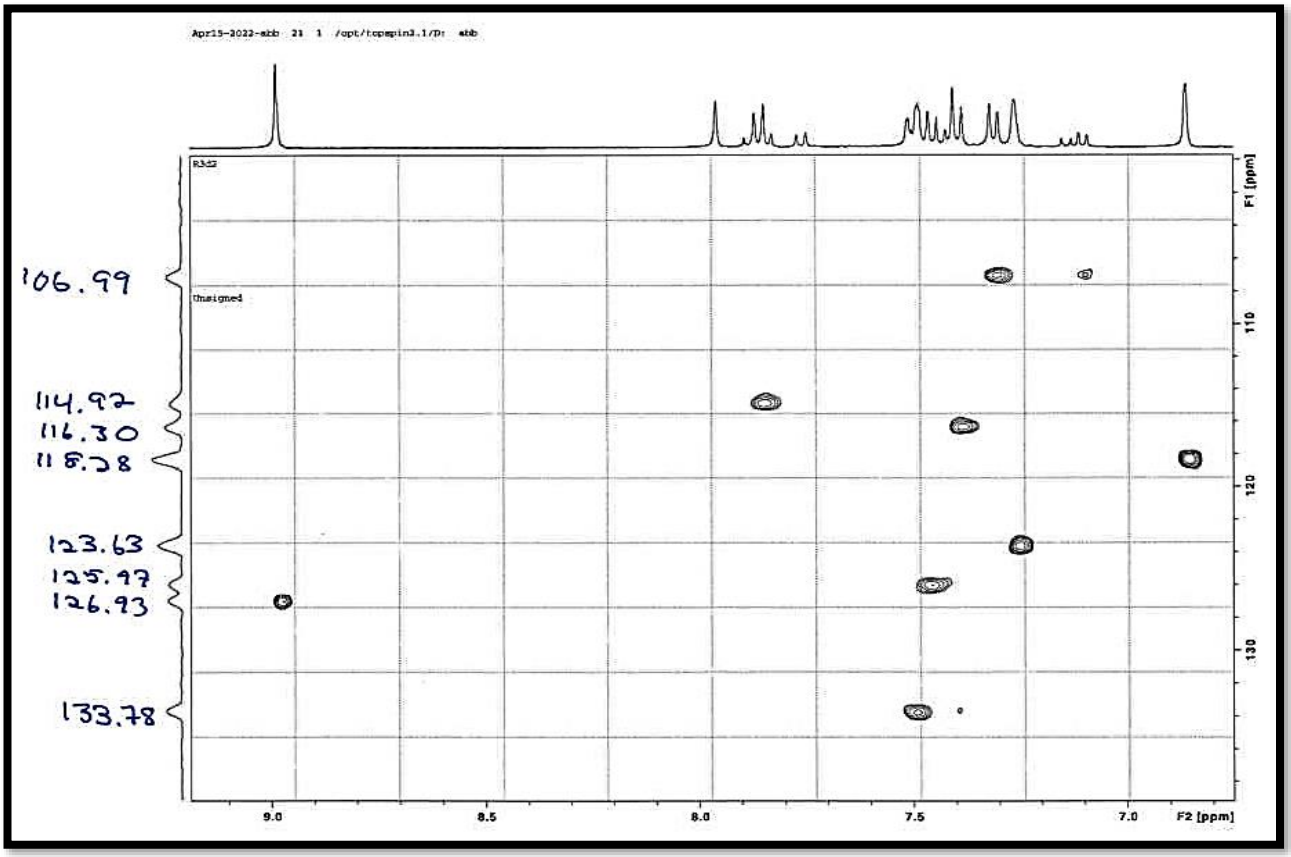

Figure S60. Part of the ^{1}H-^{13}C HSQC spectrums for compound **7a.**

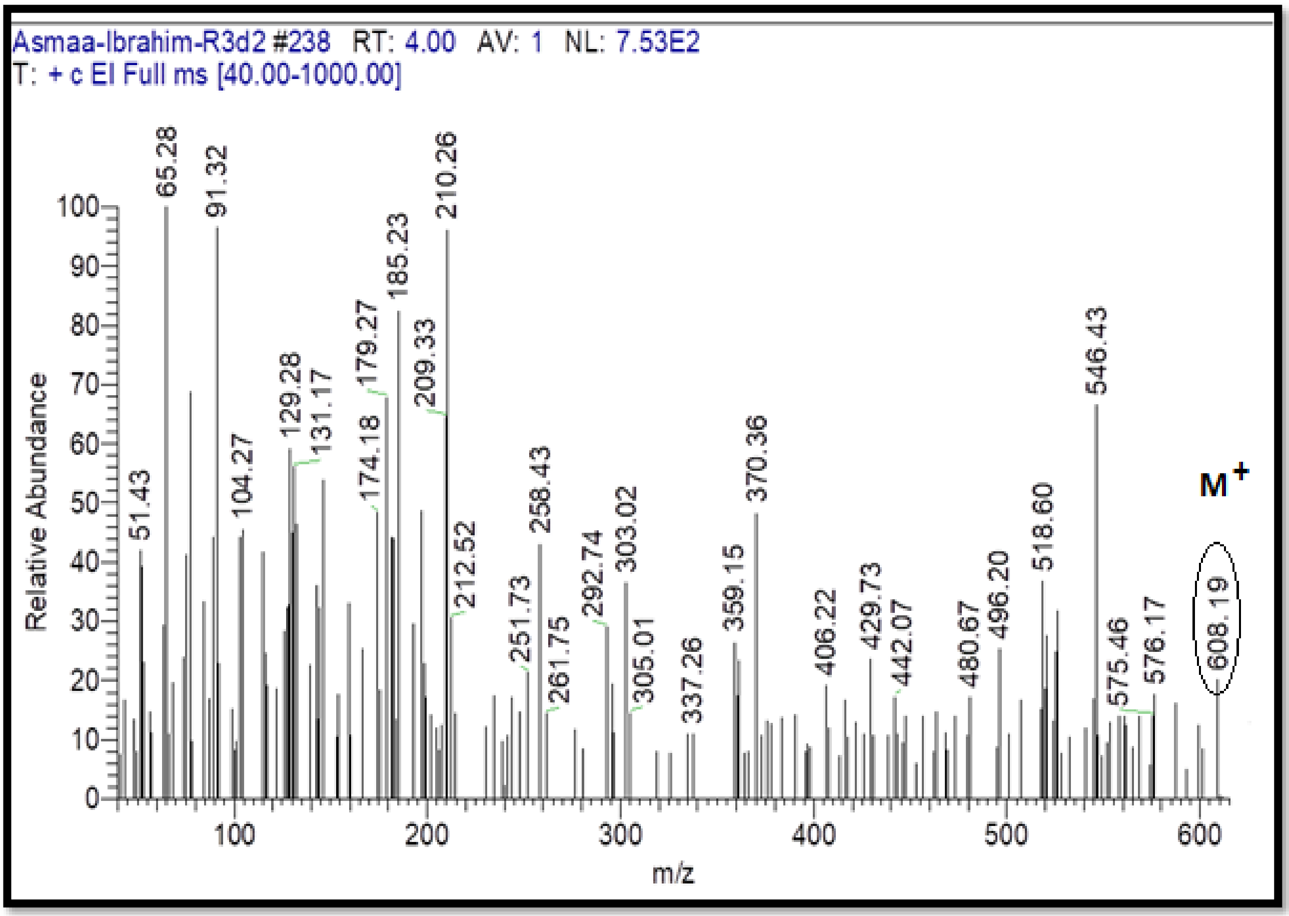

Figure S61. Mass pectrometry for compound **7a.**

Spectral data for compound 7b.

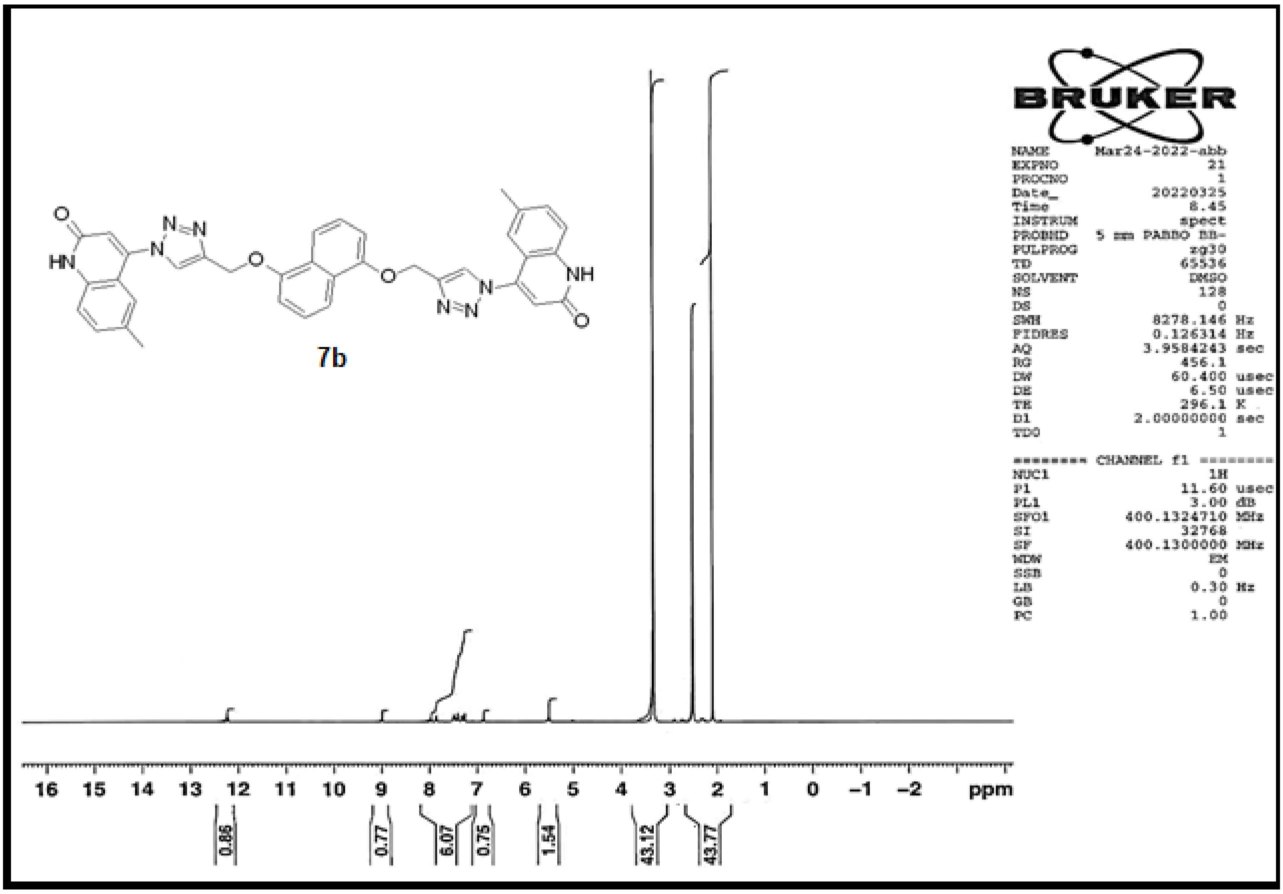

Figure S62. ^{1}H NMR spectrums for compound **7b.**

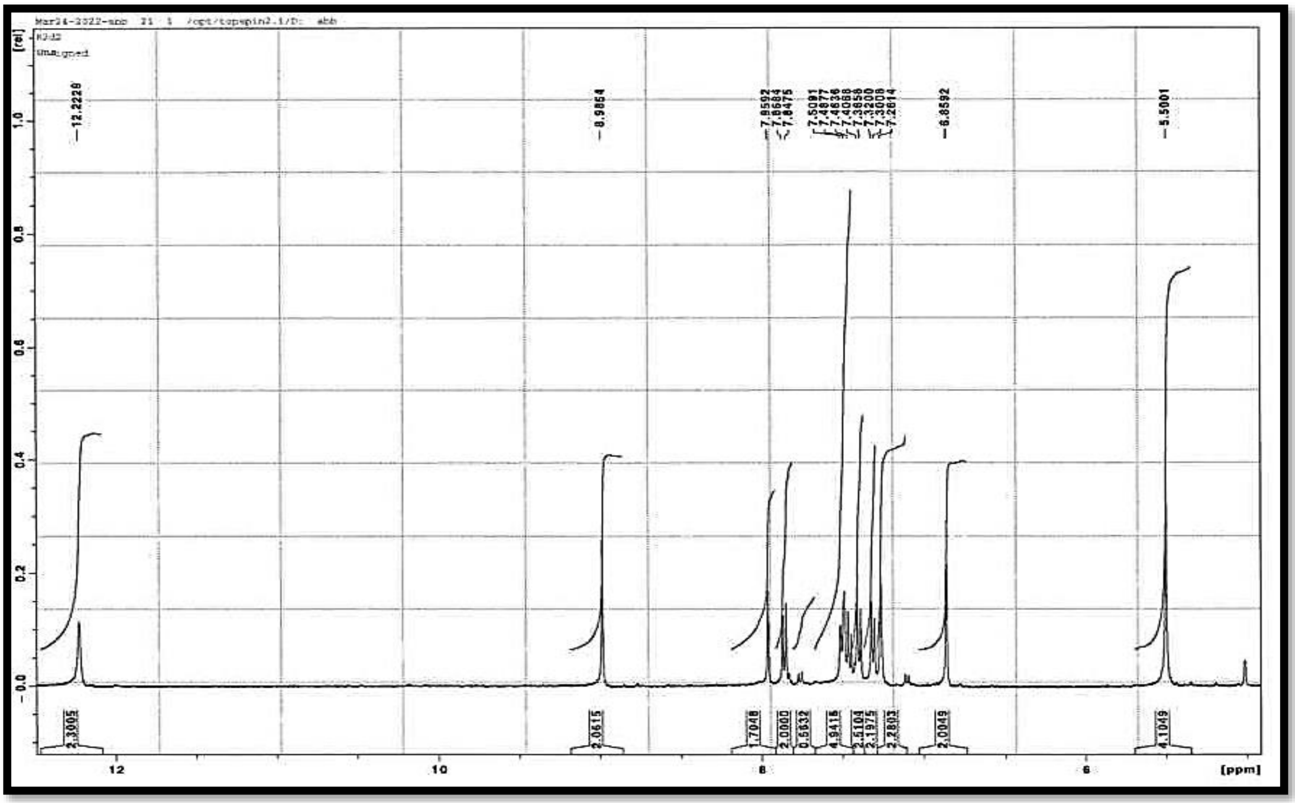

Figure S63. Part of the ^{1}H NMR spectrums for compound **7b.**

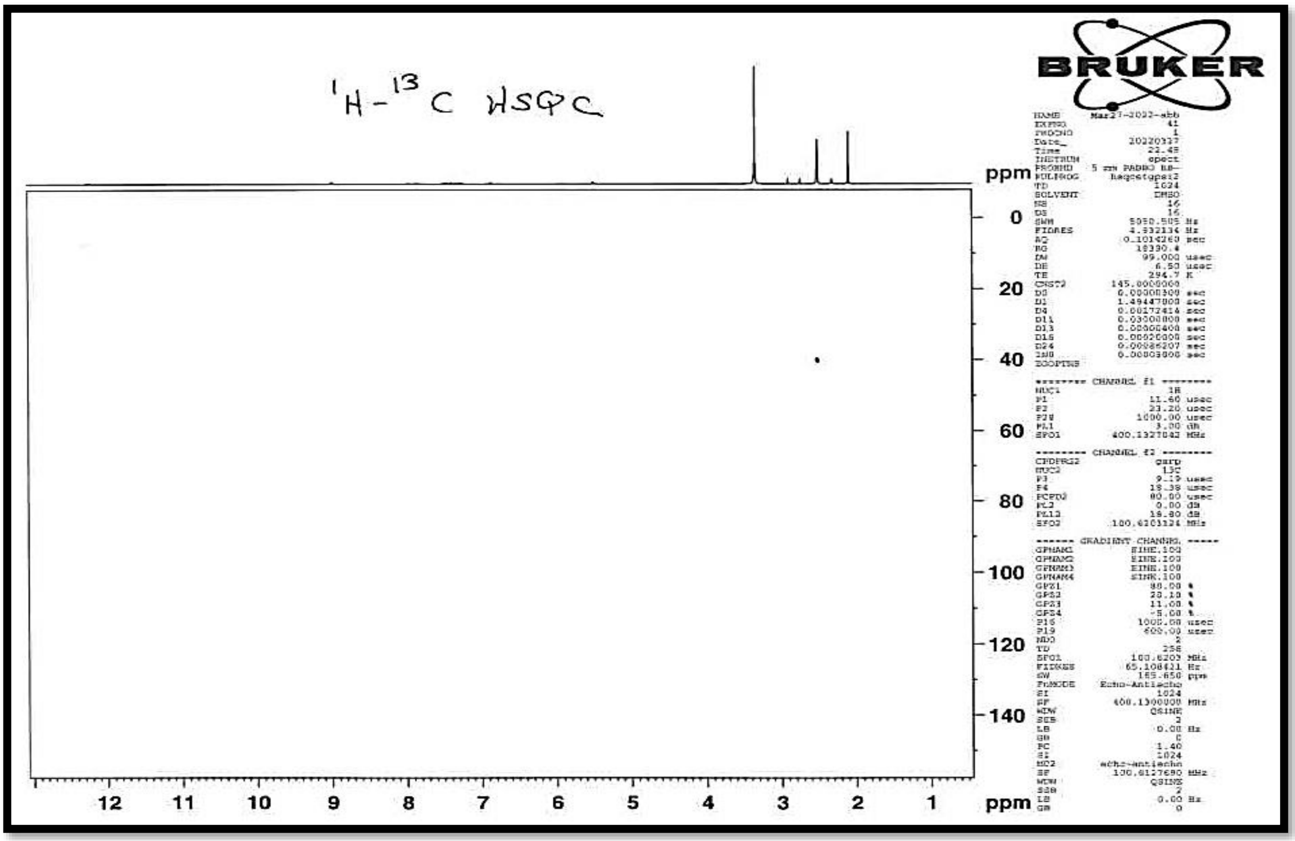

Figure S64. ^{1}H-^{13}C HSQC spectrums for compound **7b.**

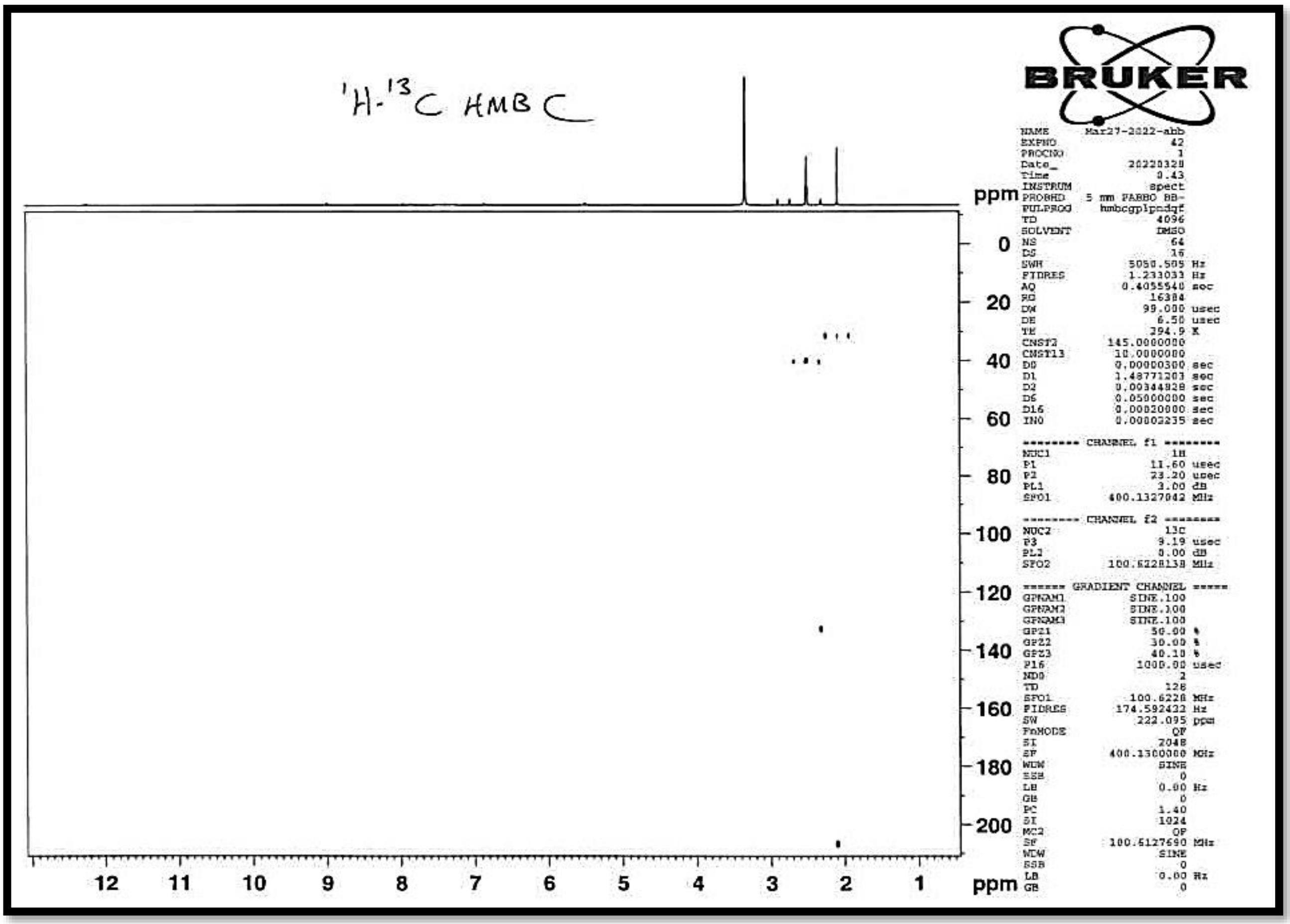

Figure S65. ^{1}H-^{13}C HMBC spectrums for compound **7b.**

Spectral data for compound 7c.

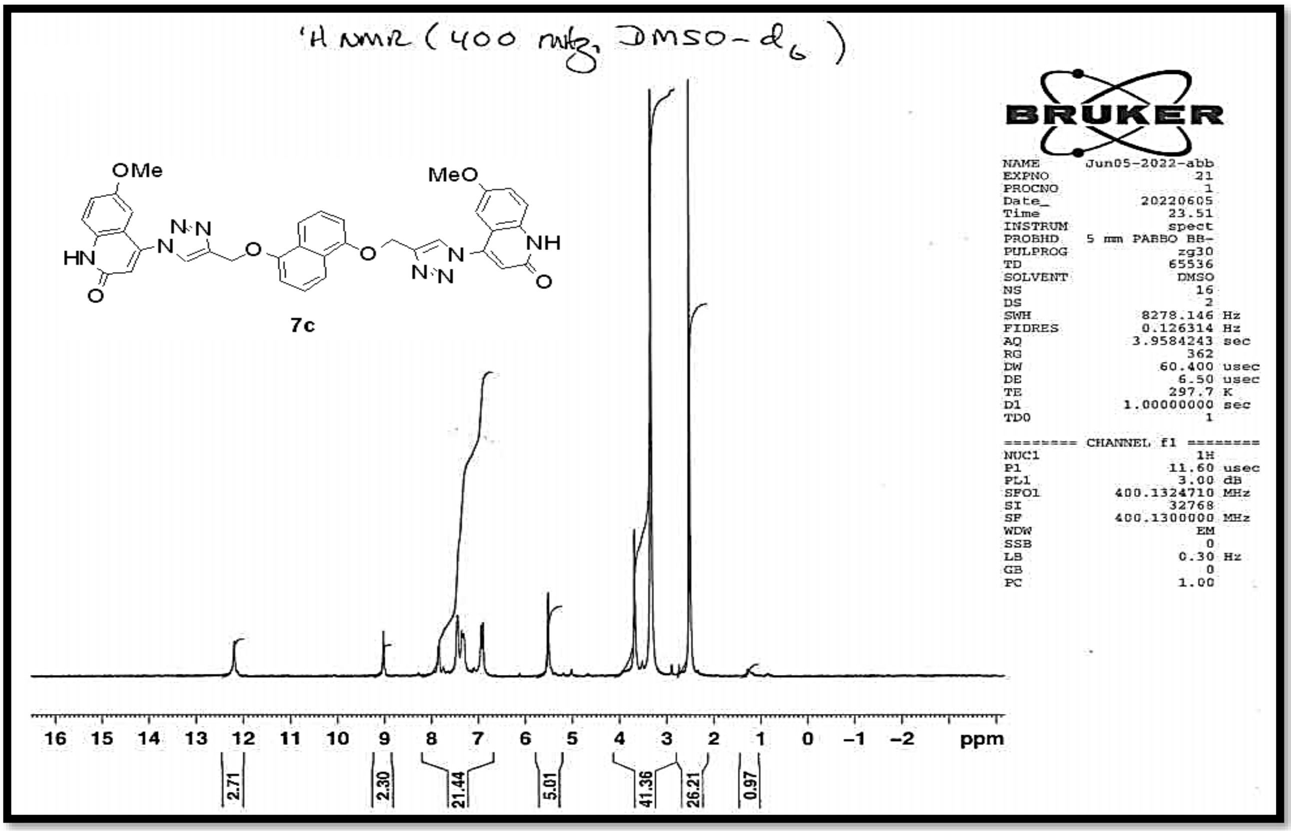

Figure S66. ^{1}H NMR spectrums for compound **7c.**

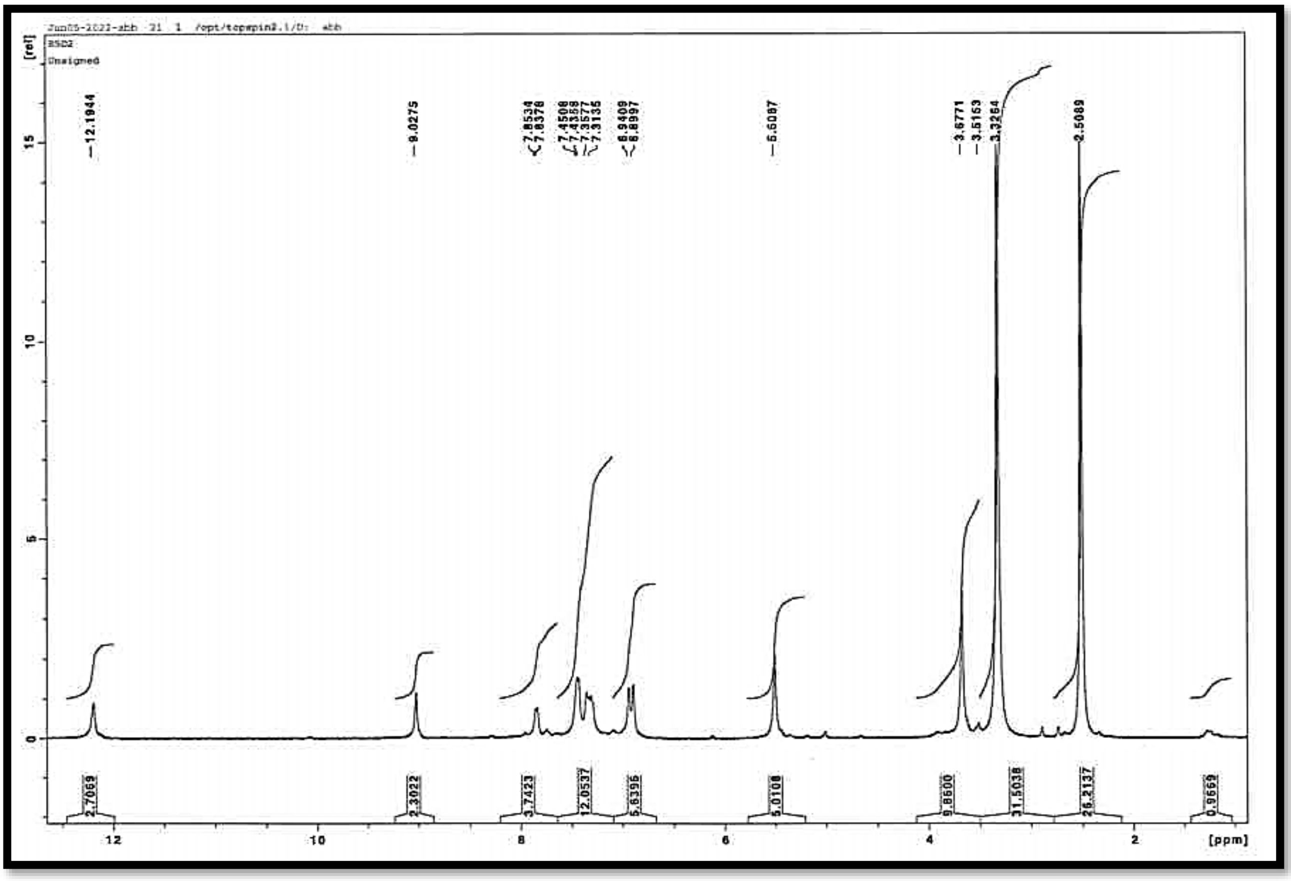

Figure S67. Part of the ^{1}H NMR spectrums for compound **7c.**

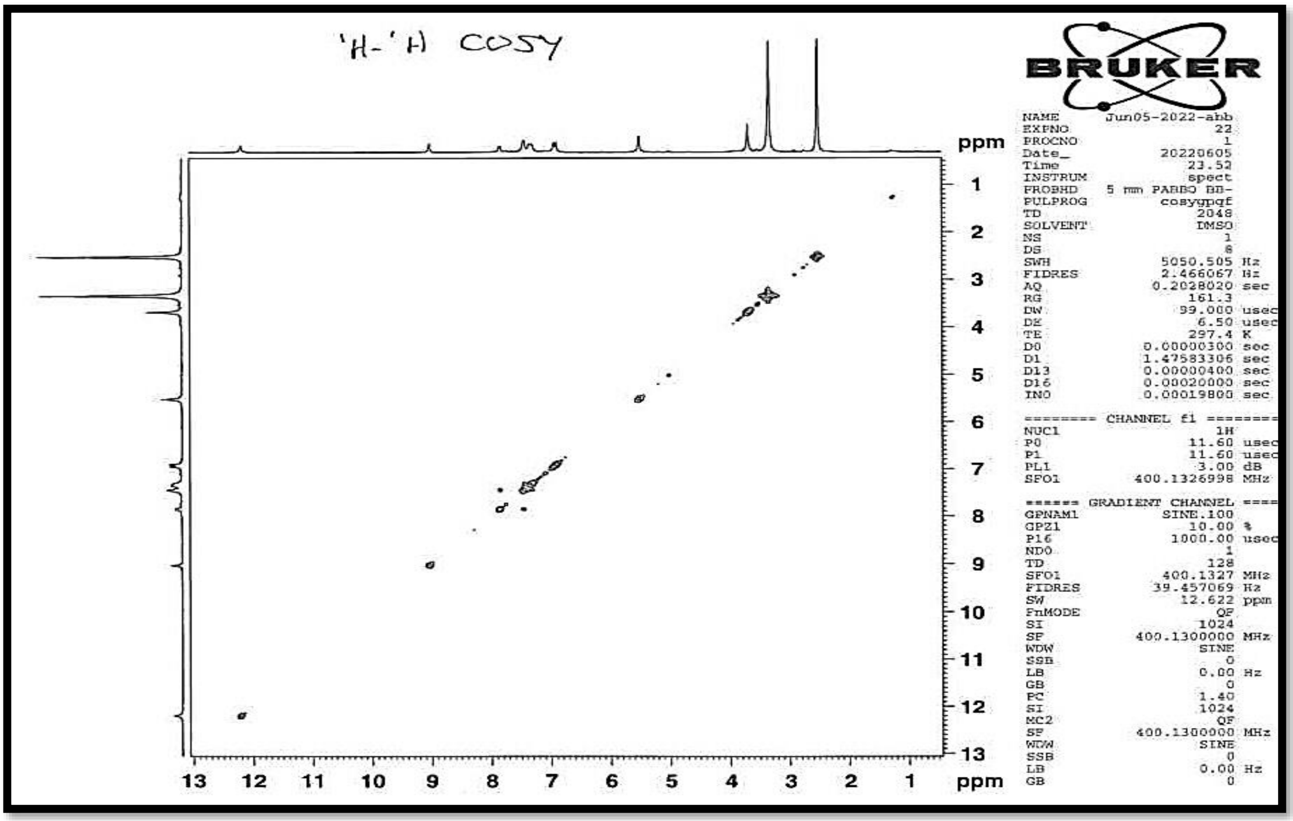

Figure S68. ^{1}H-^{1}H Cosy spectrums for compound **7c.**

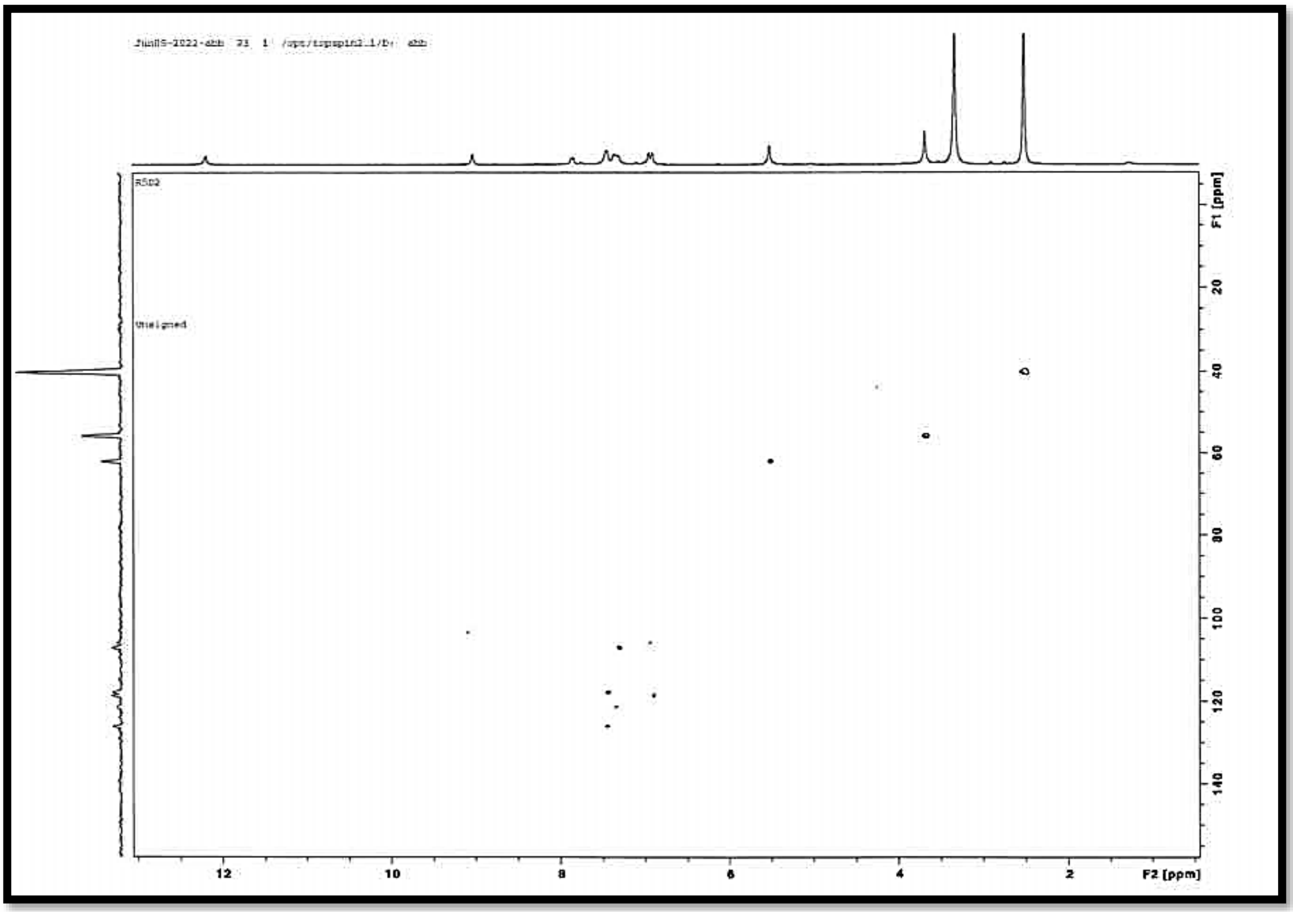

Figure S69. ^{1}H-^{13}C HSQC spectrums for compound **7c.**

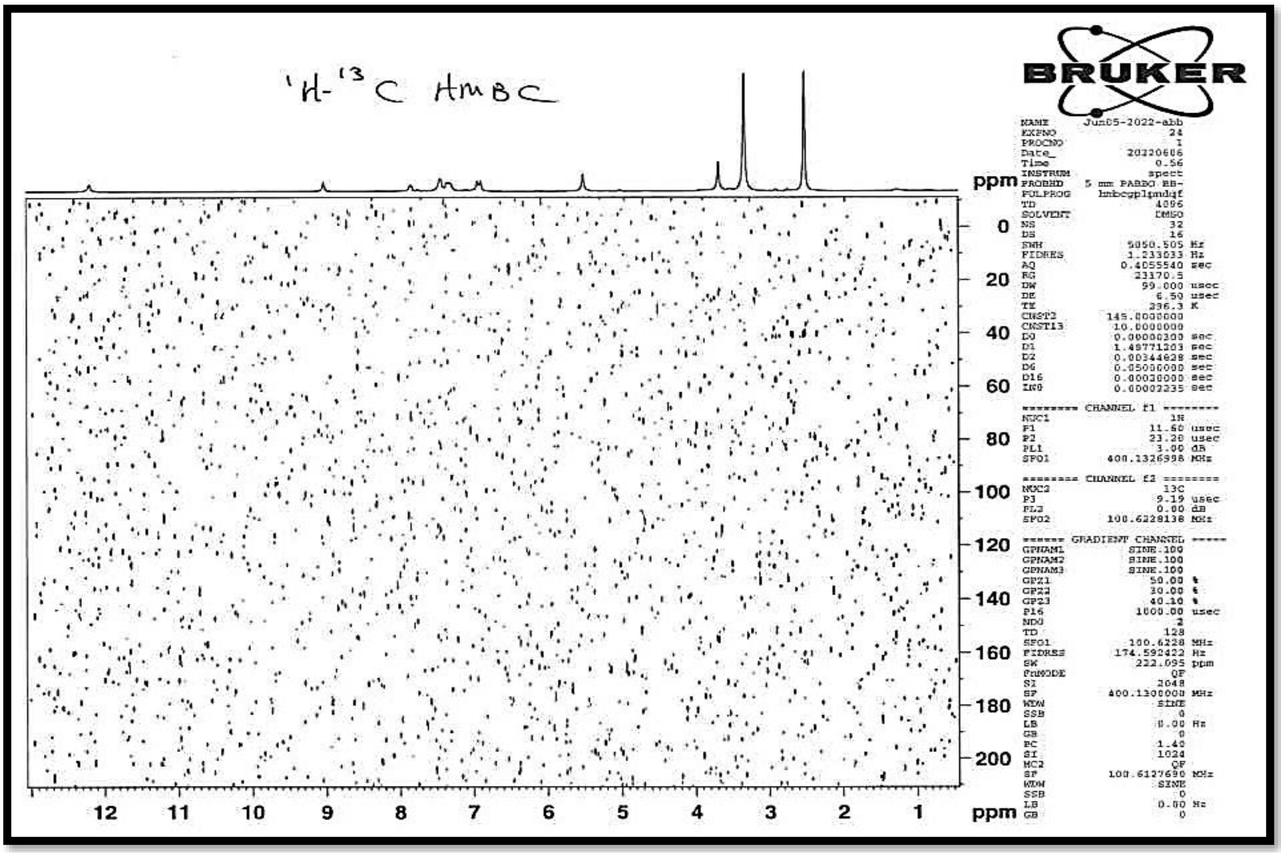

Figure S70. ^{1}H-^{13}C HMBC spectrums for compound **7c.**

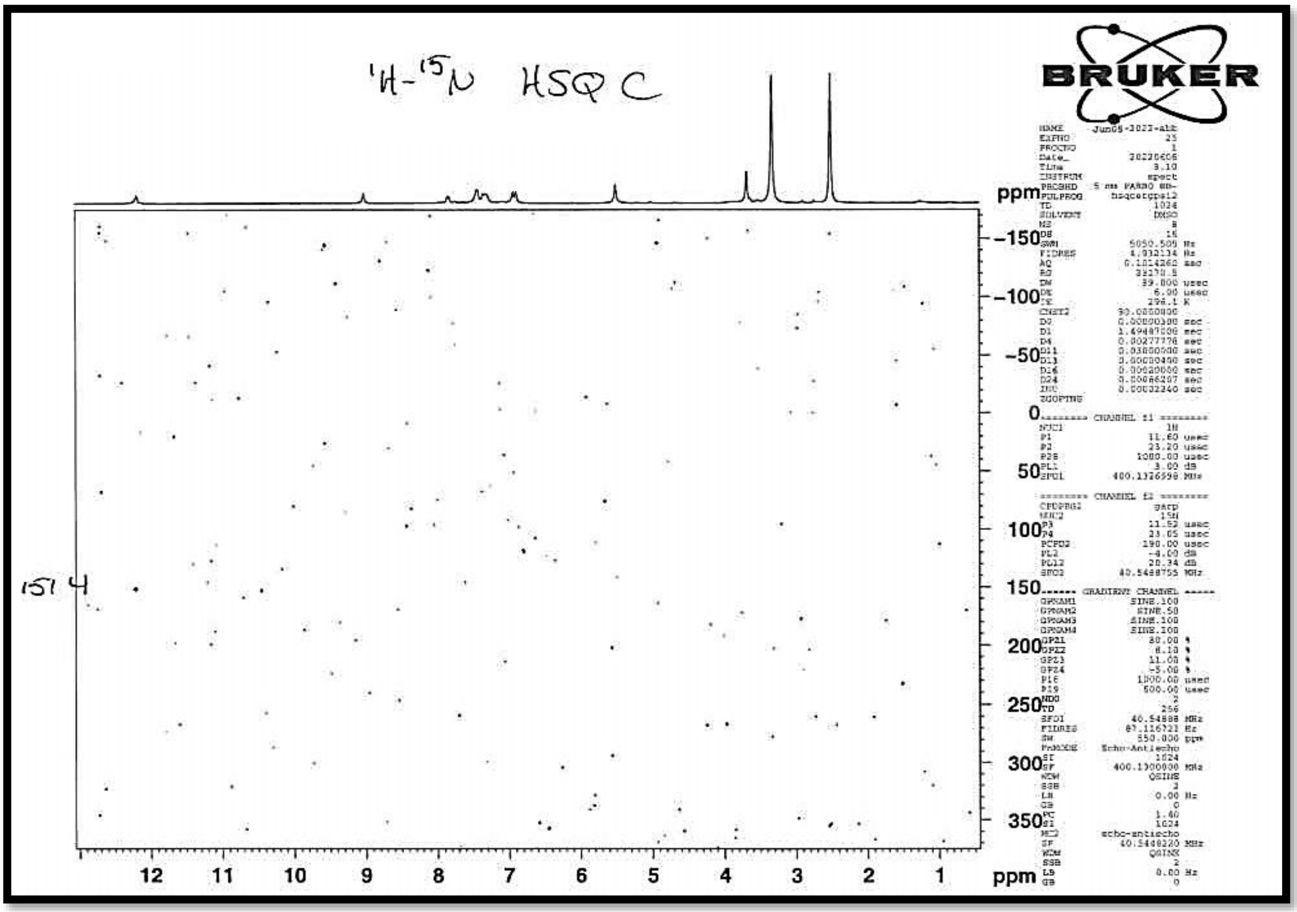

Figure S71. ^{1}H-^{15}N HSQC spectrums for compound **7c.**

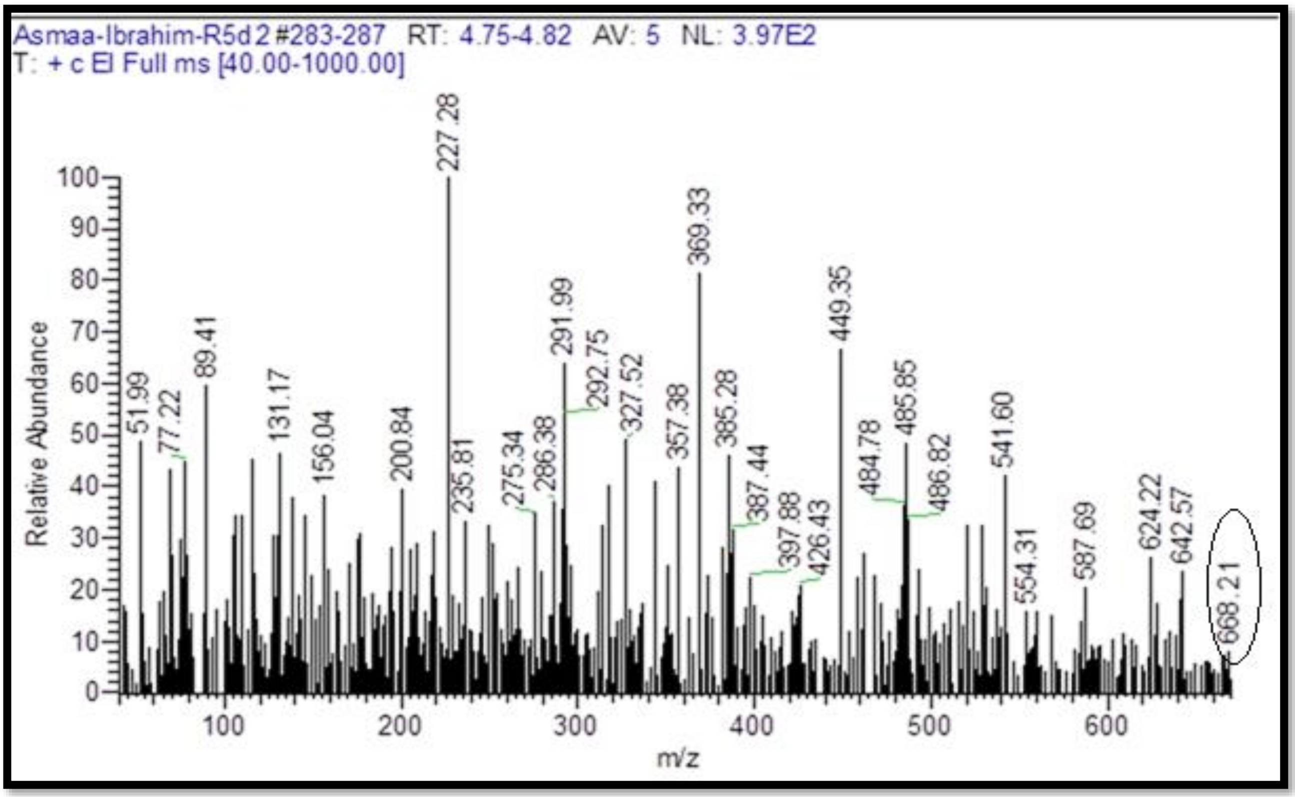

Figure S72. Mass spectrometry for compound **7c.**

Spectral data for compound 7d.

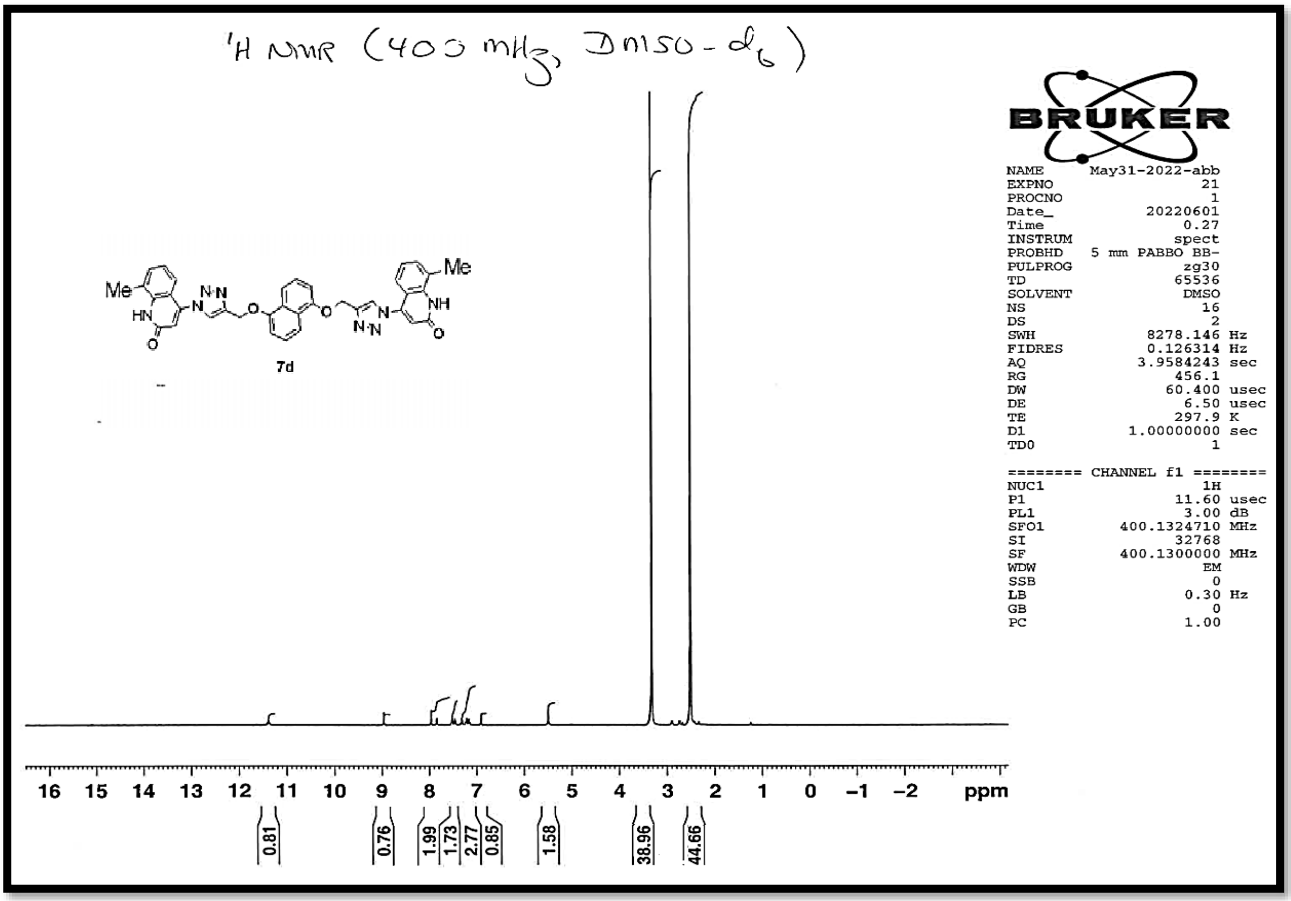

Figure S73. ¹H NMR spectrums for compound **7d.**

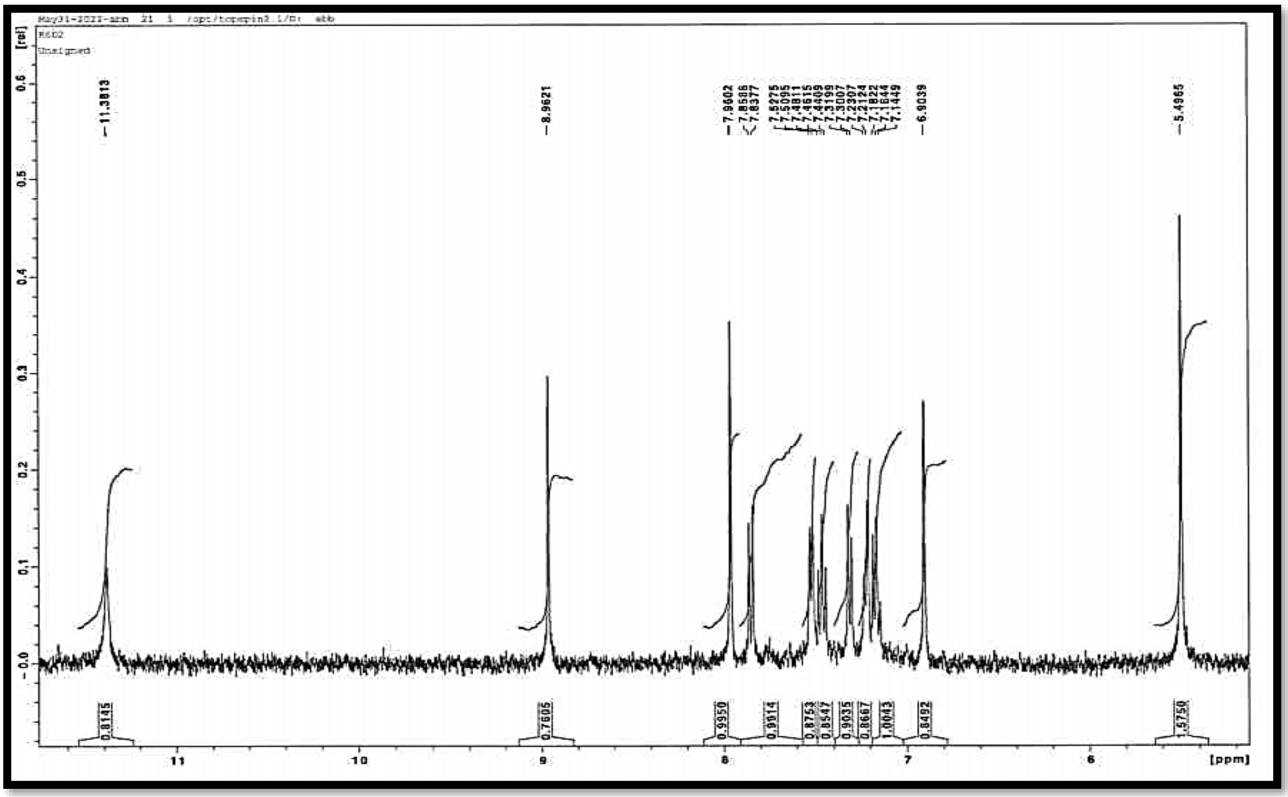

Figure S74. Part of the ^{1}H NMR spectrums for compound **7d.**

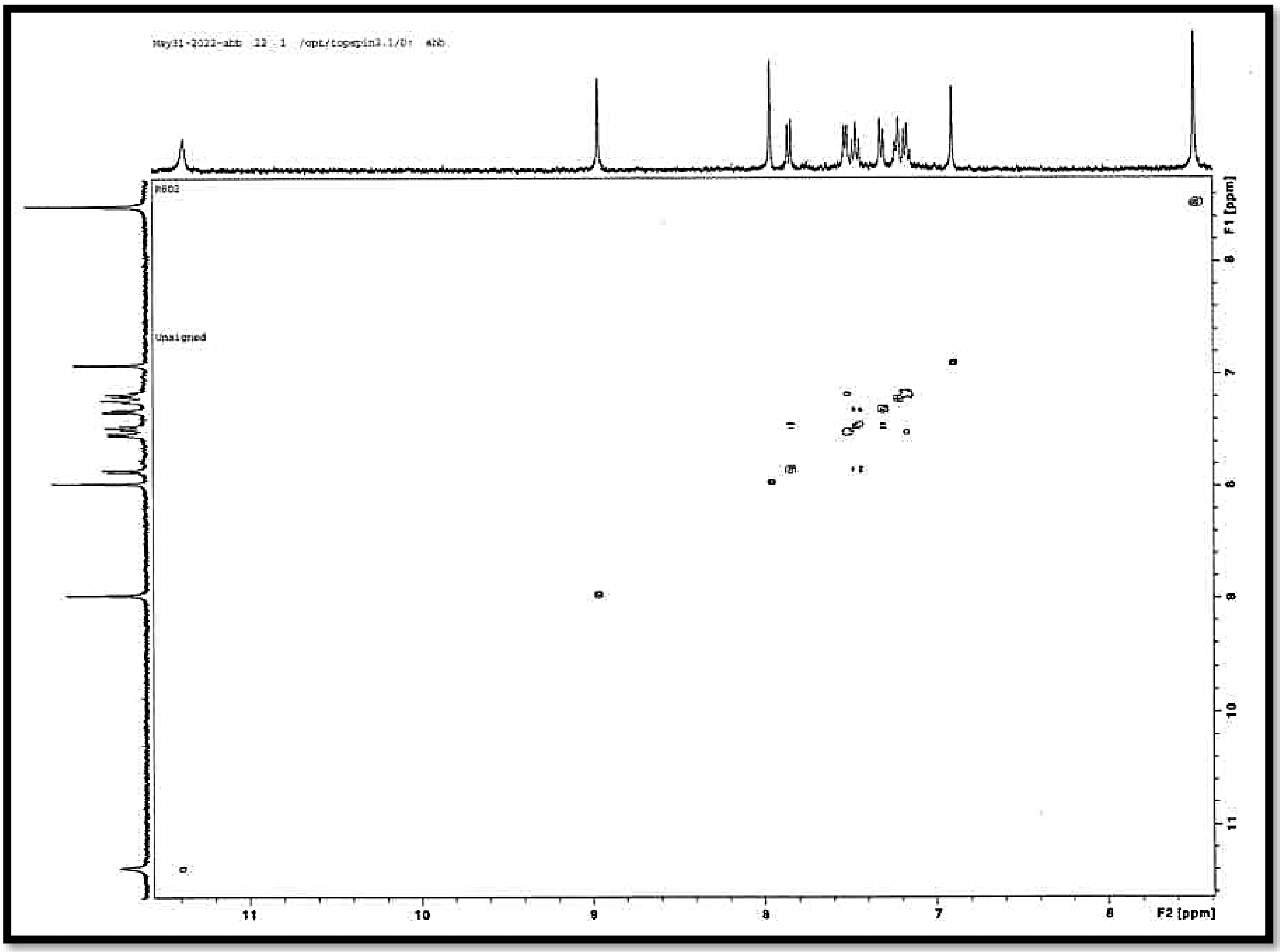

Figure S75. Part of the ^{1}H-^{1}H Cosy spectrums for compound **7d.**

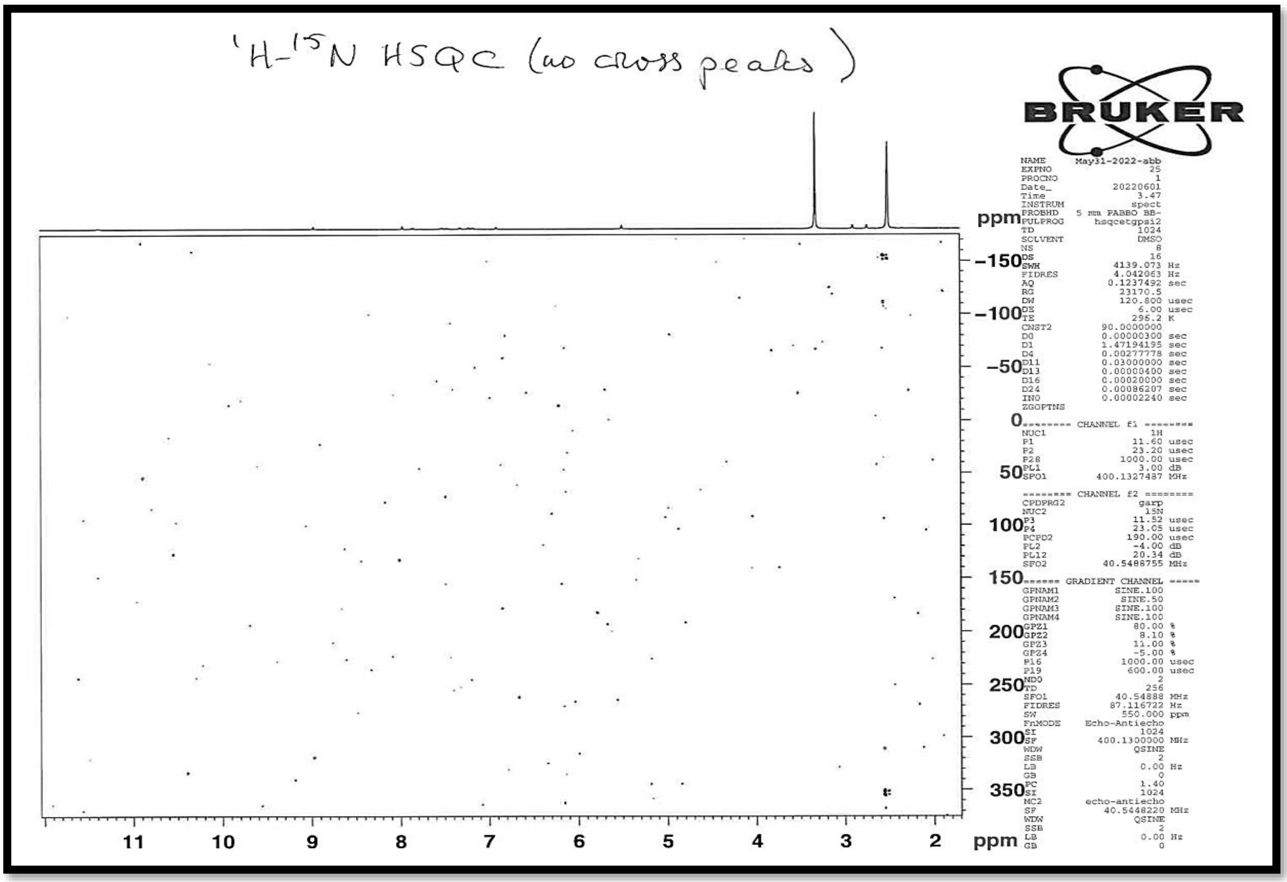

Figure S76. Part of the ^{1}H-^{15}N HSQC spectrums for compound **7d.**

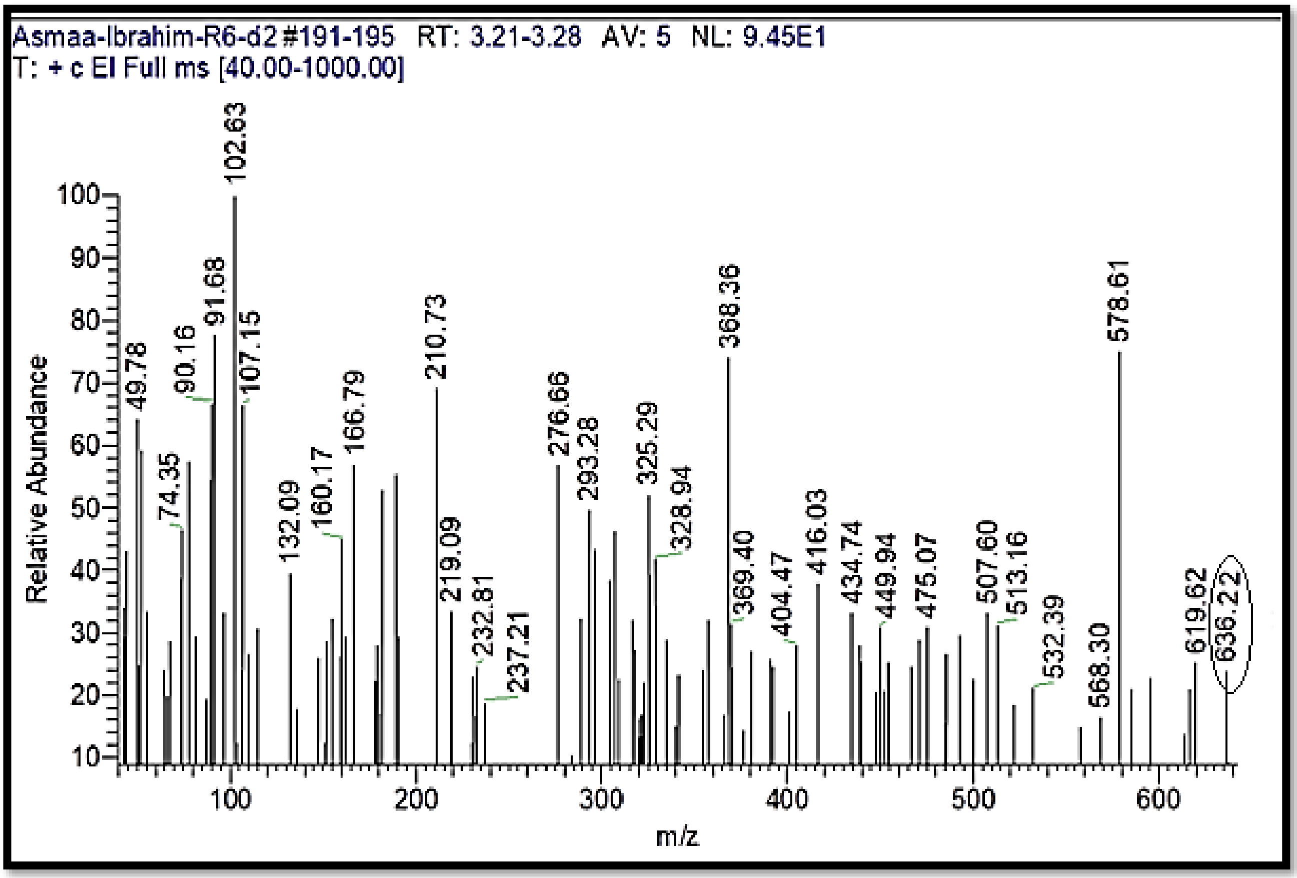

Figure S77. Mass spectrometry for compound **7d.**

Spectral data for compound 7e

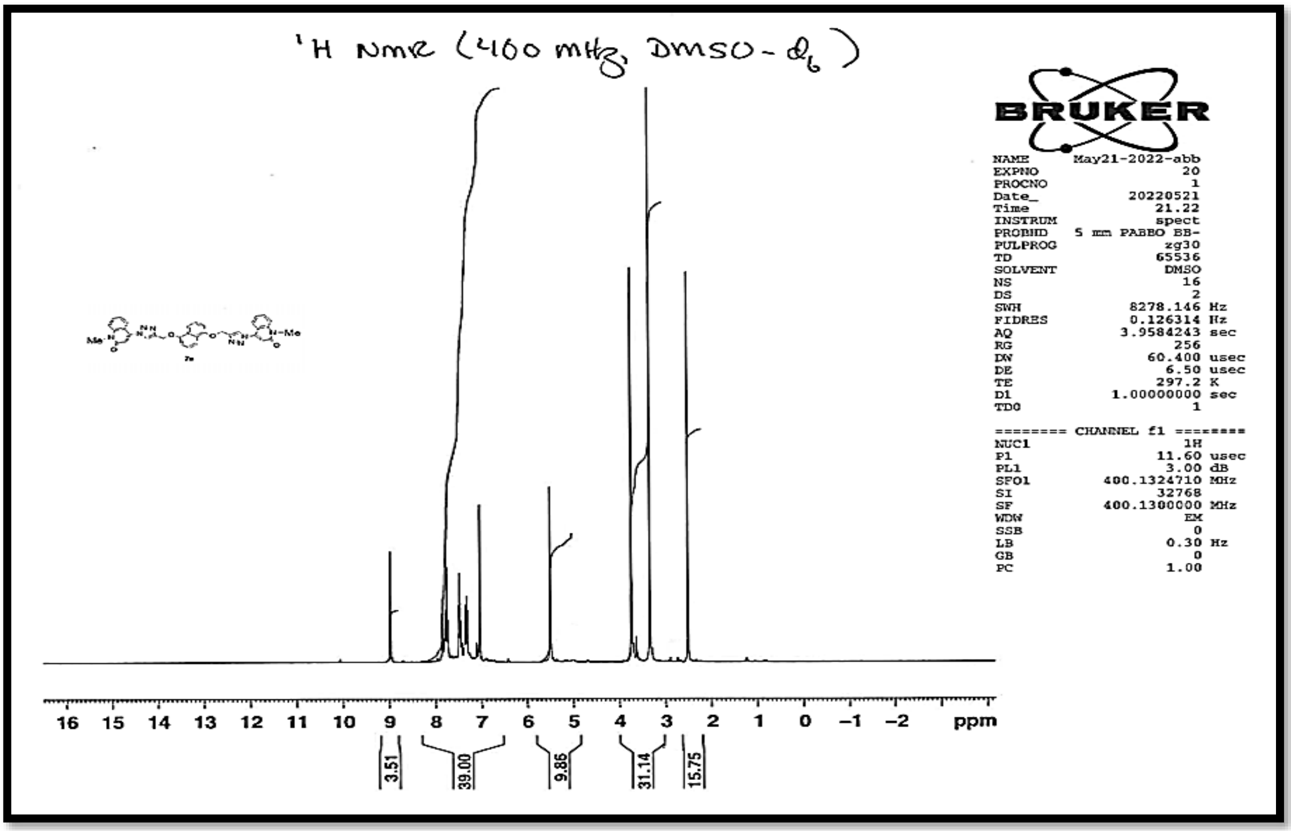

Figure S78. ^{1}H NMR spectrums for compound **7e.**

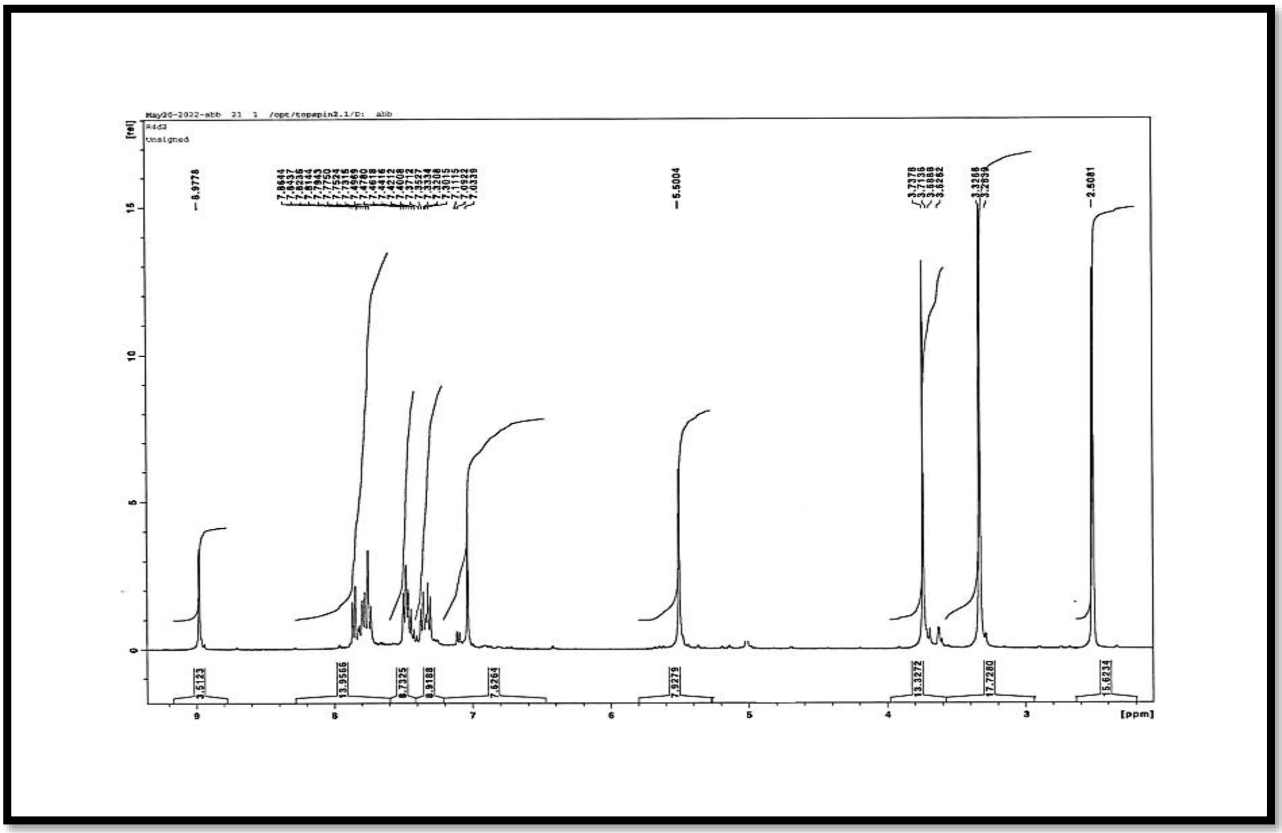

Figure S79. Part of the ^{1}H NMR spectrums for compound **7e.**

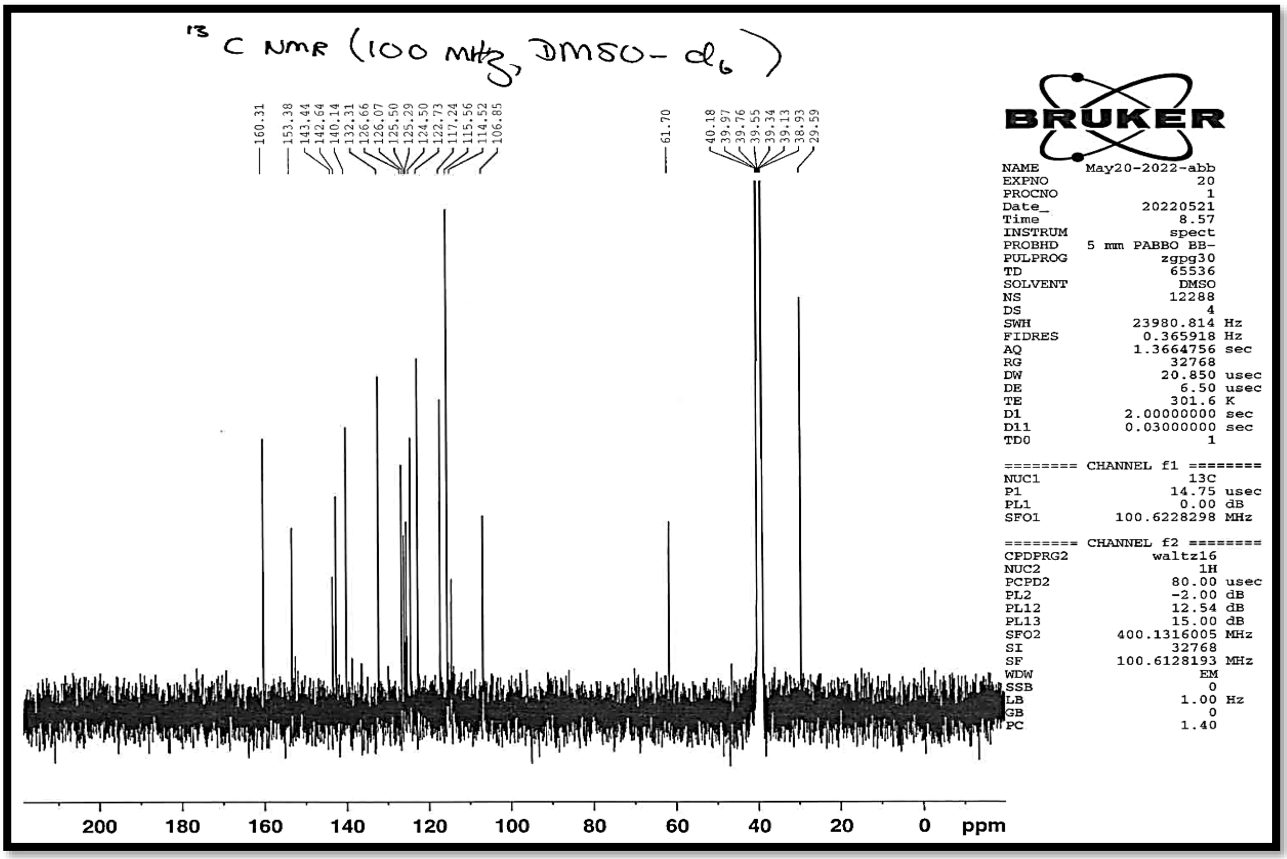

Figure S80. ^{13}C NMR spectrums for compound **7e.**

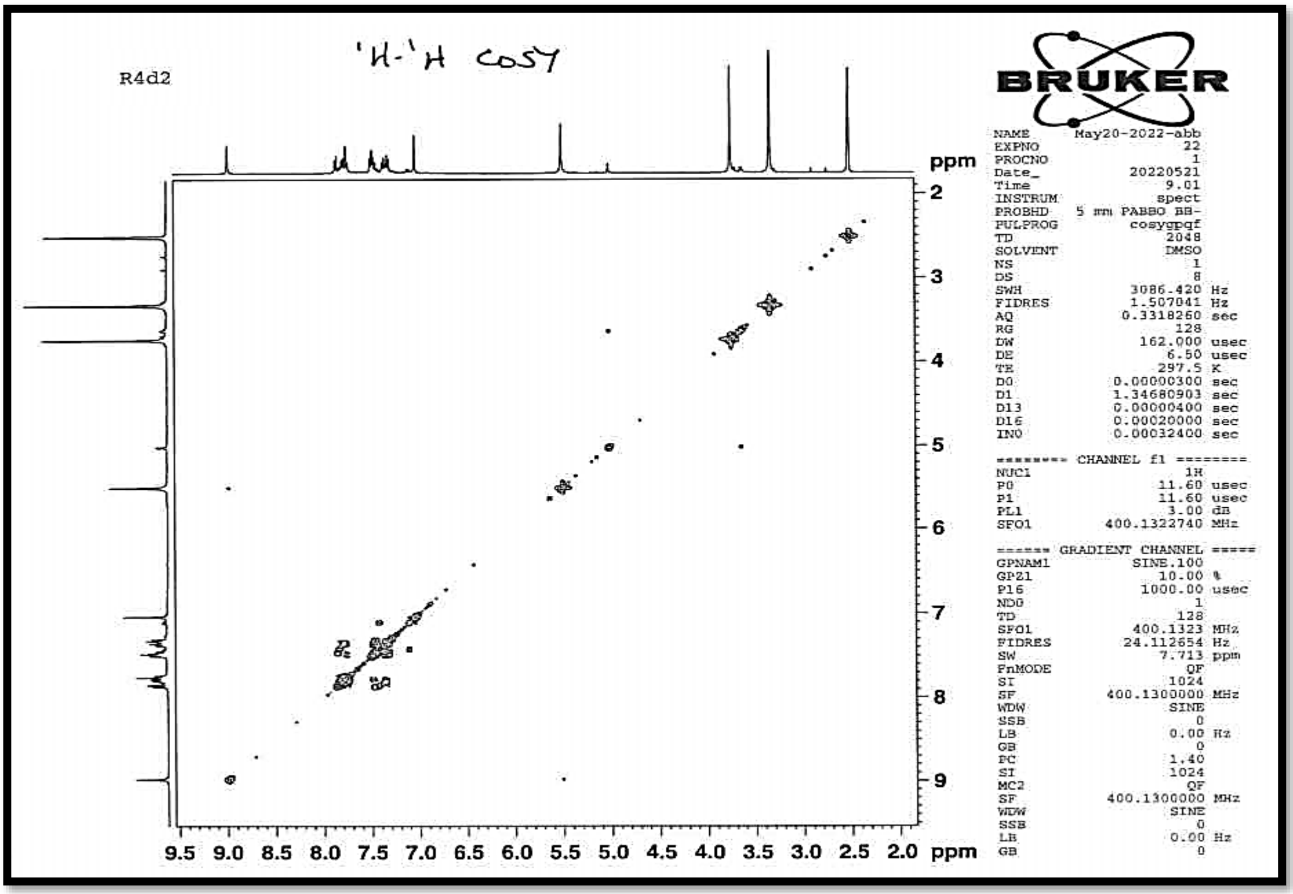

Figure S81. ^{1}H-^{1}H Cosy spectrums for compound **7e.**

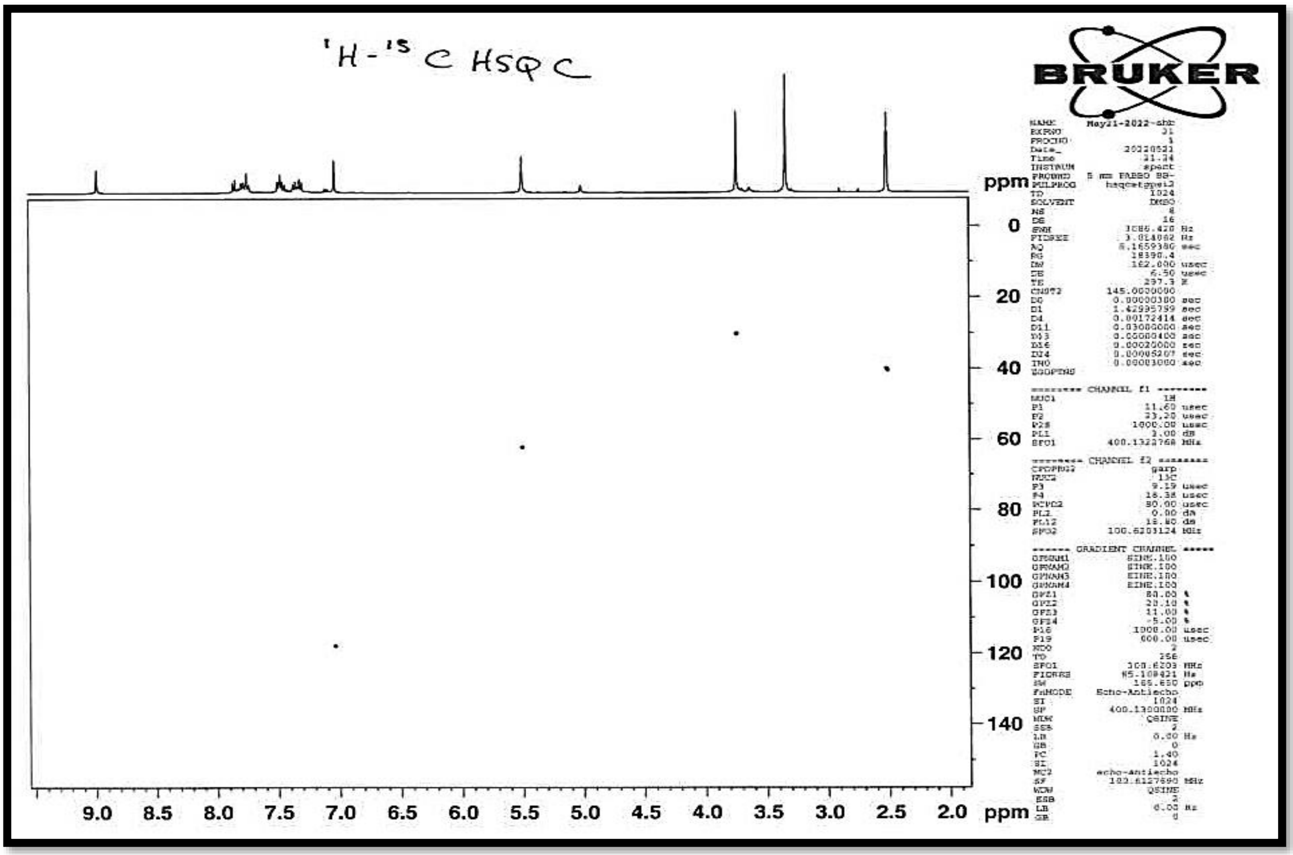

Figure S82. ^{1}H-^{13}C HSQC spectrums for compound **7e.**

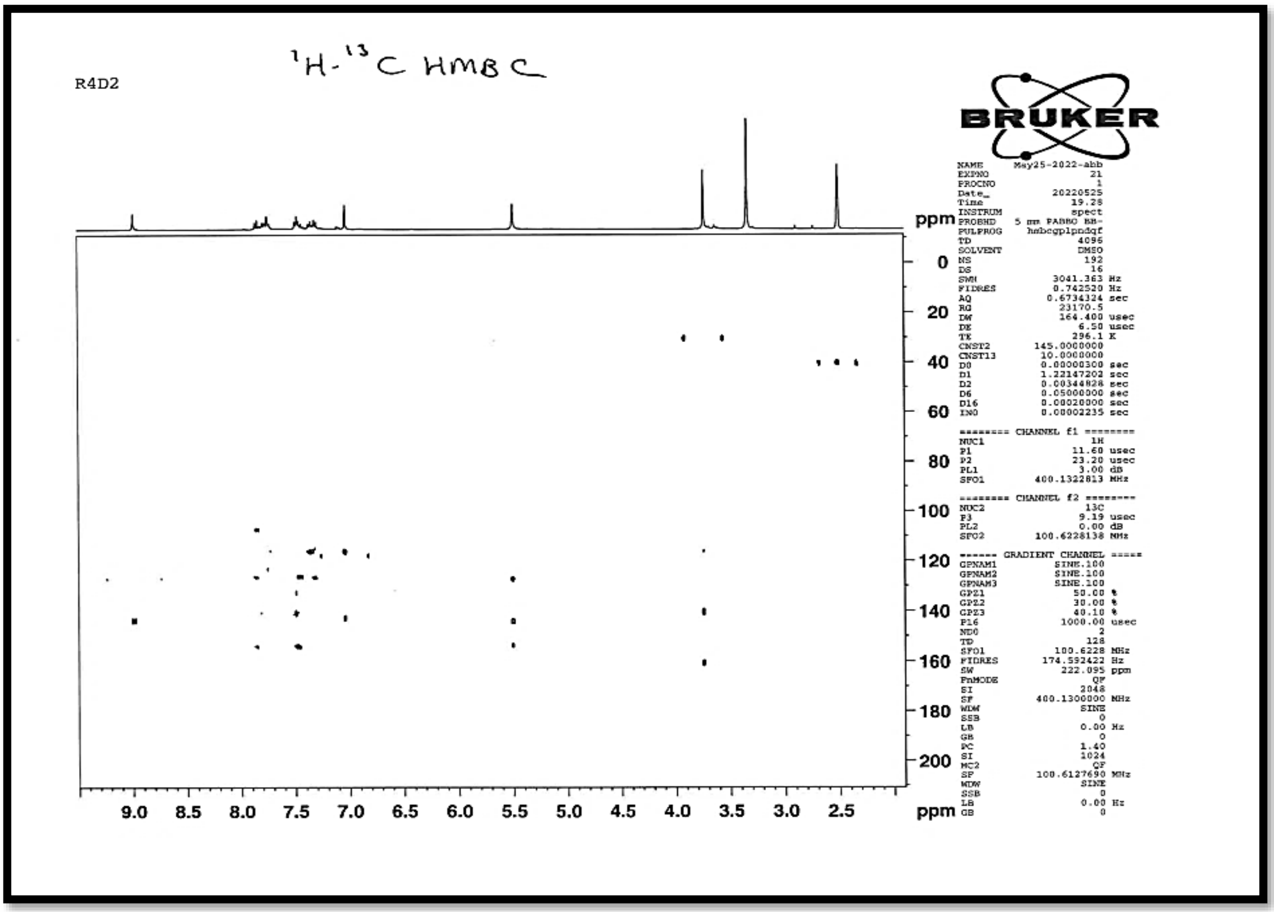

Figure S83. ^{1}H-^{13}C HMBC spectrums for compound **7e.**

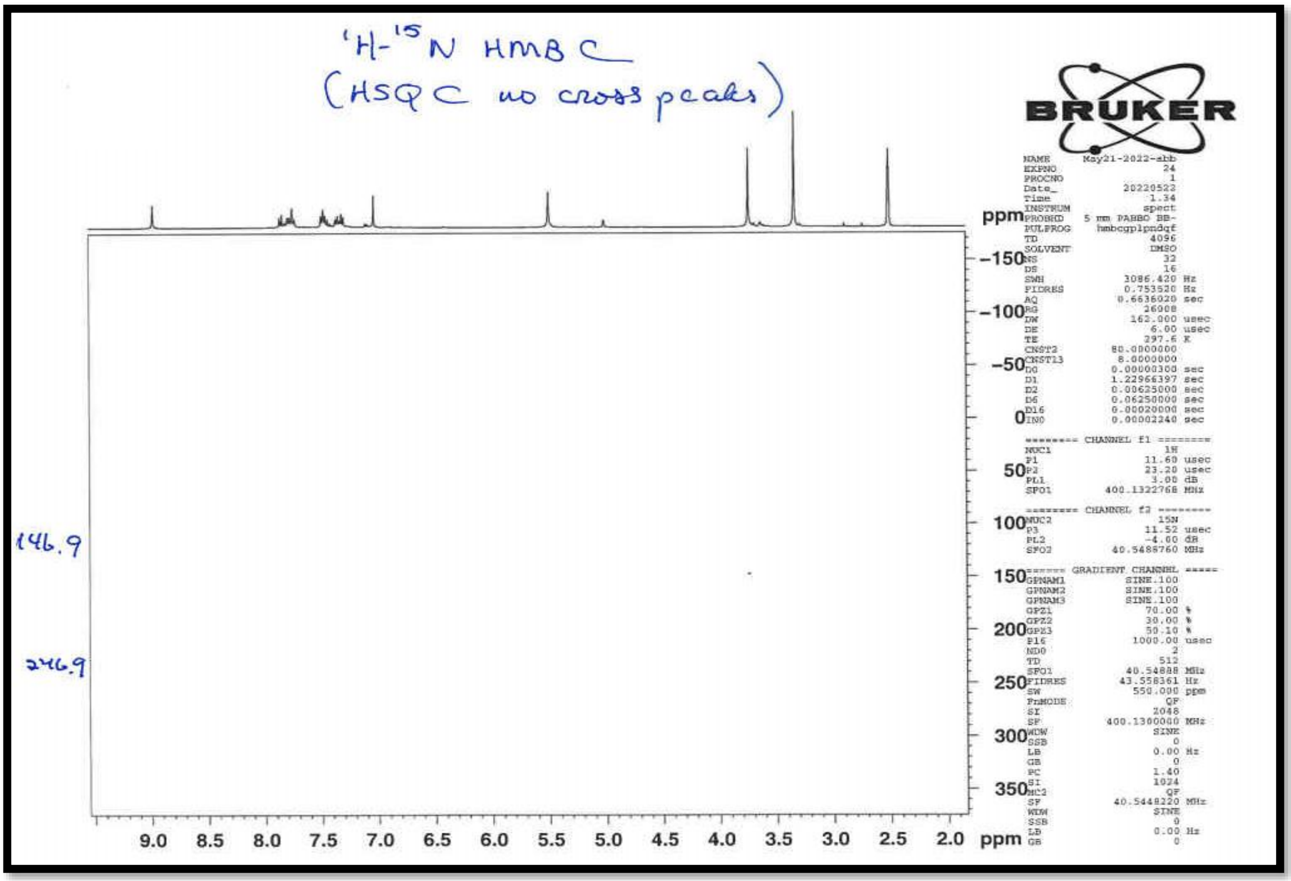

Figure S84. ^{1}H-^{13}C HSQC spectrums for compound **7e.**

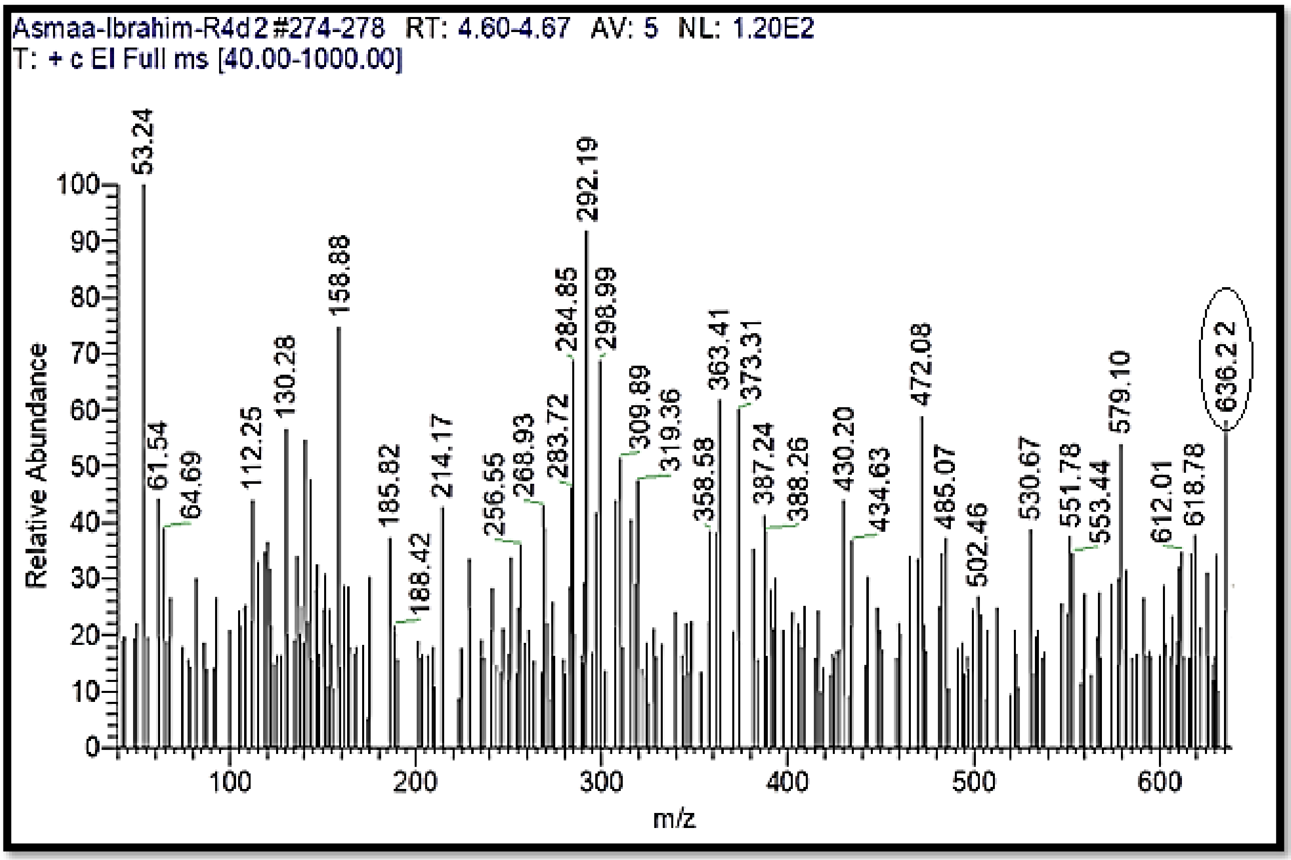

Figure S85. Mass spectrometry for compound **7e.**

Appendix SA

4. Experimental

4.1. Chemistry

Melting points were determined on Stuart electrothermal melting point apparatus and were uncorrected. All reactions were monitored with thin-layer chromatography (TLC) on Merck alumina-backed TLC plates and visualized under UV light. NMR spectra were measured in DMSO-d_6 on a Bruker AV-400 spectrometer (400 MHz for ^{1}H, 100 MHz for ^{13}C, and 40.54 MHz for ^{15}N) at the Chemistry Department, Florida Institute of Technology, 150 W University Blvd, Melbourne, FL 32901, USA. Chemical shifts are expressed in δ (ppm) versus internal Tetramethylsilane (TMS) = 0 ppm for ^{1}H and ^{13}C, and external liquid ammonia = 0 ppm for ^{15}N. Coupling constants are stated in Hz. Correlations were established using ^{1}H-^{1}H COSY, ^{1}H-^{13}C, and ^{1}H-^{15}N HSQC and HMBC experiments. Chemical shifts (δ) are reported in parts per million (ppm) relative to Tetramethylsilane (TMS) as the internal standard, and the coupling constants (*J*) are reported in Hertz (Hz). Splitting patterns are denoted as follows: singlet (s), broad (b), broad of singlet (bs), doublet (d), doublet of doublets (dd), multiplet (m), triplet (t), quartet (q) and doublet of triplets (dt). Elemental analyses were carried out on a Perkin Elmer device at the Microanalytical Institute of Organic Chemistry, Karlsruhe Institute of Technology, Karlsruhe, Germany, and the mass spectrometry was recorded on a Finnigan Fab 70 eV at Al-Azhar University, Egypt.

4.2. Biological evaluation

4.2.1. Cytotoxic activity using MTT Assay and evaluation of IC_{50}

4.2.1.1. MTT assay

MTT assay was carried out to study the effect of compounds on mammary epithelial cells (MCF-10A). The medium in which cells were propagated contained Dulbecco's modified Eagle's medium (DMEM)/ Ham's F-12 medium (1:1) supplemented with epidermal growth factor (20 ng/mL), hydrocortisone (500 ng/mL), insulin (10 μg/mL), 2 mM glutamine and 10% foetal calf serum. After every 2-3 days, the cells were passaged using trypsin ethylenediamine tetra acetic acid (EDTA). The cells were seeded at a density of 10^4 cells mL^{-1} in flat-bottomed culture plates containing 96 wells each. After 24 h, medium was removed from the plates and the compounds in (in 0.1% DMSO) were added (in 200 μL medium to yield a final concentration of 0.1% v/v) to the wells of plates. A single compound was designated with four wells followed by incubation of plates for 96h at 37°C. After incubation, medium was removed completely from the plates followed by addition of MTT (0.4 mg/mL in medium) to each well and subsequent incubation of plates for 3h. MTT (along with the medium) was removed and DMSO (150μL) was added to each well of the culture plates, followed by vortexing and subsequent measurement of absorbance (at 540 nm) using microplate reader. The data are shown as percentage inhibition of proliferation in comparison with controls containing 0.1% DMSO.

4.2.1.2. Assay for antiproliferative effect

To explore the antiproliferative potential of compounds MTT assay was performed according to previously reported procedure using different cell lines to explore the antiproliferative potential of compounds propidium iodide fluorescence assay was performed using different cell lines. To calculate the total nuclear DNA, a fluorescent dye (propidium iodide, PI) is used which can attach to the DNA, thus offering a quick and precise technique. PI cannot pass through the cell membrane and its signal intensity can be considered as directly proportional to quantity of cellular DNA. Cells whose

cell membranes are damaged or have changed permeability are counted as dead ones. The assay was performed by seeding the cells of different cell lines at a density of 3000-7500 cells/well (in 200µl medium) in culture plates followed by incubation for 24h at 37 °C in humidified 5% CO_2/95% air atmospheric conditions. The medium was removed; the compounds were added to the plates at 10 µM concentrations (in 0.1% DMSO) in triplicates, followed by incubation for 48 h. DMSO (0.1%) was used as control. After incubation, medium was removed followed by the addition of PI (25 µl, 50µg/mL in water/medium) to each well of the plates. At -80 °C, the plates were allowed to freeze for 24 h, followed by thawing at 25°C. A fluorometer (Polar-Star BMG Tech) was used to record the readings at excitation and emission wavelengths of 530 and 620 nm for each well. The percentage cytotoxicity of compounds was calculated using the following formula:

$$\% \text{ Cytotoxicity} = \frac{A_C - A_{TC}}{A_C} \times 100$$

Where A_{TC}= Absorbance of treated cells and A_C= Absorbance of control. Erlotinib was used as positive control in the assay.

4.2.1.3. EGFR inhibitory assay

EGFR-TK assay was performed to evaluate the inhibitory potency of novel compounds against EGFR. Baculoviral expression vectors including pBlueBacHis2B and pFASTBacHTc were used separately to clone 1.6 kb cDNA coding for EGFR cytoplasmic domain (EGFR-CD, amino acids 645–1186). 5′ upstream to the EGFR sequence comprised a sequence that encoded $(His)_6$. Sf-9 cells were infected for 72h for protein expression. The pellets of Sf-9 cells were solubilized in a buffer containing sodium vanadate (100 µM), aprotinin (10 µg/mL), triton (1%), HEPES buffer (50mM), ammonium molybdate (10 µM), benzamidine HCl (16 µg/mL), NaCl (10 mM), leupeptin (10 µg/mL) and pepstatin (10 µg/mL) at 0°C for 20 min at pH 7.4, followed by centrifugation for 20 min. To eliminate the non-specifically bound material, a Ni-NTA super flow packed column was used to pass through and wash the crude extract supernatant first with 10 mM and then with 100 mM imidazole. Histidine-linked proteins were first eluted with 250 and then with 500 mM imidazole after

dialysis against NaCl (50 mM), HEPES (20 mM), glycerol (10%) and 1 µg/mL each of aprotinin, leupeptin and pepstatin for 120 min. The purification was performed either at 4 °C or on ice. To record autophosphorylation level, EGFR kinase assay was carried out based on DELFIA/Time-Resolved Fluorometry. The compounds were first dissolved in DMSO absolute, after dilution to appropriate concentration using HEPES (25 mM) at pH 7.4. Each compound (10 µL) was incubated with recombinant enzyme (10 µL, 5 ng for EGFR, 1:80 dilution in 100 mM HEPES) for 10 min at 25ºC, after the addition of 5X buffer (10 µL, containing 2 mM $MnCl_2$, 100 µM Na_3VO_4, 20 mM HEPES and 1 mM DTT) and ATP-$MgCl_2$ (20 µL, containing 0.1 mM ATP and 50 mM $MgCl_2$) and incubation for 1h. The negative and positive controls were included in each plate by the incubation of enzyme either with or without ATP-$MgCl_2$. The liquid was removed after incubation and the plates were washed thrice using wash buffer. Europium-tagged antiphosphotyrosine antibody (75 µL, 400 ng) was added to each well followed by incubation of 1h and then washing of the plates using buffer. The enhancement solution was added to each well and the signal was recorded at excitation and emission wavelengths of 340 at 615 nm. The autophosphorylation percentage inhibition by compounds was calculated using the following equation:

$$100\% - [(negative\ control)/(positive\ control) - (negative\ control)]$$

Using the curves of percentage inhibition of eight concentrations of each compound, IC_{50} was calculated. Majority of signals detected by antiphosphotyrosine antibody were from EGFR because the enzyme preparation contained low impurities.

4.2.2. Caspase-3 activation assay

Allow all reagents to reach room temperature before use. Gently mix all liquid

reagents prior to use. Determine the number of 8-well strips needed for the assay. Insert these in the frame(s) for current use. Add 100 µl of the *Standard Diluent Buffer* to the zero standard wells. Well(s) reserved for chromogen blank should be left empty. Add 100 µl of standards and controls or diluted samples to the appropriate microtiter wells. The sample dilution chosen should be optimized for each

experimental system. Tap gently on side of plate to mix. Cover wells with *plate cover* and incubate for 2 hours at room temperature. Thoroughly aspirate or decant solution from wells and discard the liquid, Wash wells 4 times. Pipette 100 µl of *Caspase-3 (Active) Detection Antibod*y solution into each well except the chromogen blank(s). Tap gently on the side of the plate to mix. Cover plate with *plate cover* and incubate for 1 hour at room temperature. Thoroughly aspirate or decant solution from wells and discard the liquid, Wash wells 4 times. Add 100 µl Anti-Rabbit IgG HRP Working Solution to each well except the chromogen blank(s). Prepare the working dilution as described in Preparing IgG HRP. Cover wells with the *plate cover* and incubate for 30 minutes at room temperature. Thoroughly aspirate or decant solution from wells and discard the liquid. Wash wells 4 times. Add 100 µl of *Stabilized Chromogen* to each well. The liquid in the wells will begin to turn blue. Incubate for 30 minutes at room temperature and in the dark. The incubation time for chromogen substrate is often determined by the microtiter plate

reader used. Many plate readers have the capacity to record a maximum optical density (O.D.) of 2.0. The O.D. values should be monitored, and the substrate reaction stopped before the O.D. of the positive wells exceeds the limits of the instrument. The O.D. values at 450 nm can only be read after the *Stop Solution* has been added to each well. If using a reader that records only to 2.0 O.D., stopping the assay after 20 to 25 minutes is suggested. Add 100 µl of *Stop Solution* to each well. Tap side of plate gently to mix. The solution in the wells should change from blue to yellow. Read the absorbance of each well at 450 nm having blanked the plate reader against a chromogen blank composed of 100 µl each of *Stabilized Chromogen* and *Stop Solution*. Read the plate within 2 hours after adding the *Stop Solution*. Use a curve fitting software to generate the standard curve. A four-parameter algorithm provides the best standard curve fit. Read the concentrations for unknown samples and controls from the standard curve. Multiply value(s) obtained for sample(s) by the appropriate dilution factor to correct for the dilution in step 3. Samples producing signals greater than that of the highest standard should be diluted in *Standard Diluent Buffer* and reanalyzed.

4.2.3. Caspase-8 activation assay

Cells were obtained from American Type Culture Collection, cells were grown in RPMI 1640 containing 10% fetal bovine serum at 37°C, stimulated with the compounds to be tested for caspase 8, and lysed with Cell Extraction Buffer. This lysate was diluted in Standard Diluent Buffer over the range of the assay and measured for human active caspase-8 content. (*cells are Plated in a density of 1.2 – 1.8 × 10,000 cells/well in a volume of 100µl complete growth medium + 100 ul of the tested compound per well in a 96-well plate for 24 hours before the enzyme assay for Tubulin*.). The absorbance of each microwell was read on a spectro-photometer at 450 nm. A standard curve is prepared from 7 human Caspase-8 standard dilutions and human Caspase-8 concentration determined.

4.2.4. Bax activation assay

Bring all reagents, except the human Bax-α Standard, to room temperature for at least 30 minutes prior to opening. The human Bax-α Standard solution should not be left at room temperature for more than 10 minutes. All standards, controls and samples should be run in duplicate. Refer to the Assay Layout Sheet to determine the number of wells to be used and put any remaining wells with the desiccant back into the pouch and seal the ziploc. Store unused wells at 4 °C. Pipet 100 µL of Assay Buffer into the S0 (0 pg/mL standard) wells. Pipet 100 µL of Standards #1 through #6 into the appropriate wells. Pipet 100 µL of the Samples into the appropriate wells. Tap the plate gently to mix the contents. Seal the plate and incubate at room temperature on a plate shaker for 1 hour at ~500 rpm. Empty the contents of the wells and wash by adding 400 µL of wash solution to every well. Repeat the wash 4 more times for a total of **5 washes**. After the final wash, empty or aspirate the wells and firmly tap the plate on a lint free

paper towel to remove any remaining wash buffer. Pipet 100 µL of yellow Antibody into each well, except the Blank. Seal the plate and incubate at room temperature on a plate shaker for 1 hour at ~500 rpm. Empty the contents of the wells and wash by adding 400 µL of wash solution to every well. Repeat the wash 4 more times for a total of **5 washes**. After the final wash, empty or aspirate the wells and firmly tap the plate on a lint free paper towel to remove any remaining wash buffer. Add

100 μL of blue Conjugate to each well, except the Blank. Seal the plate and incubate at room temperature on a plate shaker for 30 minutes at ~500 rpm. Empty the contents of the wells and wash by adding 400 μL of wash solution to every well. Repeat the wash 4 more times for a total of **5 washes**. After the final wash, empty or aspirate the wells and firmly tap the plate on a lint free paper towel to remove any remaining wash buffer. Pipet 100 μL of Substrate Solution into each well. Incubate for 30 minutes at room temperature on a plate shaker at ~500 rpm. Pipet 100 μL Stop Solution to each well. Blank the plate reader against the Blank wells, read the optical density at 450 nm. Calculate the average net Optical Density (OD) bound for each standard and sample by subtracting the average Blank OD from the average OD for each standard and sample. Using linear graph paper, plot the Average Net OD for each standard versus Bax concentration in each standard. Approximate a straight line through the points. The concentration of Bax in the unknowns can be determined by interpolation.

4.2.5. Bcl-2inhibition assay

Mix all reagents thoroughly without foaming before use. Wash the microwells twice with approximately 300 μL Wash Buffer per well with thorough aspiration of microwell contents between washes. Take caution not to scratch the surface of the microwells. After the last wash, empty the wells and tap microwell strips on absorbent pad or paper towel to remove excess Wash Buffer. Use the microwell strips immediately after washing or place upside down on a wet absorbent paper for not longer than 15 minutes. Do not allow wells to dry. Add 100 μL of Sample Diluent in duplicate to all standard wells and to the blank wells. Prepare standard (1:2 dilution) in duplicate ranging from 32 ng/mL to 0.5 ng/mL. Add 100 μL of Sample Diluent, in duplicate, to the blank wells. Add 80 μL of Sample Diluent, in duplicate, to the sample wells. Add 20 μL of each Sample, in duplicate, to the designated wells. Add 50 μL of diluted biotin-conjugate to all wells, including the blank wells. Cover with a plate cover and incubate at room temperature, on a microplate shaker at 100 rpm if available, for 2 hours. Remove plate cover and empty the wells. Wash microwell strips 3 times as described in step 2. Add 100 μL of diluted Streptavidin-HRP to all wells, including the blank wells. Cover with a

plate cover and incubate at room temperature, on a microplate shaker at 100 rpm if available, for 1 hour. Remove plate cover and empty the wells. Wash microwell strips 3 times as described in step 2. Proceed to the next step. Pipette 100 µl of mixed TMB Substrate Solution to all wells, including the blanks. Incubate the microwell strips at room temperature (18° to 25°C) for about 15 minutes, if available on a rotator set at 100 rpm. Avoid direct exposure to intense light. The point, at which the substrate reaction is stopped, is often determined by the ELISA reader. Many ELISA readers record absorbance only up to 2.0 O.D. Therefore, the color development within individual microwells must be watched by the person running the assay and the substrate reaction stopped before positive wells are no longer properly detectable. Stop the enzyme reaction by quickly pipetting 100 µL of Stop Solution into each well, including the blank wells. It is important that the Stop Solution is spread quickly and uniformly throughout the microwells to completely inactivate the enzyme. Results must be read immediately after the Stop Solution is added or within one hour if the microwell strips are stored at 2 - 8°C in the dark. Read absorbance of each microwell on a spectrophotometer using 450 nm as the primary wave length.

4.2.6. Cytochrome C assay

Cells were obtained from American Type Culture Collection, cells were grown in RPMI 1640 containing 10% fetal bovine serum at 37°C, stimulated with the compounds to be tested for cytochrome C, and lysed with Cell Extraction Buffer. This lysate was diluted in Standard Diluent Buffer over the range of the assay and measured for cytochrome C content. (Cells are Plated in cells/well in a volume of 100µl complete growth medium + 100 µl of the tested compound + 50 µl of 1X biotin conjugated antibody+ 100 µl of 1X streptavidin-HRP+ 100 µL TMB substrate soln of per well in a 96-well plate for 24 hours before assay).

4.3. Statistical analysis

Computerized Prism 5 program was used to statistically analyzed data using one-way ANOVA test followed by Tukey's as post ANOVA for multiple comparison at $P \leq .05$. Data were presented as mean ± SEM.

Molecular docking

Molecular docking studies were performed on promising compounds (**4a**, **4b**, and **7d**) against the crystal structure of Human EGFR (PDB ID: 1M17) as a target enzyme. *LigPrep* was used to model the synthesized compounds by creating potential enantiomers, ionization, and tautomeric states at pH = 7.0 ± 2, and then ligands were energy minimized using default OPLS3e force field parameters. The Protein Preparation Wizard was used to prepare the protein; during this procedure, crystallographic water molecules were eliminated, bond ordering and partial charges were assigned, ionization and tautomeric states of the residues were established, and H bonds were assigned. The Receptor Grid Generation tool was started by clicking on the co-crystalized ligand (4-anilinoquinazoline inhibitor Erlotinib), and the default grid box was created. The docking was done in the active site of a receptor protein, utilising the XP (extra precision) Glide simulation-based docking methodology.

.
